# Accuracy of respiratory muscle assessments to predict weaning outcomes: a systematic review and comparative meta-analysis

**DOI:** 10.1186/s13054-024-04823-4

**Published:** 2024-03-07

**Authors:** Diego Poddighe, Marine Van Hollebeke, Yasir Qaiser Choudhary, Débora Ribeiro Campos, Michele R. Schaeffer, Jan Y. Verbakel, Greet Hermans, Rik Gosselink, Daniel Langer

**Affiliations:** 1 Department of Rehabilitation Sciences, Research Group for Rehabilitation in Internal Disorders, KU Leuven, 3000 Leuven, Belgium; 2 Department of Intensive Care Medicine, University Hospitals Leuven, Leuven, Belgium; 3 Department of Internal Medicine, Ribeirão Preto Medical School, University of São Paulo (USP), Ribeirão Prêto, Brazil; 4 Department of Public Health and Primary Care, EPI-Centre, KU Leuven, Leuven, Belgium; 5 NIHR Community Healthcare Medtech and IVD Cooperative, Nuffield Department of Primary Care Health Sciences, University of Oxford, Oxford, UK; 6 Laboratory of Intensive Care Medicine, Department of Cellular and Molecular Medicine, KU Leuven, Leuven, Belgium; 7 Department of Health and Rehabilitation Sciences, Faculty of Medicine, Stellenbosch University, Stellenbosch, South Africa

**Keywords:** Predictive accuracy, Respiratory muscle assessments, Mechanical ventilator weaning, Intensive care unit

## Abstract

**Background:**

Several bedside assessments are used to evaluate respiratory muscle function and to predict weaning from mechanical ventilation in patients on the intensive care unit. It remains unclear which assessments perform best in predicting weaning success. The primary aim of this systematic review and meta-analysis was to summarize and compare the accuracy of the following assessments to predict weaning success: maximal inspiratory (PImax) and expiratory pressures, diaphragm thickening fraction and excursion (DTF and DE), end-expiratory (Tdi_ee_) and end-inspiratory (Tdi_ei_) diaphragm thickness, airway occlusion pressure (P0.1), electrical activity of respiratory muscles, and volitional and non-volitional assessments of transdiaphragmatic and airway opening pressures.

**Methods:**

Medline (via Pubmed), EMBASE, Web of Science, Cochrane Library and CINAHL were comprehensively searched from inception to 04/05/2023. Studies including adult mechanically ventilated patients reporting data on predictive accuracy were included. Hierarchical summary receiver operating characteristic (HSROC) models were used to estimate the SROC curves of each assessment method. Meta-regression was used to compare SROC curves. Sensitivity analyses were conducted by excluding studies with high risk of bias, as assessed with QUADAS-2. Direct comparisons were performed using studies comparing each pair of assessments within the same sample of patients.

**Results:**

Ninety-four studies were identified of which 88 studies (*n* = 6296) reporting on either PImax, DTF, DE, Tdi_ee_, Tdi_ei_ and P0.1 were included in the meta-analyses. The sensitivity to predict weaning success was 63% (95% CI 47–77%) for PImax, 75% (95% CI 67–82%) for DE, 77% (95% CI 61–87%) for DTF, 74% (95% CI 40–93%) for P0.1, 69% (95% CI 13–97%) for Tdi_ei_, 37% (95% CI 13–70%) for Tdi_ee_, at fixed 80% specificity. Accuracy of DE and DTF to predict weaning success was significantly higher when compared to PImax (*p* = 0.04 and *p* < 0.01, respectively). Sensitivity and direct comparisons analyses showed that the accuracy of DTF to predict weaning success was significantly higher when compared to DE (*p* < 0.01).

**Conclusions:**

DTF and DE are superior to PImax and DTF seems to have the highest accuracy among all included respiratory muscle assessments for predicting weaning success. Further studies aiming at identifying the optimal threshold of DTF to predict weaning success are warranted.

*Trial registration*: PROSPERO CRD42020209295, October 15, 2020.

**Supplementary Information:**

The online version contains supplementary material available at 10.1186/s13054-024-04823-4.

## Introduction

Failure to wean from mechanical ventilation has a complex multifactorial pathophysiology which may involve impairments of pulmonary, brain, cardiac, endocrine and respiratory muscle function [1]. Most of these factors are routinely evaluated in mechanically ventilated patients. While respiratory muscle dysfunction is highly prevalent in these patients [2–4] and is strongly associated with failing a spontaneous breathing trial (SBT), weaning failure and mortality [3–6], its assessment is not yet routinely performed in most intensive care unit (ICU) settings. Early detection of respiratory muscle dysfunction may enable clinicians to identify patients at risk of weaning failure and poor prognosis who may benefit from treatment strategies to preserve or improve respiratory muscle function [7].

Several bedside tools for a comprehensive assessment of diaphragm and non-diaphragmatic respiratory muscle function are available [8]. These tools measure distinct aspects of respiratory muscle function and vary in ease of use. The gold standard to assess diaphragm contractility is a non-volitional assessment in which twitch transdiaphragmatic pressures or (artificial) airway opening pressures are recorded during phrenic nerve stimulation [8]. In cooperative patients, assessments of the maximal voluntary transdiaphragmatic pressure can be performed [8]. These assessments however require both sophisticated equipment and technical expertise and are therefore rarely performed in clinical settings [9]. Alternative, less invasive and more clinically accessible bedside respiratory muscle assessments are available. First, global respiratory muscle strength can be assessed by measuring the maximal inspiratory (PImax) and expiratory pressures [8]. PImax is often used in research and clinical context since reference values are available and the measurement is easy to perform [10]. Second, ultrasound imaging is increasingly used in research and in clinical settings as it is a non-invasive technique performed using devices that are readily available bedside in most intensive care units. It can evaluate multiple aspects of respiratory muscle function such as thickness, contractility, and excursion of the diaphragm [8, 11]. Third, the airway occlusion pressure during the first 100 ms of inspiratory effort (P0.1) reflects the neural respiratory drive and its transmission to respiratory muscles [8, 12, 13]. P0.1 is frequently used during SBTs since it is a rapid assessment that can be easily performed with a mechanical ventilator [14]. Fourth, electromyography of respiratory muscles assesses respiratory muscle activation [8, 9]. However, this assessment has been mostly used in research since reference values are lacking and technical expertise is necessary to perform the assessments and interpret the data.

Previous meta-analyses evaluating PImax, diaphragm ultrasound assessment or P0.1 described the associations between the respiratory muscle assessment and rates of mortality and/or weaning outcomes [15–18], but none compared their predictive accuracy. Comparing these assessments provide guidance to clinicians for making a well substantiated choice between available respiratory muscle assessment methods during the weaning process.

Since there is no general agreement on an assessment for predicting weaning outcomes, it is also difficult to determine what the minimal acceptable difference in accuracy between assessments should be. For the first time, this study provides data on comparative accuracy between respiratory muscle assessments.

The aim of this systematic review and meta-analysis was therefore to estimate and compare the accuracy of bedside respiratory muscle assessments to predict weaning outcomes, focusing on assessment methods evaluated in previous meta-analyses or recommended in a recent international statement to be used in the ICU setting [8].

## Methods

### Design and search strategy

This study protocol has been registered (PROSPERO, ID: CRD42020209295) and was conducted in accordance with the Cochrane handbook for systematic reviews of diagnostic test accuracy [19, 20] and the Preferred Reporting Items for Systematic Reviews and Meta-Analyses of Diagnostic Test Accuracy Studies (PRISMA-DTA) statement [21]. In collaboration with an expert on systematic reviews from the KU Leuven Biomedical library, a comprehensive search strategy was constructed including three concepts: intensive care unit, respiratory muscles, and the assessment methods of interest: maximal respiratory pressures, ultrasound, airway occlusion pressure, twitch airway opening pressure, electromyography, transdiaphragmatic pressure and twitch transdiaphragmatic pressure.

Studies published in English from database inception until 04/05/2023 in Medline (via Pubmed), EMBASE, Web of Science, Cochrane Library and CINAHL databases were searched for these concepts, synonyms, and MeSH terms in title and abstract (See Additional file 1: Table S1 for the search string). Reference lists of included studies and published systematic reviews were additionally searched.

Given the focus on respiratory muscle assessments, we did not consider indices aggregating results from multiple assessments of functions other than respiratory muscle function.

### Study selection and data extraction

Deduplication, screening, and data extraction were performed with Covidence software (Covidence systematic review software, Veritas Health Innovation, Melbourne, Australia).

Title and abstract screening were performed by two independent reviewers for each study (YQC, TG, DRC, MRS, DP, MVH). MVH and DP resolved any conflicts. Remaining issues were resolved following discussion with senior researchers RG and DL. Peer-reviewed studies written in English were included when fulfilling all inclusion criteria: patients ≥ 18 years, accuracy to predict weaning outcomes reported and not fulfilled an exclusion criterion: animal studies, no full text available, non-peer reviewed, non-original research studies, case reports and interventional studies. The target condition was weaning success. We accepted all the various definitions of weaning from mechanical ventilation as employed in the included studies. Data extraction was performed by YQC, DP, DRC and MVH.

In case more than one threshold were evaluated for an assessment and that assessment was conducted while patients were supported with different mechanical ventilation settings, the threshold associated with mechanical ventilation settings closest to unsupported spontaneous breathing was retained.

If multiple studies reported on the same patients, multiple inclusions of the same patients were avoided by including the studies providing data on the largest sample. In case the confusion matrix (2 by 2 table) was not reported, it was computed from the provided values of sensitivity, specificity and the occurrence (prevalence) of patients presenting with and without the target condition. Studies for which the confusion matrix was reported are marked in the tables of characteristics.

Any missing data or information was requested from the corresponding authors by e-mail. If no response, two reminders were sent, or other authors of the team were contacted. In case raw data were obtained and no threshold was specified in the study, the median threshold of the other included studies reporting on the same assessment and target condition was used to compute the confusion matrix.

### Methodological quality

The methodological quality of studies included in the meta-analysis was assessed with the Quality Assessment of Diagnostic Accuracy Studies (QUADAS-2) tool [22] and evaluated by DP, DRC and MVH. A pilot was performed before assessing all studies in which reviewers developed and agreed on review-specific rating guidance (Additional file 1: Table S2). Each study was independently evaluated by two reviewers, and conflicts were resolved by the third. Remaining issues were resolved following discussion with senior researchers RG and DL.

### Statistical analyses

Results of individual studies reporting on predictive accuracy were summarized for weaning success with forest plots of sensitivity and specificity. Therefore, when studies reported on weaning failure, the confusion matrix of test accuracy was reversed. Meta-analyses were performed if at least 4 studies evaluating the accuracy of the same assessment to predict weaning success could be included [19, 20]. Predictive accuracy for each assessment was summarized as sensitivity at fixed 80% specificity with corresponding 95% confidence intervals [20, 23].

The hierarchical summary receiver operating characteristic (HSROC) model was used to estimate the SROC curves of each assessment, while accounting for different thresholds used across studies [19, 20]. HSROC meta-regression models were used to compare SROC curves between assessments included in the meta-analysis. Three meta-regression models were fitted: Model 1: “Varied,” which included covariates to allow accuracy, threshold and shape to vary for each SROC curve under comparison; Model 2: “Fixed shape,” where the covariate term for shape was removed, to assume that the SROC curves under comparison have the same shapes; Model 3: “Fixed accuracy,” where the covariate term for accuracy was removed to assume that the SROC curves under comparison have the same accuracy [20].

Potential sources of heterogeneity were investigated with meta-regression analyses. These included the condition during which the assessment was performed [i.e., SBT or during mechanical ventilation] and the threshold. Thresholds were categorized depending on the median threshold of the included studies for the respective assessment, as low or high when lower or higher than the median threshold, respectively.

Sensitivity analyses were performed: (1) after excluding studies with potential high risk of bias or applicability concerns for at least one of the domains of the QUADAS-2 tool [22], and (2) after excluding studies that conducted assessments early after start of mechanical ventilation and not during the weaning process.

Direct comparisons between assessments were performed for studies which compared each pair of assessments within the same sample of patients.

Relative diagnostic odds ratio (RDOR) was computed for each comparison between assessments (SAS macro MetaDAS). RDOR is the ratio of diagnostic odds ratios (DORs) of the compared assessments, which are summary indicators of the accuracy of each assessment [20]. An RDOR greater than 1 indicates a better performance of the first assessment compared to the second one.

SAS OnDemand for Academics (SAS Institute Inc. 2021) was used to perform the meta-analyses (SAS macro MetaDAS, by fitting the HSROC model [23]) and HSROC meta-regression analyses (using Proc NLMIXED in SAS [23]) to compare summary curves. Forest and SROC plots were created with Review manager (Review Manager, RevMan, [Computer program] Version 5.4, Copenhagen: The Nordic Cochrane Centre, The Cochrane Collaboration, 2020).

## Results

### Study selection and characteristics.

**Fig. 1:**
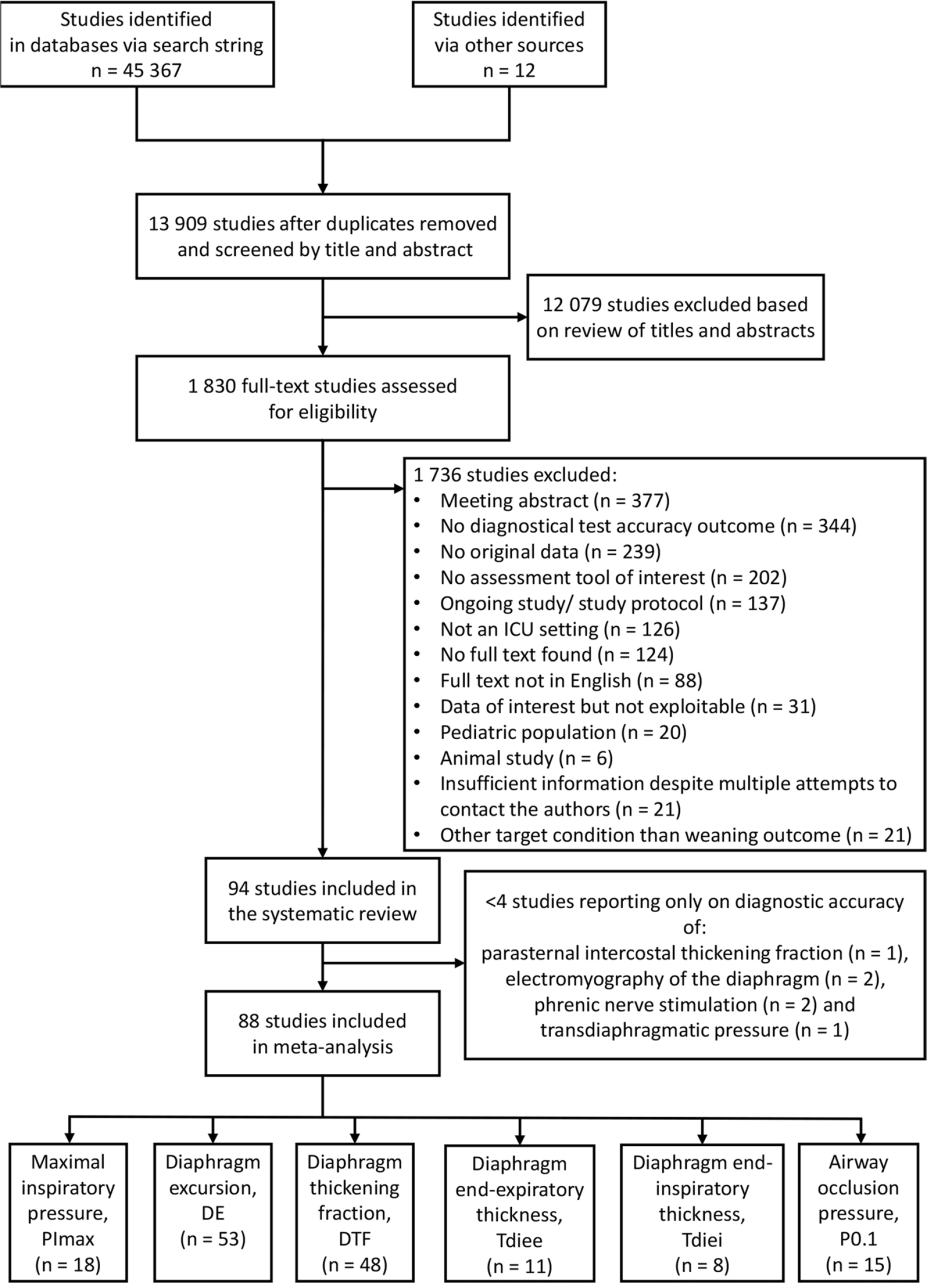
Flowchart of the studies (*n*) included in the systematic review and meta-analysis. Several studies included in the meta-analysis reported on accuracy of more than one assessment to predict weaning outcomes

The search identified 13 909 unique studies, retaining 1 830 studies for full text screening (Fig. 1). Ninety-four studies were included in the systematic review of which 88 studies reporting on accuracy of the assessments of interest to predict weaning outcomes were included in the meta-analyses (Fig. 1).

Studies included in the meta-analysis were published between 1987 and 2023. The studies were most frequently conducted in Asia (34%), followed by Europe (25%), Africa (22%), North America (12%), South America (7%) and Oceania (1%). In total, 6296 patients were included in the present meta-analyses.

Studies reporting on accuracy to predict weaning outcomes and included in the meta-analysis involved assessment of PImax (*n* = 18 studies) [24–41], diaphragm ultrasound assessments with diaphragm excursion (DE, *n* = 53 studies) [6, 26, 30, 31, 35, 37, 39–85], diaphragm thickening fraction (DTF, *n* = 48 studies) [38, 40, 43–56, 62, 65–67, 69, 71–73, 75–77, 79, 81–84, 86–101] and end-expiratory (Tdi_ee_, *n* = 11 studies) [45, 48, 53–55, 66, 73, 89, 90, 99, 100], end-inspiratory diaphragm thickness (Tdi_ei_, *n* = 8 studies) [48, 53–55, 66, 73, 89, 100] and P0.1 (*n* = 15 studies) [25, 27, 29, 36, 73, 81, 102–110] (See Additional file 1: Table S3 for study characteristics). Different weaning outcome definitions were used across studies (Additional file 1: Table S3).

Less than 4 studies reported on accuracy to predict weaning outcomes for maximal expiratory pressure [28, 32], parasternal intercostal thickening fraction [101, 111], thickness of the abdominal muscles [47], electromyography of the diaphragm [112, 113], phrenic nerve stimulation [114, 115] and transdiaphragmatic pressure [116]. Therefore the data on these assessments were not considered in the meta-analysis in case the studies were already included in the meta-analysis for other assessments [28, 32, 47, 101]. Conversely, the studies exclusively focusing on these assessments were excluded from the meta-analysis [111–116].

The characteristics of these studies and their sensitivity and specificity are presented in Additional file 1: Table S4 and Fig. S1.

### Methodological quality

**Fig. 2:**
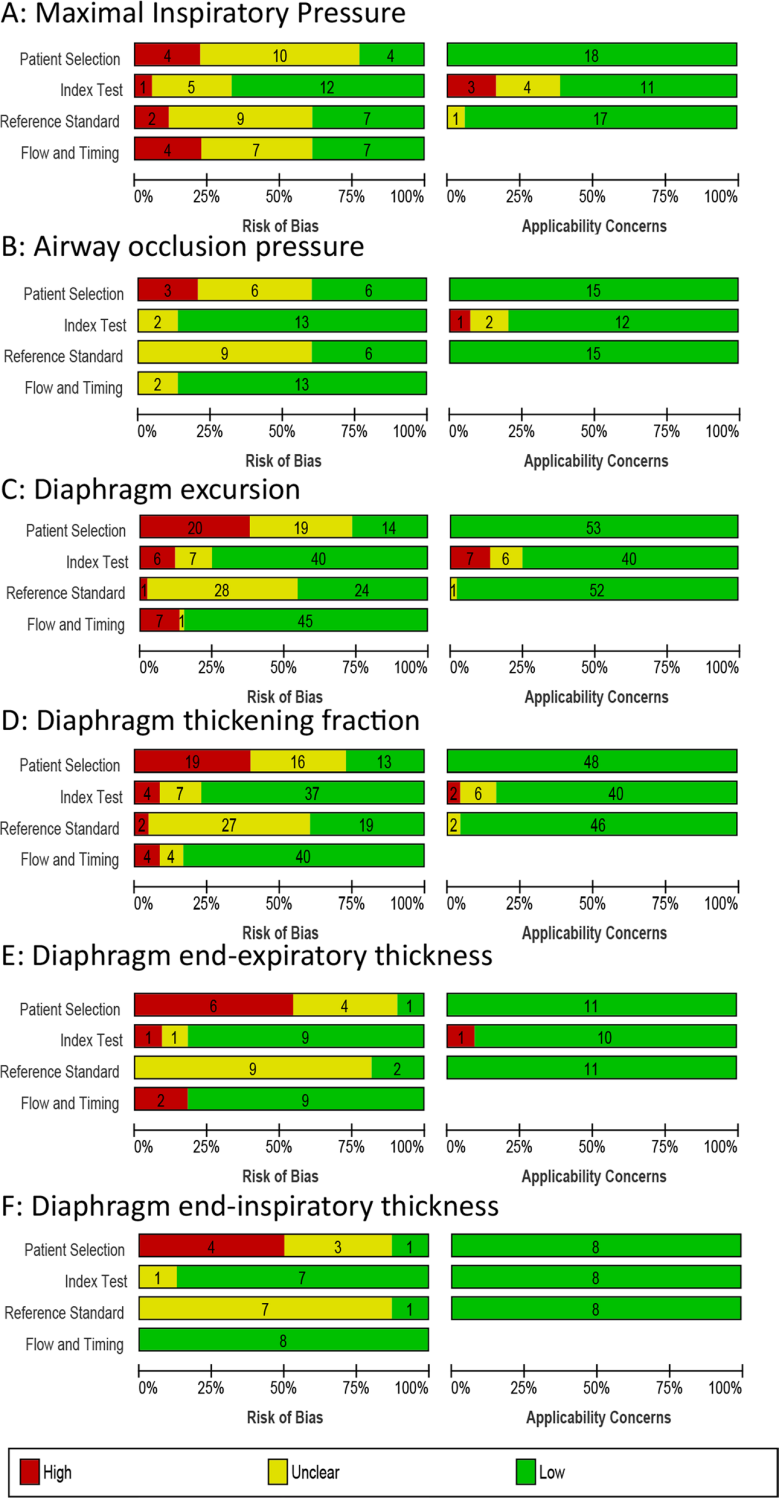
Risk of bias and applicability concerns for weaning success per assessment method. Risk of bias analyses and applicability concerns have been performed with QUADAS 2 tool for predicting weaning success by assessing the maximal inspiratory pressure (panel **A**), airway occlusion pressure, P0.1 (panel **B**), diaphragm excursion (panel **C**), diaphragm thickening fraction (panel **D**), diaphragm end-expiratory thickness (panel **E**) or diaphragm end inspiratory thickness (panel **F**)

Overview of risk of bias and applicability concerns is provided in Fig. 2. Most frequent source of potential high risk of bias involved patient selection, due to non-consecutive patient recruitment. Methodological quality per study and assessment method are provided in Additional file 1: Figs. S2–S4.

### Accuracy of respiratory muscle assessments

**Fig. 3:**
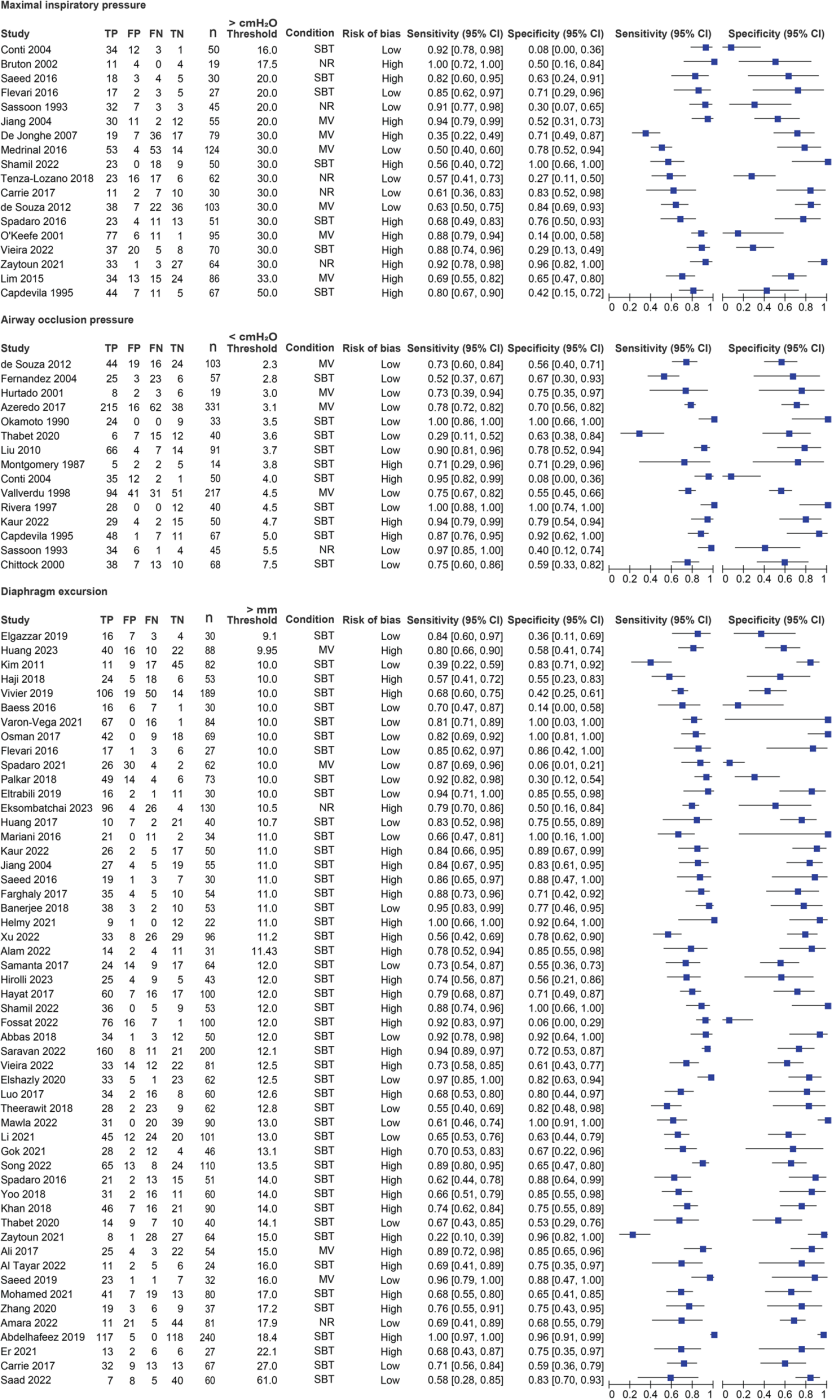
Accuracy of respiratory muscle assessment methods for predicting weaning success—part 1 of 2. Figure depicts the individual sensitivity and specificity of each study on the respiratory assessment method of interest for predicting weaning success. Condition: Indicates whether the assessment was performed while the patients was mechanically ventilated (MV) or during spontaneous breathing/spontaneous breathing trial (SBT). If no or insufficient data was provided on the condition it is marked as not reported (NR). Risk of bias: Studies that were identified as having a high risk of bias on one of the domains of the QUADAS 2 tool were indicated as high risk of bias (High). Studies which had no domain in which a potential high risk of bias was identified were indicated as low risk of bias (Low). Abbreviations: *TP* True positive, *FP* False positive, *FN* False negative, *TN* True negative, *n* Sample size of the study, *SBT* Spontaneous breathing trial, *MV* Mechanical ventilation, *NR* Not reported, *CI* Confidence interval

**Fig. 4:**
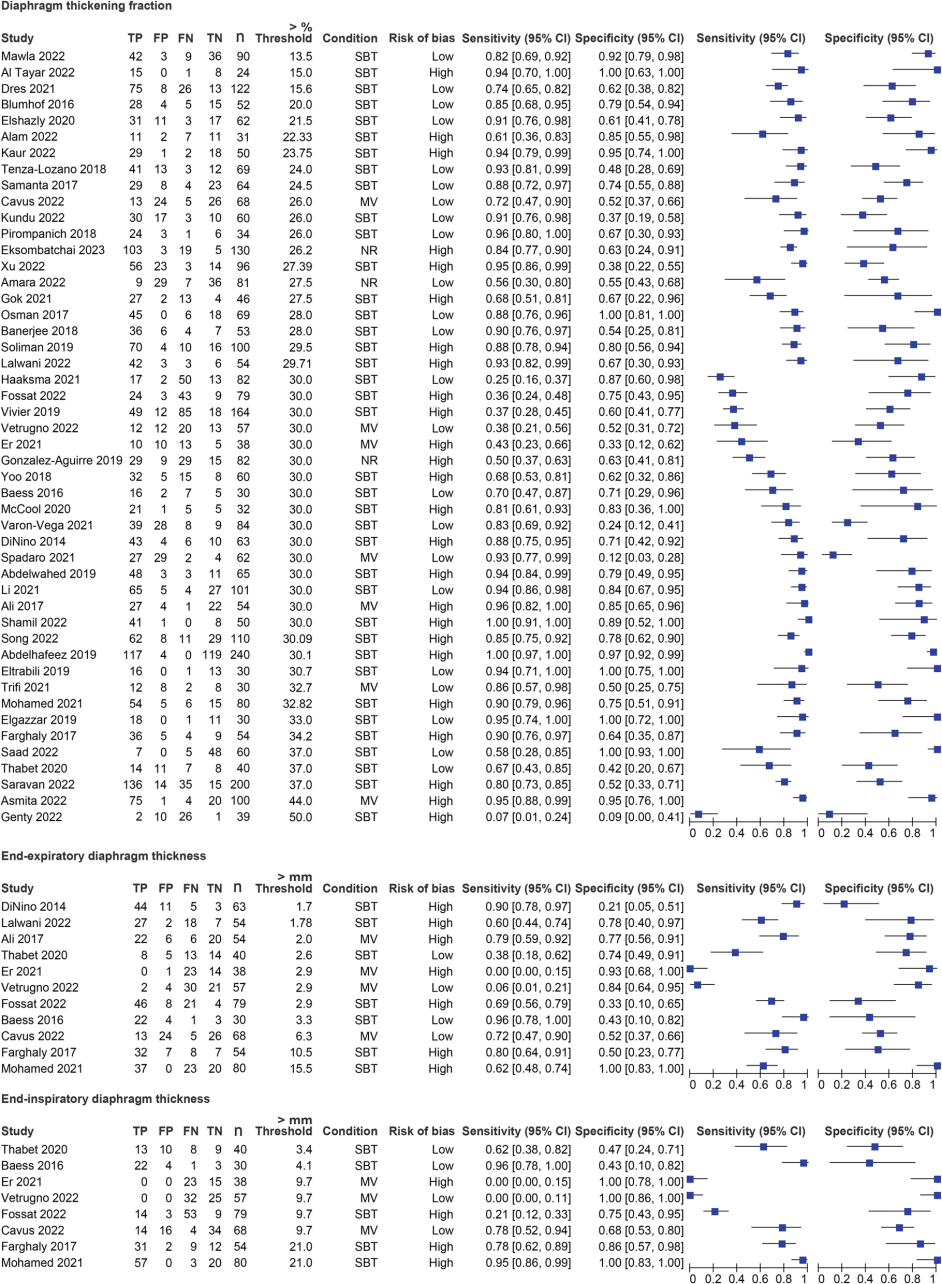
Accuracy of respiratory muscle assessment methods for predicting weaning success—part 2 of 2. Figure depicts the individual sensitivity and specificity of each study on the respiratory assessment method of interest for predicting weaning success. Condition: Indicates whether the assessment was performed while the patients was mechanically ventilated (MV) or during spontaneous breathing/spontaneous breathing trial (SBT). If no or insufficient data was provided on the condition it is marked as not reported (NR). Risk of bias: Studies that were identified as having a high risk of bias on one of the domains of the QUADAS 2 tool were indicated as high risk of bias (High). Studies which had no domain in which a potential high risk of bias was identified were indicated as low risk of bias (Low). Abbreviations: *TP* True positive, *FP* False positive, *FN* False negative, *TN* True negative, *n* Sample size of the study, *SBT* Spontaneous breathing trial, *MV* Mechanical ventilation, *NR* Not reported, *CI* Confidence interval

Figures 3 and 4 provide the values of sensitivity and specificity of individual studies. At a fixed value of 80% for specificity to predict weaning failure, the estimated values of sensitivity were 63% (95% CI 47–77%) for PImax, 75% (95% CI 67–82%) for DE, 77% (95% CI 61–87%) for DTF, 74% (95% CI 40–93%) for P0.1, 69% (95% CI 13–97%) for Tdi_ei_, 37% (95% CI 13–70%) for Tdi_ee_. Comparison of these tests showed that the accuracy for predicting weaning success was statistically significantly higher for DE versus PImax (*p* = 0.04) and for DTF versus PImax (*p* < 0.01) (Table 1). The curves estimated with the HSROC model for each assessment method are provided in Additional file 1: Fig. S5. The results on one-to-one comparisons of SROC curves included in the meta-analyses, are presented in Table 1.

**Table 1:** Comparisons of SROC curves to predict WS between respiratory muscle assessments

Assessment comparison	Studies (*n*)	Patients (*n*)	− 2Log Likelihood values of the fitted models			Likelihood ratio assessments			RDOR (95% CI)
			Varied model (V)	Fixed shape model (FS)	Fixed accuracy model (FA)	Model comparison	Chi-square (*df* = 1)	*p* value	
DE versus PImax	53	3638	857.9	858.2	862.3	FS versus V	0.3	0.58	**2.11 (1.14–3.93)**
	18	1107				FA versus FS	4.1	**0.04**	
DTF versus Pimax	48	3471	798.9	799.3	811.6	FS versus V	0.4	0.53	**4.70 (1.71–12.96)**
	18	1107				FA versus FS	12.3	** < 0.01**	
DE versus DTF	53	3638	1280.7	1306.7	1306.7	FS versus V	26	** < 0.01**	¥
	48	3471				FA versus FS	¥	¥	
DE versus Tdi_ee_	53	3638	757.4	765.3	769.6	FS versus V	7.9	** < 0.01**	¥
	11	617				FA versus FS	¥	¥	
DTF versus Tdi_ee_	48	3471	774.1	774.1	775.6	FS versus V	0	1	1.46 (0.85–2.50)
	11	617				FA versus FS	1.5	0.22	
Pimax versus Tdi_ee_	18	1107	336.3	338.8	340	FS versus V	2.5	0.11	1.85 (0.64–5.37)
	11	617				FA versus FS	1.2	0.27	
Pimax versus P0.1	18	1107	380.6	382.5	383.4	FS versus V	1.9	0.17	0.68 (0.30–1.54)
	15	1225				FA versus FS	0.9	0.34	
DE versus P0.1	53	3638	782.9	787.1	787.1	FS versus V	4.2	**0.04**	¥
	15	1225				FA versus FS	¥	¥	
DTF versus P0.1	48	3471	746.6	748.4	749	FS versus V	1.8	0.18	1.49 (0.50–4.44)
	15	1225				FA versus FS	0.6	0.44	
P0.1 versus Tdi_ee_	15	1225	312	312.1	312.7	FS versus V	0.1	0.75	1.87 (0.51–6.81)
	11	617				FA versus FS	0.6	0.44	

To compare SROC curves, the following HSROC models were compared: Model 1 (“Varied”, V) which includes covariates to allow accuracy, threshold and shape to vary by assessment; Model 2 (“Fixed shape”, FS) from which the covariate term for shape was removed, to assume that the SROC curves under comparison have the same shapes; Model 3 (“Fixed accuracy”, FA) from which also the covariate term for accuracy was removed to assume that the SROC curves under comparison have the same accuracy. A significant *p* value for Model 2 versus 1 denotes that the shapes of the SROC curves under comparison are different; a significant *p* value for Model 3 versus 2 comparison denotes that the overall accuracies of the assessments under comparison are different. ¥ = in case of a significant *p* value for Model 2 versus 1, which denotes that the shapes of the SROC curves under comparison are different, further comparisons of Model 3 versus 2 was not performed. Significance level: *p* < 0.05. Values of RDOR were calculated as relative diagnostic odds ratio of the first assessment compared to the second, as indicated by the order specified in the “Assessment comparison” column. A value of the RDOR higher or lower than 1 indicate that the first assessment has higher or lower accuracy than the second one. Confidence intervals of RDOR not containing 1 indicate significant higher or lower accuracy. Comparisons were not possible for Tdi_ee_ and Tdi_ei_ due to irregular shape of the SROC curves. Significant p-values (p<0.05) and RDOR 95%CI are highlighted in bold. Abbreviations: CI Confidence interval, DE Diaphragm excursion, DTF Diaphragm thickening fraction, PImax Maximal inspiratory pressure, P0.1 Airway occlusion pressure, Tdiee Diaphragm thickness at end-expiration, df  Degrees of freedom which are equal to the difference in the number of parameters between the models that are compared, RDOR Relative diagnostic odds ratio, WS Weaning success. Bold value indicates statistically significant values

### Heterogeneity analyses

The condition of the assessments (i.e., during mechanical ventilation or during an SBT) and the chosen threshold were t identified as a source of heterogeneity for PImax, DE, DTF, P0.1 (Additional file 1: Tables S5, S6).

### Sensitivity analyses

Sensitivity analyses were performed based on a total of 44 studies after exclusion of studies with high potential risk of bias from the meta-analyses. The count included studies that reported results for multiple assessments only once. At a fixed value of 80% for specificity to predict weaning failure, the estimated values of sensitivity were 61% (95% CI 44–75%) for PImax, 78% (95% CI 63–88%) for DTF and 76% (95% CI 64–85%) for DE. SROC curves of PImax (*n* = 7 studies) [26, 27, 29, 30, 33, 36, 38], DTF (*n* = 23 studies) [38, 47–52, 62, 65, 67, 69, 71–73, 75, 88, 89, 93, 94, 96, 98, 101], DE(*n* = 23 studies) [6, 26, 30, 42, 47–52, 60, 62, 64, 65, 67–75] and P0.1 (*n* = 11 studies) [29, 36, 73, 102–106, 108–110], Tdi_ee_ (*n* = 4 studies) [48, 73, 89, 100] and Tdi_ei_ (*n* = 4 studies) [48, 73, 89, 100] were estimated (Additional file 1: Fig. S6 and Table S7). The one-to-one comparisons showed that accuracy for predicting weaning success was significantly higher for DTF versus DE (Additional file 1: Table S8).

Results of SROC comparisons after exclusion of studies that conducted assessments early after start of mechanical ventilation [53, 98] and not during the weaning process did not substantially differ from the results presented in Table 1 (Additional file 1: Table S9).

### Direct comparisons

At least 4 direct comparative studies reporting on accuracy to predict weaning success were found for the following pairs of assessments: DE versus PImax (*n* = 8 studies) [30, 31, 35, 37, 39–41], DE versus DTF (*n* = 28 studies) [40, 43–45, 47–52, 54, 56, 62, 65–67, 69, 71–73, 75, 77, 79, 81–84, 117], DE versus Tdi_ee_ (*n* = 5 studies) [45, 48, 54, 66, 73], DE versus Tdi_ei_ (*n* = 4 studies) [48, 54, 66, 73], DTF versus Tdi_ee_ (*n* = 11 studies) [45, 48, 53–55, 66, 73, 89, 90, 99, 100], DTF versus Tdi_ei_ (*n* = 8 studies) [48, 54, 55, 66, 73, 89, 100] and P0.1 versus PImax (*n* = 4 studies) [25, 27, 29, 36]. The SROC curves estimated based on direct comparative studies are presented in Additional file 1: Fig. S7 (DE vs. DTF) and in Additional file 1: Fig. S8 (DE vs. PImax, DE vs. Tdi_ee_, DE vs. Tdi_ei_, Tdi_ee_ vs. DTF, Tdi_ei_ vs. DTF and P0.1 vs. PImax).

At a fixed value of 80% for specificity to predict weaning failure, the estimated values of sensitivity were 88% (95% CI 78–93%) for DTF and 79% (95% CI 68–87%) for DE. The results of the one-to-one comparisons of SROC curves of the identified paired of assessments showed that predictive accuracy was higher for DTF versus DE (*p* < 0.01, Table 2).

**Table 2:** Comparisons of SROC curves for prediction of WS based on direct comparative studies

Assessment comparison	Studies (*n*)	Patients (*n*)	− 2Log Likelihood value of the fitted models			Likelihood ratio assessments			RDOR (95% CI)
			Varied model (V)	Fixed shape model (FS)	Fixed accuracy model (FA)	Model comparison	Chi-square (*df* = 1)	*p* value	
DE versus PImax	8	377	190.4	193.5	194.8	FS versus V	3.1	0.08	1.51 (0.39–5.84)
	8	377				FA versus FS	1.3	0.25	
DTF versus DE	28	2081	649.3	650.9	664.5	FS versus V	1.6	0.20	1.84 (1.25–2.70)
	28	2081				FA versus FS	13.6	**< 0.01**	
DE versus Tdi_ee_	5	258	110.4	111.3	111.3	FS versus V	0.9	0.34	0.74 (0.14–3.80)
	5	258				FA versus FS	0	1	
Pimax versus P0.1	4	265	83	84.3	84.7	FS versus V	1.3	0.25	0.59 (0.02–18.80)
	4	265				FA versus FS	0.4	0.53	

Direct comparisons were made only using data from studies which compared each pair of assessments on the same patients. To compare SROC curves, the following HSROC models were compared: Model 1 (“Varied”, V) which includes covariates to allow accuracy, threshold and shape to vary by assessment; Model 2 (“Fixed shape”, FS) from which the covariate term for shape was removed, to assume that the SROC curves under comparison have the same shapes; Model 3 (“Fixed accuracy”, FA) from which also the covariate term for accuracy was removed to assume that the SROC curves under comparison have the same accuracy. Significance level: *p* < 0.05. Values of RDOR were calculated as relative diagnostic odds ratio of the first assessment compared to the second, as indicated by the order specified in the “Assessment comparison” column. A value of the RDOR higher or lower than 1 indicates that the first assessment has higher or lower accuracy than the second one. Confidence intervals of RDOR not containing 1 indicate significant higher or lower accuracy. Comparisons of the SROC curves were not performed for DTF versus Tdi_ee_ and DTF versus Tdi_ei_ because of the irregular shape of one of the curves and for DE versus Tdi_ei_ because model FS could not be fitted. Significant p-values (p<0.05) and RDOR 95%CI are highlighted in bold. Abbreviations: CI Confidence interval, DE Diaphragm excursion, DTF Diaphragm thickening fraction, PImax Maximal inspiratory pressure, P0.1 Airway occlusion pressure, Tdiee Diaphragm thickness at end-expiration, df Degrees of freedom which are equal to the difference in the number of parameters between the models that are compared, WS Weaning success. Bold value indicates statistically significant values

## Discussion

### Main findings

This systematic review and meta-analysis aimed to estimate and compare the accuracy of multiple bedside respiratory muscle assessments to predict weaning outcomes in critically ill patients. The estimated values of sensitivity were 63% for PImax, 75% for DE, 77% for DTF, 74% P0.1, 69% for Tdi_ei_, and 37% for Tdi_ee_ at 80% specificity for predicting weaning success. DTF and DE performed significantly better than PImax, with DTF showing the highest accuracy in direct comparative studies. Our findings indicate that among the evaluated bedside respiratory muscle assessments, DTF is the most accurate tool to identify mechanically ventilated patients who may be successfully weaned.

### Accuracy of respiratory muscle assessment methods to predict weaning outcomes

Our findings confirm the results of previous studies when considering all the studies included in our meta-analysis for DTF, DE and PImax to predict weaning outcomes [15, 16, 18, 118]. In fact, when visually inspecting the estimated SROC curves in previous meta-analyses [15, 16, 18], values of sensitivity to predict weaning success at a fixed value of 80% specificity were between 70 and 80% for DE and DTF and close to 60% for PImax.

Importantly, our study provides new valuable information on accuracy differences between respiratory muscle assessment methods to predict weaning success: 1) DTF and DE are more accurate than PImax; 2) DTF has higher accuracy than DE when excluding studies with potential high risk of bias or considering direct comparative studies.

DTF and DE showed higher accuracy to predict weaning success compared to PImax when considering all the studies in the meta-analysis. However, these differences were no longer statistically significant in sensitivity analyses despite the unchanged magnitude of difference in sensitivity at 80% specificity (sensitivity ranging from 75 to 78% for DTF and DE and from 61 to 63% for PImax). Additionally, no study directly compared DTF with PImax and the SROC curves estimated based on only eight direct comparative studies of DE versus PImax showed minimal sensitivity differences at 80% specificity. These findings are likely due to result variability and the limited number of studies reporting on PImax or directly comparing PImax with DTF and DE. The use of different assessment protocols, such as a 20–30 s occlusion method [119] or a single complete expiration followed by a forceful inspiration against a closed valve [120] likely contributed to the variability in predictive accuracy of PImax.

Hence, it remains plausible that DE and DTF would have shown a higher accuracy than PImax in sensitivity and direct comparison analyses if a larger number of studies similar to the number used in the overall meta-analysis had been available.

The superiority of DTF over DE may be explained by the influence of mechanical ventilation support, patient’s positioning, and variation in thoracic and abdominal pressures on the interpretation of DE [18, 121].

Although P0.1 is frequently used during SBTs due to its rapid assessment using a mechanical ventilator, published data on its predictive accuracy are few and widely variable. The variability of predictive accuracy of P0.1 may be due to variations in P0.1 formulas across mechanical ventilator brands [12, 14] or to the use of an external device for measurement.

The paucity of data also hindered the summarization and interpretation of predictive accuracy of Tdi_ei_ and Tdi_ee_.

### Strengths and weaknesses

To our knowledge, this is the largest systematic review and meta-analysis to summarize and the first to formally compare accuracy of several bedside respiratory muscle assessments to predict weaning success in critically ill patients using indirect and direct comparative studies and sensitivity analyses excluding studies with potentially high risk of bias. Through a comprehensive search string across diverse databases and the retrieval of missing data from authors, we obtained a maximum of published data.

Our study has limitations. Direct comparisons, which are considered as more reliable and less likely to be biased compared to indirect comparisons [20, 122], could not be performed for all the assessment methods of interest. Another limitation is that no estimation of predictive accuracy could be carried out for all the assessment methods of interest for our review due to limited available data. Moreover, deriving the confusion matrix of studies for which we received individual (raw) patient data by using the median threshold from other included studies reporting on the same assessment method could potentially introduce bias. However, we deemed this approach logical in the absence of a consensus on threshold values to predict weaning outcomes.

The use of different thresholds across the included studies introduces limitations to this work. Despite using the HSROC model as recommended by the Cochrane Handbook [19, 20], this approach did not allow for determining the most optimal threshold to predict weaning success for each assessment method.

Furthermore, most of the studies selected thresholds post-hoc relying on assessments at a single point in time and using the Youden index (i.e., sensitivity + specificity − 1), leading to potential overestimation of the sensitivity and specificity of the assessment method [123] and timing of testing may influence the capability of an assessment method to predict weaning outcomes.

Finally, patient heterogeneity may have also influenced the prediction characteristics of the assessment methods considered in this study.

### Implications for clinical practice

A spontaneous breathing trial is a recommended for assessing whether a patient’s readiness for mechanical ventilator weaning [124]. Previous studies reported a 10 to 20% weaning failure rate among patients who passed the trial [125]. Respiratory muscle assessment methods are promising tools to further assist clinician at the bedside during the weaning process.

This meta-analysis supports the use of DE and DTF over PImax to predict weaning outcomes in mechanically ventilated patients. Further sensitivity analyses suggest that DTF may outperform DE.

PImax and P0.1 are accessible tools in the hands of clinicians, but the results of this meta-analysis and the greater variability in their sensitivity to predict weaning success compared to DTF and DE, are not currently supporting their use to predict weaning success.

In contrast to PImax and P0.1, the need for specific training to learn diaphragm ultrasonography has been recently highlighted [126]. Although DTF measurements may have a slower learning curve than DE [126], there is indication that clinicians previously lacking experience can produce accurate measurements when compared to measurements performed by experts and that a good intra-rater and inter-rater agreement among assessors can be achieved after a relatively brief training [96, 127]. Moreover, ICU allied healthcare professionals can also easily acquire the skills required for diaphragm ultrasonography assessment. Among them, respiratory physiotherapists are becoming very involved in the applications of thoracic ultrasound (including diaphragm) imaging in their clinical practice [128–131]. They can certainly support physicians during the weaning process by performing DE and DTF measurements, which can be rapidly performed even in uncooperative patients without causing discomfort or prolonging the weaning process. Additionally, DTF assessments have moderate reproducibility [132].

Subsequently, we encourage integrating DTF assessments during SBTs after specific training [126] and following the recently published recommendations on methodology for diaphragm ultrasonography [126]. However, despite the potential of DTF to guide the weaning process, to date only one single center study observed that incorporating DTF information in patients with a DTF > 30% significantly reduced the time to extubation [95]. Therefore more future prospective studies are needed to investigate its impact on clinical decision making and improvement of weaning outcomes.

Finally, most included studies used thresholds ranging from 25 to 33% for DTF (Fig. 4). We recommend using that range of thresholds for DTF in clinical practice to predict weaning outcomes.

### Implications for research

Additional high-quality test accuracy studies comparing predefined thresholds and multiple respiratory muscle assessments within the same patient sample are needed to find optimal threshold values for predicting weaning outcomes, thus increasing their clinical usefulness and routine applicability. More homogeneity of weaning definitions can facilitate the interpretation and applicability of studies reporting on predictive accuracy of respiratory muscle assessment methods. A recent promising weaning definition is available and may be used to account for tracheostomized patients in future studies [133]. Notably, although P0.1 is a very fast and easy tool to use, data on its accuracy to predict weaning outcomes are lacking, warranting future research to further establish its accuracy and the optimal cut-off.

Methodology of ultrasound assessments varied in mode, probe type, probe and patient positioning and breathing condition. Clear reporting and uniformity in the methodology based on recent recommendations [126] will ensure reproducibility of predictive accuracy in research and clinical practice.

Exploring comparative accuracy of different combinations of multiple assessments of respiratory muscles would be important to determine the most accurate combination for predicting weaning outcomes. DE and DTF are promising assessments and accuracy may improve when their evaluation is combined which can be done efficiently using the same equipment.

Finally, successful weaning depends on various factors beyond respiratory muscle function, such as cardiac and respiratory failure, cognitive and endocrine dysfunction [1]. Machine learning, incorporating all these facets may be a powerful tool to predict weaning success [134] and diaphragm thickening fraction is a parameter that deserves inclusion in models for future evaluations [134].

## Conclusions

Among several bedside respiratory muscle assessments, diaphragm thickening fraction and excursion have higher accuracy compared to maximal inspiratory pressure to predict weaning success. Predictive accuracy seems to be the highest when diaphragm thickening fraction is assessed. This assessment has a great potential to assist clinicians during weaning. It can be applied by clinicians lacking experience after specific training, even in non-cooperative patients, without causing discomfort.

Future research should validate the use of ultrasound assessments when incorporated in clinical decision-making around weaning and explore the accuracy of combining ultrasound with other respiratory muscle assessments. In addition, identifying the optimal threshold for diaphragm thickening fraction to predict weaning success would be of great clinical and research value. Lastly, it is worthy to investigate the potential of combining several bedside respiratory muscle assessments or of multifactorial models to predict weaning outcomes.

### Supplementary Information

**Additional file 1:** Search string, characteristics tables of the studies, review-specific risk of bias rating guidance, supplementary figures and tables.

## Data Availability

The dataset used and/or analyzed for the current study is available from the corresponding author on reasonable request.

## Abbreviations

AECOPD: Acute exacerbation chronic obstructive pulmonary disease
ARF: Acute respiratory failure
ATS: American Thoracic Society
CCU: Coronary care unit
COPD: Chronic obstructive pulmonary disease
CPAP: Continuous positive airway pressure
DE: Diaphragm excursion
DTF: Diaphragm thickening fraction
DOR: Diagnostic odds ratio
EICU: Emergency intensive care unit
ERS: European Respiratory Society
FRC: Functional residual capacity
HSROC: Hierarchical Summary Receiver Operating Characteristic
ICU: Intensive care unit
MICU: Medical intensive care unit
MV: Mechanically ventilated/mechanical ventilation
NICU: Neurointensive care unit
NIV: Non-invasive ventilation
NIPPV: Non-invasive positive pressure ventilation
NR: Not reported
P0.1: Airway occlusion pressure
PEEP: Positive end-expiratory pressure
PImax: Maximal inspiratory pressure
PRISMA-DTA: Preferred Reporting Items for Systematic Reviews and Meta-analyses of Diagnostic Accuracy Studies
PS: Pressure support
PSV: Pressure support ventilation
RDOR: Relative diagnostic odds ratio
QUADAS-2: Quality Assessment of Diagnostic Accuracy Studies
RICU: Respiratory intensive care unit
RV: Residual volume
SICU: Surgical intensive care unit
SB: Spontaneous breathing
SBT: Spontaneous breathing trial
Tdi_ee_: End-expiratory diaphragm thickness
Tdi_ei_: End-inspiratory diaphragm thickness
TLC: Total lung capacity
