# Supplementary material for: Accuracy of respiratory muscle assessments to predict weaning outcomes: a systematic review and comparative meta-analysis
Source: Crit Care. 2024 Mar 7;28:70. doi: 10.1186/s13054-024-04823-4 (PMC10919035; doi:10.1186/s13054-024-04823-4)
Supplement: Supplementary file 1 — Additional file 1: Search string, characteristics tables of the studies, review-specific risk of bias rating guidance, supplementary figures and tables. [file 13054_2024_4823_MOESM1_ESM.docx]

Additional File 1

**Accuracy of respiratory muscle assessments to predict weaning outcomes: a systematic review and comparative meta-analysis.**

**Authors**

Diego Poddighe*, Marine Van Hollebeke*, Yasir Qaiser Choudhari, Débora Ribeiro Campos, Michele R Schaeffer, Jan Verbakel, Greet Hermans, Rik Gosselink, Daniel Langer

*Shared first author

**Table of contents**

[Table S1. Search string for systematic literature review 3](#_Toc156835460)

[Table S2. Consensus reached among reviewers on review-specific rating guidance using QUADAS-2 tool 4](#_Toc156835461)

[Table S3. Characteristics table of studies investigating predictive accuracy of assessment methods to predict weaning success. 7](#_Toc156835462)

[Table S4. Characteristics table of studies investigating predictive accuracy of the assessment methods not included in the meta-analyses 39](#_Toc156835463)

[Figure S1. Accuracy of respiratory muscle assessment methods for predicting weaning success 44](#_Toc156835464)

[Figure S2. Risk of bias and applicability concerns for weaning success per assessment method, part 1 of 3 45](#_Toc156835465)

[Figure S3. Risk of bias and applicability concerns for weaning success per assessment method, part 2 of 3 46](#_Toc156835466)

[Figure S4. Risk of bias and applicability concerns for weaning success per assessment method, part 3 of 3 47](#_Toc156835467)

[Figure S5. Estimated summary receiver operating characteristic (SROC) curves to predict WS 48](#_Toc156835468)

[Figure S6. Estimated SROC curves to predict WS after exclusion of studies with high risk of bias 48](#_Toc156835469)

[Figure S7. Estimated SROC curves to predict WS considering direct comparative studies 49](#_Toc156835470)

[Figure S8. Estimated SROC curves to predict weaning success based on direct comparative studies 49](#_Toc156835471)

[Table S5. Within-assessment SROC curve comparisons with timepoint as potential source heterogeneity. 50](#_Toc156835472)

[Table S6. Within-assessment SROC curve comparisons with threshold as potential source of heterogeneity. 51](#_Toc156835473)

[Table S7. Estimated HSROC parameters after exclusion of studies with high RoB 51](#_Toc156835474)

[Table S8. Comparisons of SROC curves to predict WS after excluding studies with high risk of bias. 52](#_Toc156835475)

[Table S9. SROC curves comparisons after excluding studies conducting assessments early after start of MV 53](#_Toc156835476)

[References 54](#_Toc156835477)

| Table S1. Search string for systematic literature review | | | | |
| --- | --- | --- | --- | --- |
| **Concepts** | **Mesh term** | **Title and abstract** | | |
| **Critically ill and mechanically ventilated patients** | Intensive Care Units  Respiratory Care Units  Critical Care  Critical Illness  Ventilators, Mechanical  Continuous Positive Airway Pressure  Intermittent Positive-Pressure Breathing  Intermittent Positive-Pressure Ventilation  Ventilator Weaning  Airway Extubation | intensive-care  ICU  ICUs  Respiratory care unit*  RCU  Critical care  critically ill  critically illness  critical ill | critical illness  mechanical-ventilat*  mechanically-ventilat*  respirator  respirators  MV  Continuous positive airway pressure  CPAP | IPPB  Inspiratory Positive Pressure  Intermittent-Positive-Pressure-Ventilation  IPPV  Weaning  GICU  extubation*  detubation* |
| **Respiratory muscle assessments** | PImax/PEmax:  Maximal Respiratory Pressures  Respiratory Function Tests  Ultrasound:  Ultrasonography  Electromyography:  Electromyography  Phrenic nerve stimulation:  Phrenic Nerve  Neuromuscular Monitoring  Evoked Potentials, Motor  Evoked Potentials  Electric Stimulation  Neural Conduction  Transdiaphragmatic pressure:  Pressure  Airway occlusion pressure:  Airway Resistance | PImax/PEmax:  Maximal respiratory pressure*  Maximal inspiratory pressure*  Maximal expiratory pressure*  PImax  MIP  PEmax  MEP  Respiratory Function  Ultrasound:  Ultraso*  Echotomogra*  Medical-Sonogra*  US  DUS  echogra*  gray-scale-echography  Electromyography:  Electromyogra*  EMG  myogra*  Phrenic nerve stimulation:  Phrenic-Nerve*  neuromuscular-monitoring  Motor-Evoked-Potential*  Electric-Stimulation*  Magnetic-stimulation* | Neural-Conduction*  Nerve-Conduction*  diaphragm-innervation  nerve-stimulation  nerve-cell-stimulation  nervus-phrenicus  axon-conduction  nerve-conductivity  nerve-fiber-conduction  nerve-transmission  nervous-conduction  neuroconduction  neuron-conduction  motoneuron-conduction  motor-conductivity  Transdiaphragmatic pressure:  Transdiaphragma*  twPdi  Pdi  endotracheal tube pressure*  endotracheal pressure*  oesophageal pressure*  esophageal pressure*  gastric pressure*  abdominal pressure*  pleural pressure*  endoesophageal pressure | intraesophageal pressure  intraoesophageal pressure  oesophagus pressure  esophagus pressure  lung pressure  lung-interstitial-pressure  pulmonary-pressure  trachea-pressure  intratracheal-pressure  stomach-pressure  gastric-pressure  intragastric-pressure  abdomen-pressure  intraabdominal-pressure  intrathoracic-pressure  Airway occlusion pressure:  Airway-resistance*  airway-occlusion*  P0.1  airway-pressure  occlusion-pressure  airway-closure  transpulmonary-pressure  intrapleural-pressure  gastroesophageal-pressure  mouth-pressure* |
| **Respiratory muscles** | Respiratory Muscles  Diaphragm  Intercostal Muscles  Abdominal Muscles  Abdominal Oblique Muscles  Abdominal Wall  Rectus Abdominis | respiratory-muscle*  ventilatory-muscle*  diaphragm*  intercostal-muscle*  abdominal-muscle*  transverse-abdominal  oblique-muscle*  abdominal-wall  recti-abdominis  rectus-muscle* | scalene-muscle*  scalenus-muscle  scaleni  scalene  sternocleidomastoid*  thorax-muscle*  chest-muscle*  thoracic-muscle*  breathing-muscle* | airway-muscle  hemidiaphragm*  abdomen-muscle*  rectus-sheath  sternomastoid  PTP  pressure-time-product  WOB  work-of-breathing |
| Mesh terms and title-abstract terms within each concept were stringed with “OR” command and the 3 concepts were stringed together with AND command | | | | |

#

| Table S2. Consensus reached among reviewers on review-specific rating guidance using QUADAS-2 tool | | | |
| --- | --- | --- | --- |
|  | **Risk of Bias** | **Applicability** |  |
| **Domain 1: Patient selection** | Q1: Was a consecutive or random sample of patients enrolled? In case it was not specified that the sample was “*consecutive*”, “*random*” or clearly mentioned that all patients from a specified period were enrolled, we marked it as unclear.  Q2: Was a case-control design avoided?  This signalling question did not need further clarification.  Q3: Did the study avoid inappropriate exclusions? The following criteria were not considered as inappropriate:   - Exclusion due to low ultrasound imaging quality. (i.e: obese patients) - Tracheotomised patients when reference standard is weaning outcome. As definition of weaning outcome varies between the studies, we cannot base our interpretation on the appropriateness of excluding tracheotomised patients on the definition that was used for the weaning outcome. Therefore we marked this exclusion as an inappropriate exclusion however not an important inappropriate exclusion.   The following criteria were considered as inappropriate exclusions:   - *Varon-Vega 2019[1]*: Neuromuscular blocking agents during admission to the intensive care unit. - *McCool 2020[2]*: Prolonged mechanical ventilated patients. - *Farghaly 2017[3]*: Previous cardiothoracic surgery. - *González-Aguirre 2019[4]*: Patients with previous extubation failure. - *Ali 2017[5]:* Use of non-invasive ventilation before the start of mechanical ventilation. - *Luo 2017[6]*: Severe mitral stenosis or prosthetic mitral valve*.*  \| **Domain 1: Interpretation of signaling questions (Q)** \| \| \| \| \| \| --- \| --- \| --- \| --- \| --- \| \| **Q1** \| **Q2** \| **Q3** \| **Risk of bias** \| \| Yes \| Yes \| Yes / Unclear / No \| Low \| \| Unclear \| Unclear \| Yes / Unclear / No \| Unclear \| \| Unclear \| Yes \| Yes / Unclear / No \| Unclear \| \| Yes \| Unclear \| Yes / Unclear / No \| Unclear \| \| No \| No \| Yes / Unclear / No \| High \| \| No \| Yes / Unclear \| Yes / Unclear / No \| High \| \| Yes / Unclear \| No \| Yes / Unclear / No \| High \| \| Exception: inappropriate exclusion was considered as important (list supra). In that case the study was marked as high risk. Signaling Q1 and Q2 were considered as high impact signaling questions. \| \| \| \| \| | The following parameters were considered for applicability of the patient selection:  • Demographic features  • Differential diagnosis or co-morbidity (i.e.COPD, cardiac surgery)  • Setting of study: only assessments performed while admitted to the intensive care unit.  • Previous testing protocols (included based on phrenic nerve stimulation or maximal inspiratory pressure) |  |
| **Domain 2: Index test** | Q1: Were the index test results interpreted without knowledge of the results of the reference standard? Were the assessors blinded? This signalling question did not need further clarification. Q2: If a threshold was used, was it pre-specified? This signaling question was not considered as important as almost all papers did not pre-specify the threshold.   \| **Domain 2: Interpretation of signaling questions (Q)** \| \| \| \| --- \| --- \| --- \| \| **Q1** \| **Q2** \| **Risk of bias** \| \| Yes \| Yes / Unclear / No \| Low \| \| Unclear \| Yes / Unclear / No \| Unclear \| \| No \| Yes / Unclear / No \| High \| \| Signaling Q1 was considered as high impact signaling questions. \| \| \| | Variation in technology, execution or interpretation and methodology were evaluated for applicability.  For maximal inspiratory pressure, the only applicable method considered was the Marini method.  It needed to be clearly mentioned who performed the measurements. |  |
| **Domain 3: Reference standard** | Q1: Is the reference standard likely to correctly classify the target condition? Only reference standard who have 100% sensitive and specific were considered as low risk. Q2: Were the reference standard results interpreted without knowledge of the results of the index test? Were the assessors blinded? This signalling question did not need further clarification.   \| **Domain 3: Interpretation of signaling questions (Q)** \| \| \| \| --- \| --- \| --- \| \| **Q1** \| **Q2** \| **Risk of bias** \| \| Yes \| Yes \| Low \| \| Unclear \| Unclear \| Unclear \| \| Unclear \| Yes \| Unclear \| \| Yes \| Unclear \| Unclear \| \| No \| No \| High \| \| No \| Yes / Unclear \| High \| \| Yes / Unclear \| No \| High \| \| Signaling Q1 and Q2 were considered as high impact signaling questions. \| \| \| | In the case that the target condition was not specified it was marked as unclear |  |
| **Domain 4: Flow and Timing** | Q1: Was there an appropriate interval between index test and reference standard? Index test should be performed as close as possible to the reference standard (i.e during spontaneous breathing trial or during mechanical ventilation right before spontaneous breathing trial) without knowing the result of the reference test. Q2: Did all patients receive the same reference standard? This signalling question did not need further clarification. Q3: Were all patients included in the analysis? In case patients were measured with the index test and died before weaning outcome was determined, it is inappropriate to exclude these patients from the analysis and will result in a high risk.   \| **Domain 4: Interpretation of signaling questions (Q)** \| \| \| \| \| --- \| --- \| --- \| --- \| \| **Q1** \| **Q2** \| **Q3** \| **Risk of bias** \| \| Yes \| Yes \| Yes \| Low \| \| Unclear \| Yes \| Yes \| Low \| \| Yes \| Unclear \| Yes \| Low \| \| Yes \| Yes \| Unclear \| Low \| \| Unclear \| Unclear \| Unclear \| Unclear \| \| Yes \| Unclear \| Unclear \| Unclear \| \| Unclear \| Yes \| Unclear \| Unclear \| \| Unclear \| Unclear \| Yes \| Unclear \| \| No \| No \| No \| High \| \| No \| Yes / Unclear / No \| Yes / Unclear / No \| High \| \| Yes / Unclear / No \| No \| Yes / Unclear / No \| High \| \| Yes / Unclear / No \| Yes / Unclear / No \| No \| High \| \| Signaling Q1, Q2 and Q3 were considered as high impact signaling questions. \| \| \| \| | NA |  |

| Table S3. Characteristics table of studies investigating predictive accuracy of assessment methods to predict weaning success. | | | | | | | |
| --- | --- | --- | --- | --- | --- | --- | --- |
| **Study** | **Country** | **Setting and population** | **Sample size (n)** | **Threshold** | **Equipment used** | **Assessment protocol** | **Definition of weaning success^b^** |
| **Maximal inspiratory pressure, PImax** | | | | | | | |
| Bruton 2002^[7]^ | UK | ICU, MV patients | 19 | 17.5 cmH_2_O | - A commercially available mouth pressure meter (Precision Medical UK Ltd) attached via serial interface to a laptop computer. | - Patient position: supine position (45°).  - Timepoint of measurement: NR  - Maneuver: The equipment was inserted into the MV circuit with a 3-way tap, permitting the subject to continue with his or her current mode of assisted ventilation until the moment of measurement, the 3-way tap was turned to connect them to the sustained maximal inspiratory pressure equipment.  - Lung volume from which the effort was performed: RV  - Number of breathing efforts/tests performed: Single maximal effort performed. | Not requiring reintubation and not requiring additional respiratory support within 24h of extubation. |
| Capdevila 1995^[8]^ | France | ICU, MV patients ready to wean | 67 | 50 cmH_2_O | - A side port proximal to the endotracheal or tracheostomy tube  - Differential pressure transducer (MLR 2, Les Clayes, France).  - ultrarapid ink jet printer (Oscillomink, Siemens, Sweden) to print the signal. | - Patient position: supine position (30 ˚- 45˚).  - Timepoint of measurement: during a 20-min T-piece trial.  - Maneuver: described by Marini et al., 1986^[9]^.  - Lung volume from which the effort was performed: NR  - Number of breathing efforts/tests performed: NR | Reinstitution of MV was not necessary in the 48h following extubation. |
| Carrie 2017^[10]^ | France | ICU, MV patients | 30 | 30 cmH_2_O† | NR | - Patient position: NR  - Timepoint of measurement: NR  - Maneuver: NR  - Lung volume from which the effort was performed: NR  - Number of breathing efforts/tests performed: NR | The success of SBT and not requiring reinstitution of MV for > 48h^a^. |
| Conti 2004^[11]^ | Italy | ICU, MV patients | 50 | 16 cmH_2_O | NR | - Patient position: NR  - Timepoint of measurement: during the first 2 min after discontinuation of MV (reported in Conti et al. 1992^[12]^).  - Maneuver: NR  - Lung volume from which the effort was performed: FRC (reported in Conti et al. 1992^[12]^).  - Number of breathing efforts/tests performed: NR | Sustained SB > 48h after extubation. |
| De Jonghe 2007^[13]^* | France | MICU, SICU,  MV patients | 79 | 30 cmH_2_O | NR | - Patient position: supine position (45°).  - Timepoint of measurement: first day of return to normal consciousness.  - Maneuver: PImax was measured after a forced expiration against a manual occlusion of the respiratory circuit and held for ≥ 1s.  - Lung volume from which the effort was performed: RV  - Number of breathing efforts/tests performed: three tests were performed, selecting the highest value. | No reintubation > 48h after extubation^a^. |
| de Souza 2012^[14]^ | Brazil | ICU, MV patients, ready to wean | 103 | 30 cmH_2_O | - Unidirectional valve  - Digital vacuometer (MVD 300, Globalmed, Porto Alegre, Rio Grande do Sul, Brazil). | - Patient position: supine position (45˚).  - Timepoint of measurement: before SBT.  - Maneuver: Patients were disconnected from MV and 20s occlusion was applied using a unidirectional valve method. Pre-oxygenation was used beforehand.  - Lung volume from which the effort was performed: FRC  - Number of breathing efforts/tests performed: one period of 20s occlusion. | Sustained SB > 48h after withdrawal from MV. |
| Flevari 2016^[15]^ | Greece | ICU, MV patients with difficult and prolonged weaning | 27 | 20 cmH_2_O | NR | - Patient position: supine position.  - Timepoint of measurement: during SBT.  - Maneuver: describe by ATS/ ERS.,2002^[16]^.  - Lung volume from which the effort was performed: approaching RV.  - Number of breathing efforts/tests performed: NR | No reinstitution of MV or NIV within 48h after SBT^a^. |
| Jiang  2004^[17]^ | Taiwan | ICU, MV patients | 55 | 20 cmH_2_O | NR | - Patient position: supine position.  - Timepoint of measurement: after SBT.  - Maneuver: NR  - Lung volume from which the effort was performed: NR  - Number of breathing efforts/tests performed: NR | Patients who did not receive reintubation or NIPPV within 72h after extubation^a^. |
| Lim  2015^[18]^ | Taiwan | ICU and respiratory care center, MV patients | 86 | 33 cmH_2_O | - Unidirectional valve  - Manometer (Boehringer Laboratories, Norristown, PA, USA) | - Patient position: supine position (30° - 45°).  - Timepoint of measurement: after conversion of the endotracheal tube to a tracheostomy.  - Maneuver: described by Truwit, Marini,1992^[19]^.  - Lung volume from which the effort was performed: NR  - Number of breathing efforts/tests performed: three tests were performed, selecting the highest value. | Liberation from MV support for at least 5 consecutive days. |
| Medrinal 2016^[20]^* | France | ICU, MV patients | 124 | 30 cmH_2_O | - Unidirectional valve  - Electronic manometer, micro-RPM (Eolys, PAYS) | - Patient position: supine position (45°).  - Timepoint of measurement: after SBT.  - Maneuver: describe by ATS/ ERS.,2002^[16]^.  - Lung volume from which the effort was performed: RV  - Number of breathing efforts/tests performed: three tests were performed, selecting the highest value. | NR |
| O'Keefe 2001^[21]^ | USA | SICU and trauma ICU, MV patients | 95 | 30 cmH_2_O | - Unidirectional valve  - Pressure transducer | - Patient position: NR  - Timepoint of measurement: daily measured at 5am until patients was successfully liberated from MV.  - Maneuver: described by Marini et al., 1986^[9]^.  - Lung volume from which the effort was performed: NR  - Number of breathing efforts/tests performed: NR | 24h of unassisted ventilation. |
| Saeed 2016^[22]^ | Egypt | RICU, MV COPD patients | 30 | 20 cmH_2_O | NR | - Patient position: NR  - Timepoint of measurement: during SBT.  - Maneuver: NR  - Lung volume from which the effort was performed: NR  - Number of breathing efforts/tests performed: NR | No reventilation for > 48h after extubation^a^. |
| Sassoon 1993^[23]^ | USA | ICU, MV patients, recovering from ARF of various aetiologies, ready to wean | 45 | 20 cmH_2_O | - A side port proximal to the endotracheal tube  - Differential pressure transducer (MP45 ± 100 cm H20 ; Validyne Corp., Northridge, CA). | - Patient position: supine position (30 ˚- 45˚).  - Timepoint of measurement: after 5min of 1h 5 cmH_2_O CPAP trial.  - Maneuver: described by Marini et al., 1986^[9]^.  - Lung volume from which the effort was performed: NR  - Number of breathing efforts/tests performed: three tests were performed, selecting the highest value. | Ability to complete the 1-h trial or completion of the trial without siqns of cardiorespiratory distress within 48h of discontinuation from MV^a^. |
| Shamil 2022[24] | India | ICU, MV patients ready to wean | 50 | 30 cmH2O† | NR | - Patient position: supine position (30°-45°).  - Timepoint of measurement: during SBT  - Maneuver: maximum inspiratory pressure was noted from the ventilator display.  - Lung volume from which the effort was performed: NR  - Number of breathing efforts/tests performed: NR | No reintubation or need of noninvasive ventilation (NIV) for the patient within 48 hours after extubation. |
| Spadaro 2016^[25]^ | Italy | ICU, MV (intubated) patients | 51 | 30 cmH_2_O† | - A portable manometer (MicroRPM™; CareFusion Corporation, San Diego, USA). | - Patient position: supine position (30˚-45˚)  - Timepoint of measurement: during SBT.  - Maneuver: described by Marini et al.,1986^[9]^.  - Lung volume from which the effort was performed: FRC.  - Number of breathing efforts/tests performed: three tests of PImax were performed. | Patients were extubated and SB > 48h. |
| Tenza-Lozano 2018^[26]^ | Spain | ICU, MV patients | 62 | 30 cmH_2_O† | NR | - Patient position: NR  - Timepoint of measurement: NR  - Maneuver: NR  - Lung volume from which the effort was performed: NR  - Number of breathing efforts/tests performed: NR | Patients were extubated and SB > 48h. |
| Vieira 2022^[27]^ | Brazil | ICU, MV and tracheostomized patients | 70 | 30 cmH_2_O† | - Digital manovacuometer (MVD 500, Globalmed®) | - Patient position: supine (30°).  - Timepoint of measurement: during spontaneous ventilation within 48h after tracheostomy.  - Maneuver: the digital manovacuometer was connected to the tracheostomy followed by an inspiratory occlusion for 20-25s.  - Lung volume from which the effort was performed: RV  - Number of breathing efforts/tests performed: the highest values of three reproducible measures was recorded. | 48h free of MV for patients who underwent MV for up to 20 days; 5 consecutive days without MV for patients who underwent MV for a period of 21 days or more. |
| Zaytoun 2021^[28]^ | Egypt | ICU, MV patients | 64 | 30 cmH_2_O | NR | - Patient position: NR  - Timepoint of measurement: NR  - Maneuver: measured by asking the patient to exhale as deeply as possible. During expiration, the measurements were initiated by pressing on the expiratory pause button, and the patient was asked to inspire as fast as possible and as powerful as possible.  - Lung volume from which the effort was performed: RV  - Number of breathing efforts/tests performed: the maximum reading from three breaths was recorded. | Patient was extubated and maintained his/her SB >48 h without any ventilatory support. |
| **Diaphragmatic excursion, DE** | | | | | | | |
| **Study** | **Country** | **Setting and population** | **Sample size (n)** | **Threshold** | **Equipment used** | **Assessment protocol** | **Definition of weaning success^b^** |
| Abbas 2018^[29]^ | Egypt | RICU, patients with AECOPD at their first SBT | 50 | 12 mm | - SonoScape ultrasound machine (SonoScape SSI-4000, SonoScape Medical Corp., Guangdong, China; EC REP SonoScape Europ S.R.L, Rome, Italy) | - Patient position: supine position (30 °- 45 °).  - Timepoint of measurement: after 30 min from the beginning of the SBT.  - Maneuver: the probe was placed over one of the lower intercostal spaces in the right anterior axillary line; the ultrasound beam was directed to the hemidiaphragm dome.  - Mode of US: M-mode.  - Type of probe: 3.5-MHz.  - Number of measurements: average of six measurements. | Extubated and SB ≥ 48 h^a^. |
| Abdelhafeez 2019^[30]^ | Egypt | RICU, MV patients | 240 | >18.4 mm | - Mindray DP-2200; Shenzen Lontek Electronic Technology Co., Limited, China | - Patient position: supine position.  - Timepoint of measurement: just before extubation.  - Maneuver: the probe was placed between the midclavicular and anterior axillary lines, in the subcostal area, and directed medially, cranially, or dorsally, so that the ultrasound beam reached perpendicularly the posterior third of the right hemi-diaphragm.  - Mode of US: M-mode.  - Type of probe: 3.5-5 MHz curvilinear probe.  - Number of measurements: measurements of at least three respiratory cycles were averaged for the right and left hrmidiaphragm. Then the average of measurements of the two sides was calculated. | **Weaning success**: simple weaning = weaning duration less than week and patient weaned successfully from the first weaning trial; difficult weaning = weaning duration up to a week and patient weaned successfully after three weaning trials. **Weaning failure** was defined as one of the following: nonscheduled extubation, need for reintubation, need for noninvasive positive pressure ventilation of the patient within 48h after extubation, delayed extubation, tracheostomy. |
| Alam  2022^[31]^ | Bangladesh | ICU, MV patients | 31 | 11.43 mm | -Sonosite M-Turbo (FUJIFILM SonoSite, Bothell, WA, USA) | - Patient position: supine position (20 °- 40 °).  - Timepoint of measurement: at the start and after 10 min of a T-piece trial, and just before extubation  - Maneuver: the probe was placed directly under the right costal edge along the mid-clavicular line and directed medially, cephalad, and dorsally such that the ultrasonic beam reaches the posterior part of the diaphragm perpendicularly.  - Mode of US: M-mode.  - Type of probe: 2-5 MHz curvilinear probe.  - Number of measurements: average of three measurements. | Continuation of SB ≥ 48h after extubation |
| Ali  2017^[5]^ | Egypt | ICU, MV patients | 60 | 15 mm | - Echo Blaster 128 Kit | - Patient position: supine position (0 °-20 °).  - Timepoint of measurement: NR  - Maneuver: measurements during tidal breathing, excluding smaller or deeper breaths. The probe was placed anterior subcostal between midclavicular and axillary lines.  - Mode of US: M-mode.  - Type of probe: 4 MHz curvilinear transducer.  - Number of measurements: average of six measurements. | Ability to maintain SB ≥ 48h without any level of MV support. |
| Al Tayar 2022^[32]^ | Saudi Arabia | ICU, MV patients | 24 | 15.6 mm | - CX50 (Philips, Amsterdam, the Netherlands) | - Patient position: NR  - Timepoint of measurement: after 30 min of SBT.  - Maneuver: NR  - Mode of US: NR  - Type of probe: curvilinear probe 2-5 MHz.  - Number of measurements: average of three measurements. | No need for re-intubation, noninvasive ventilation, or death in the first 48 h after extubation^a^ |
| Amara 2022^[33]^ | India | ICU, MV patients ready to wean | 81 | 17.9 mm | - Philips CX 50 (Philips Healthcare, 3000 Minuteman Road, Andover, USA) | - Patient position: NR  - Timepoint of measurement: on the day of the first SBT  - Maneuver: the probe was placed in subcostal space in a horizontal fashion.  - Mode of US: M-mode.  - Type of probe: curvilinear probe.  - Number of measurements: NR | Initiation of weaning to successful extubation after first SBT |
| Baess 2016^[34]^ | Egypt | ICU and RICU, MV (intubated) patients | 30 | 10 mm | - Philips Healthcare (Andover, Massachusetts, USA) | - Patient position: supine position (45°).  - Timepoint of measurement: during SBT  - Maneuver: the probe was placed immediately below the costal margin at the midclavicular line or on the last two spaces at the anterior axillary line. The probe was directed backward, caudally, and slightly medially until the dome was visualized.  - Mode of US: M-mode.  - Type of probe: 2–4 MHz phased-array probe.  - Number of measurements: average of five measurements. | SB > 48h following extubation. |
| Banerjee 2018^[35]^ | India | ICU, MV patients | 53 | 11 mm | - Siemens Acuson X300 | - Patient position: NR  - Timepoint of measurement: after 20 mins of SBT.  - Maneuver: the probe was placed subcostal parallel to the intercostal space to measure the range of the diaphragmatic movement.  - Mode of US: M-mode.  - Type of probe: curvilinear probe.  - Number of measurements: NR | Stay in SB ≥ 48 h after the extubation. |
| Carrie 2017^[10]^ | France | ICU, MV patients | 67 | 27 mm | - Vivid S5^TM^ ultrasound machine (GE Health care, Wauwatosa, WI, USA). | - Patient position: supine position (45°).  - Timepoint of measurement: before SBT.  - Maneuver: measurements during a forced expiratory maneuver after a maximal inspiration. The probe was placed between the mid-clavicular and the anterior axillary lines.  - Mode of US: M-mode.  - Type of probe: 4 MHz cardiac probe.  - Number of measurements: best of three measurements. | The success of SBT and not requiring reinstitution of MV > 48 h^a^. |
| Eksombatchai 2023^[36]^ | Thailand | MICU and SICU, ready to wean MV patients | 130 | 10.5 mm | - Sonosite M-Turbo (SonoSite Inc., Bothell, WA, USA) | - Patient position: supine position (30°-45°)  - Timepoint of measurement: at 2h after the SBT.  - Maneuver: probe was placed at the junction of the mid-clavicular line and subcostal margin or intercostal space in which the ultrasound beam paralleled the direction of diaphragmatic movement.  - Mode of US: M-mode  - Type of probe: 1-to-5 MHz probe  - Number of measurements: average of three consecutive tidal breaths. | No reintubation reintubation within 48h after extubation^a^. |
| Elgazzar 2019^[37]^ | Egypt | RICU, MV patients | 30 | 9.1 mm | - Doppler ultrasound system, model S6 (SonoScape, Shanghai, China) | - Patient position: semi-sitting position.  - Timepoint of measurement: during SBT.  - Maneuver: the vertical space between upper border of the liver at end of expiration to that at the end of inspiration was measured.  - Mode of US: M-mode.  - Type of probe: NR  - Number of measurements: NR | SB ≥ 48 h, without any support from the MV and without developing failure measures of SB. |
| Elshazly 2020^[38]^ | Egypt | RICU, MV patients | 62 | 12.5 mm | - Transthoracic ultrasound (Logiq P3, GE Healthcare, Chicago, IL, USA) | - Patient position: semi-sitting position.  - Timepoint of measurement: during SBT.  - Maneuver: measurements during tidal breathing. The probe was placed between the mid-clavicular and anterior axillary lines.  - Mode of US: M-mode.  - Type of probe: 3.5 MHz curvilinear probe.  - Number of measurements: NR | SB duration after extubation > 48h. |
| Eltrabili 2019^[39]^ | Egypt | SICU, MV patients | 30 | 10.4 mm | - Mindray model M7  ultrasound machine (Mindray Bio-Medical, Shenzhen, China) | - Patient position: supine position.  - Timepoint of measurement: after SBT.  - Maneuver: probe placed over one of the lower intercostal spaces in the right anterior axillary line, during inspiration, the normal diaphragm contracts and moves caudally toward the transducer.  - Mode of US: M-mode.  - Type of probe: 3–5 MHz curvilinear probes.  - Number of measurements: NR | Patient stays in SB ≥ 48h after extubation. |
| Er 2021^[40]^ | Turkey | MICU, MV patients | 38 | 22.1 mm | - Acuson X700, Siemens | - Patient position: supine position.  - Timepoint of measurement: during the first SBT.  - Maneuver: measurements were done during breaths with a tidal volume of 6-8ml/kg of ideal body weight. The probe was positioned in the anterior subcostal area in the mid-clavicular line and directed cranially and dorsally by using the liver window.  - Mode of US: M-mode.  - Type of probe: 3.1 MHz phase array probe.  - Number of measurements: average value of three consecutive measurements. | No reintubation or death within 7 days after extubation |
| Farghaly 2017^[3]^ | Egypt | RICU, MV patients | 54 | 10.5 mm | - Samsung Medison Sono Ace R3 ultrasound system (Samsung company, Seoul, South Korea) | - Patient position: supine position (45°)  - Timepoint of measurement: during SBT.  - Maneuver: measurements during tidal breathing. The probe was placed below the right subcostal margin in the mid-clavicular line.  - Mode of US: M-mode.  - Type of probe: 3.5 MHz probe.  - Number of measurements: average of three measurements. | Maintenance of SB > 48h following extubation. |
| Flevari 2016^[15]^ | Greece | ICU, MV patients with difficult and prolong weaning | 27 | 10 mm | NR | - Patient position: supine position (0-10°).  - Timepoint of measurement: NR  - Maneuver: measurements during quiet breathing. The probe was placed in the subcostal area in the midclavicular line.  - Mode of US: M-mode.  - Type of probe: 5 MHz.  - Number of measurements: average of five consecutive measurements. | No reinstitution of MV or NIV within 48h after SBT^a^. |
| Fossat 2022^[41]^ | France | MICU, MV patients | 99 | 12 mm† | - GE HealthCare | - Patient position: NR  - Timepoint of measurement: after 25 min of SBT.  - Maneuver: described by Zambon et al. 2017[42]).  - Mode of US: M-mode.  - Type of probe: 5 MHz cardiac probe.  - Number of measurements: NR | No need for re-intubation or for the initiation of curative noninvasive ventilation (NIV) before or at day 7 after extubation |
| Gok 2021^[43]^ | Turkey | ICU, MV patients | 46 | 13.1 mm | - Philips Affiniti (Philips, Amsterdam, Netherlands | - Patient position: NR  - Timepoint of measurement: during SBT (T-piece trial).  - Maneuver: NR  - Mode of US: M-mode.  - Type of probe: 2-4 MHz curvilinear probe.  - Number of measurements: average of five measurements. | No re-intubation or need for NIV within 48h after extubation^a^. |
| Haji  2018^[44]^ | Australia | MICU and SICU, MV patients | 53 | 10 mm | -Vivid E9 (GE Health-care, 9900 Innovation Drive, Wauwatosa, WI 53226, USA) | - Patient position: supine position (30° – 50°).  - Timepoint of measurement: beginning of SBT.  - Maneuver: probe was placed laterally and perpendicularly on the lower intercostal spaces of the lateral chest wall between mid- and posterior axillary line.  - Mode of US: NR  - Type of probe: 1.8–6.0 MHz curvilinear probe.  - Number of measurements: average of three measurements. | No re-intubation, NIV support or death within 48h after extubation^a^. |
| Hayat 2017^[45]^ | Pakistan | MICU, MV patients | 100 | 12 mm | NR | - Patient position: supine position.  - Timepoint of measurement: after extubation.  - Maneuver: measurements during tidal breathing. The probe was placed at right anterior axillary lines.  - Mode of US: NR  - Type of probe: 3.5 MHz curvilinear probe.  - Number of measurements: average of three measurements. | SB without MV ≥ 48h. |
| Helmy 2021^[46]^ | Egypt | ICU, MV patients | 22 | 11 mm | - Samsung HS60 ultrasound machine | - Patient position: supine position (30 °- 45 °).  - Timepoint of measurement: 15 min after initiation of SBT (PS = 5 cmH2O, PEEP = 5 cmH2O).  - Maneuver: the probe was placed over one of the lower intercostal spaces in the right anterior axillary line for the right diaphragm. The ultrasound beam was directed to the hemi-diaphragmatic domes at an angle ≥ 70°.  - Mode of US: M-mode.  - Type of probe: 3-5 MHz curvilinear probe.  - Number of measurements: average of three measurements. | Patients did not require any ventilatory support 72h after extubation. |
| Hirolli 2023^[47]^ | India | NICU, MV patients | 43 | 12 mm | - M turbo, Sonosite, Bothell, WA | - Patient position: supine position (20 - 40°)  - Timepoint of measurement: during SBT, before extubation after the patient was deemed fit for extubation.  - Maneuver: NR  - Mode of US: NR  - Type of probe: 2–6-MHz curvilinear probe.  - Number of measurements: average of three measurements. | No reintubation within 48h of extubation^a^. |
| Huang 2017^[48]^ | China | MICU, MV patients | 40 | 10.7 mm | - EPIQ5 machine (Philips, Holland) | - Patient position: supine position (45°)  - Timepoint of measurement: during SBT.  - Maneuver: the probe was placed on the chest wall; the ultrasound beam was directed to the hemi-diaphragmatic dome. Diaphragm contraction during inspiration was observed as an upward motion.  - Mode of US: M-mode.  - Type of probe: 1-5 MHz curvilinear probe.  - Number of measurements: average of six measurements. | Patient could maintain SB ≥ 48h with no need for any level of MV support after extubation. |
| Huang 2023^[49]^ | China | ICU, MV patients | 88 | 9.95 mm | - TE7 Diagnostic Ultrasound System (Shenzen Mindray Biomedical, China) | - Patient position: supine position (30°-45°)  - Timepoint of measurement: before SBT.  - Maneuver: all patients had spontaneous breathing with the PS of 10-12 cmH2O. The ultrasound beam was prerpendicular to the posterior diaphragm when the manual measurement was taken. With the automatic measurement, three to six region of interest on the diaphragm are automatically chosen; the displacement of those regions of interest is calculated and subsequently averaged.  - Mode of US: M-mode  - Type of probe: 2-5 MHz probe  - Number of measurements: NR | Patient could maintain SB ≥ 48h with no need of any level of MV after extubation |
| Jiang  2004^[17]^ | Taiwan | ICU, MV patients | 55 | 11 mm | - Aloka Echo Camera SSD-1400 (Aloka; Zug, Switzerland) | - Patient position: supine position.  - Timepoint of measurement: at the beginning of T-piece trial.  - Maneuver: measurements during quiet breathing, excluding deep breathing. The probe was placed along the right anterior axillary line.  - Mode of US: NR  - Type of probe: 3.5-MHz probe.  - Number of measurements: average of ten measurements per operator, for a total of two operators. The mean of the two averages was recorded. | Patients who did not receive reintubation or NIPPV within 72h after extubation^a^. |
| Kaur 2022^[50]^ | India | ICU, MV patients ready to wean | 50 | 11 mm | - FUJIFILM ((SonoSite Inc., Bothell, WA, USA) | - Patient position: supine position.  - Timepoint of measurement: during SBT.  - Maneuver: The probe was placed subcostally parallel to  the intercostal space between the mid-clavicular  and the mid-axillary lines with the beam directed  medially, cephalad, and dorsally.  - Mode of US: M-mode  - Type of probe: 3-5 MHz curvilinear probe.  - Number of measurements: average of three values. | Ability to sustain SB after removal of the endotracheal tube or tracheostomy tube > 48 hours, without the need for re-intubation^a^. |
| Khan 2018^[51]^ | Pakistan | MICU, MV patients | 90 | 13.5 mm | NR | - Patient position: NR  - Timepoint of measurement: during SBT.  - Maneuver: the probe was placed at the right hypochondrial area.  - Mode of US: M-mode.  - Type of probe: 3.5 to 5 MHz curvilinear probe.  - Number of measurements: NR | No MV or NIV within 48h after extubation. |
| Kim  2011^[52]^ | Republic of Korea | MICU, MV patients | 82 | 10 mm | - Esaote ultrasound machine (Esaote My Lab 30CV, Genoa, Italy) | - Patient position: supine position.  - Timepoint of measurement: during SBT.  - Maneuver: the probe was placed over one of the lower intercostal spaces in the right anterior axillary.  - Mode of US: M-mode.  - Type of probe: 3.5-MHz probe.  - Number of measurements: average of six measurements.. | Patient was able to maintain SB ≥ 48h without any level of MV support^a^. |
| Li 2021^[53]^ | China | ICU, elderly patients (<= 65years) | 101 | 13 mm | Mindray probe | - Patient position: NR  - Timepoint of measurement: during SBT  - Maneuver: probe placed over one of the lower intercostal spaces in the right anterior axillary line for the right diaphragm and the liver, serving as an acoustic window. Two-dimensional (2D) mode was used to search for the line of the right hemidiaphragm.  - Mode of US: M-mode  - Type of probe: 2-5 MHz probe  - Number of measurements: NR | Extubation success was defined as sustained SB > 48h following extubation without noninvasive positive-pressure ventilation (NPPV) or invasive ventilation. |
| Luo  2017^[6]^ | China | RICU, SICU, CCU and EICU, difficult to wean patients | 46 | 12.6 mm | - Vivid i (GE Medical Systems Israel Ltd) | - Patient position: supine position (30°- 45°).  - Timepoint of measurement: after SBT.  - Maneuver: the probe was placed over one of the lower intercostal spaces in the right anterior axillary line.  - Mode of US: M-mode.  - Type of probe: 3.0 MHz probe.  - Number of measurements: average of six measurements. | No Reintubation within 1 week. |
| Mariani 2016^[54]^* | France | ICU,  MV patients | 34 | 11 mm | NR | - Patient position: supine position (30°).  - Timepoint of measurement: during SBT.  - Maneuver: measurements during tidal breathing. The probe was placed between the 8^th^ and 9^th^ ribs on midaxillary lines. The ultrasound beam was directed perpendicularly to the diaphragmatic line during inspiration.  - Mode of US: M-mode.  - Type of probe: 4-2 MHz probe.  - Number of measurements: best value of three or four measurements. | No Reintubation within 72h to 96h after extubation. |
| Mawla 2022^[55]^ | Egypt | ICU, MV patients | 90 | 13 mm | Philips HD11XE | - Patient position: semi-recumbent  - Timepoint of measurement: during SBT  - Maneuver: subcostally parallel to the intercostal space between mid clavicular and anterior axillary lines, directed medially, cranially and dorsally in order to demonstration of the posterior third of the right diaphragm.  - Mode of US: M-mode  - Type of probe: 4MHz  - Number of measurements: mean of 5 measurements | Able to maintain their own breathing for 48h after extubation without any level of ventilator support |
| Mohamed 2021^[56]^ | Egypt | ICU, MV patients | 80 | 17 mm | NR | - Patient position: head of the bed elevated between 20 and 40°  - Timepoint of measurement: after SBT success  - Maneuver: probe is placed in the right subcostal region parallel to the intercostal space to measure the range of the diaphragmatic movement  - Mode of US: M-mode  - Type of probe: convex probe  - Number of measurements: mean of at least 3 measurements | Successful extubation was defined as maintenance of SB > 48h following extubation |
| Osman 2017^[57]^ | Egypt | ICU, MV patients | 68 | 10 mm | - Logic E9 (GE) and Honda electronics HS-2100 portable ultrasound machine. | - Patient position: supine position (45°).  - Timepoint of measurement: during SBT.  - Maneuver: the probe was placed subcostally parallel to the intercostal space.  - Mode of US: M-mode.  - Type of probe: 3.5 MHz curvilinear probe.  - Number of measurements: NR | No reintubation > 48h after weaning trial^a^. |
| Palkar 2018^[58]^ | USA | MICU, MV patients | 73 | 10 mm | - M turbo P21 probe (SonoSite, Bothell, WA) | - Patient position: supine position (20°- 40°).  - Timepoint of measurement: after 30 mins of SBT.  - Maneuver: measurements during tidal breathing. The probe was placed immediately below the right costal margin in the mid-clavicular line.  - Mode of US: M-mode.  - Type of probe: 3.5 MHz curvilinear probe.  - Number of measurements: average of three measurements. | Ability to maintain SB ≥ 48h after extubation. |
| Saad 2022^[59]^ | Egypt | ICU, MV patients | 64 | 61 mm | PHILIPS HD5 release 2.1 (distributed by PHILIPS healthcare, Bothell, WA, USA) @2011Koninklijke Philips N.V. | - Patient position: supine position.  - Timepoint of measurement: t-piece during SBT, measured 30 min after disconnecting mechanical ventilation  - Maneuver: during deep breathing, the probe was placed perpendicular to the chest wall or with angle not less than 70° according difficulty of the case, in the eighth or ninth intercostal space, between the anterior axillary and the midaxillary lines. The liver was identified as a window for right hemidiaphragm and the spleen was identified as a window for left hemidiaphragm. The ultrasound probe was placed in the direction in which the ultrasound beam reached the posterior third of the corresponding hemidiaphragm perpendicularly.  - Mode of US: M-mode  - Type of probe: 1 - to 5-MHz ultrasound curved probe  - Number of measurements: three consecutive maximal breaths on the right side and three on the left side, and the average values on each side were used for analysis | Failure of weaning was considered when patient needed MV during SBT and patient was reintubated and ventilated or needed non-invasive ventilation (NIV) within 48h |
| Saeed 2016^[22]^ | Egypt | RICU, MV patients with COPD | 30 | 14 mm | - Mindray DP 1100 ultrasound machine | - Patient position: supine position.  - Timepoint of measurement: after SBT.  - Maneuver: the probe was placed on anterior subcostal abdominal wall at the midclavicular line. A fixed point was taken on the outer surface (point A) and the distance from this point to the diaphragm (point B) was measured during inspiration and expiration.  - Mode of US: B-mode.  - Type of probe: 2–5 MHz curvilinear phased array probe  - Number of measurements: average of three to five measurements. | No re-ventilation ≥ 48h after extubation^a^. |
| Saeed 2019^[60]^ | Egypt | RICU, MV patients with COPD | 32 | 16 mm | Mindray M7 (Guangzhou Medsinglong Medical Equipment Co. Ltd, Guangdong, China) | - Patient position: supine position.  - Timepoint of measurement: during PS mode.  - Maneuver: the transducer was put on anterior subcostal abdominal wall at the midclavicular line operating a transverse scanning with cranial direction to display the best imaging of the hemi-diaphragmatic dome.  - Mode of US: B-mode.  - Type of probe: 3.5C (bandwidth 2–5MHz) convex phased array probe.  - Number of measurements: average of three to five measurements. | Extubation and the absence of ventilator support 48h following the extubation. |
| Samanta 2017^[61]^ | India | MICU, SICU, MV patients ready to wean | 64 | 12 mm | - Fujifilm SonoSite, Inc. | - Patient position: supine position (45°).  - Timepoint of measurement: during the first T-piece trial.  - Maneuver: the probe was placed on the right subcostal margin between the mid-clavicular and anterior axillary line.  - Mode of US: M-mode.  - Type of probe: 3.5-5 MHz curvilinear.  - Number of measurements: average of three measurements. | Patients who proceeded from initiation of weaning to extubation success on their first SBT without any difficulty were categorized as simple weaning. |
| Saravanan 2022^[62]^ | India | ICU, MV patients | 200 | 12.1 mm | - GE LogiQ machine, China | - Patient position: supine position (45°)  - Timepoint of measurement: before extubation.  - Maneuver: the probe was placed in the right subcostal margin and tilted cranially to visualize the dome of the diaphragm. Diaphragm excursion was measured during deep breathing.  - Mode of US: M-mode  - Type of probe: 3-5 MHz curvilinear probe.  - Number of measurements: NR | Ability of the patient to tolerate spontaneous breathing without the use of non-invasive ventilation for at least 48h after extubation. |
| Shamil 2022^[24]^ | India | ICU, MV patients ready to wean | 50 | 12 mm† | NR | - Patient position: position: supine position (30°-45°).  - Timepoint of measurement: during SBT.  - Maneuver: measurements performed during tidal breathing while patients ventilated with PSV (PEEP: 5 cmH2O, PS: 5 cmH2O); the probe was placed on the right side of the chest at  the anterior subcostal location between the midclavicular line and anterior axillary line. The ultrasound beam would reach the posterior part of the diaphragm at an angle of not less than 70°.  - Mode of US: B- and M-mode  - Type of probe: 3.5 MHz curvilinear probe.  - Number of measurements: average of five measurements. | No reintubation or need of noninvasive ventilation (NIV) for the patient within 48 hours after extubation. |
| Song 2022^[63]^ | China | ICU, MV patients | 110 | 13.5 mm | M-turbo; (FUJIFILM Sonosite, Washington, USA) | - Patient position: supine (30°-45°)  - Timepoint of measurement: after 30 min from the beginning of the SBT or immediately before returning to the initial ventilator settings in case of SBT failure.  - Maneuver: the probe was placed immediately below the right costal margin on the anterior axillary or midclavicular line in the longitudinal scanning plane, with the tomographic plane angled in the cephalad direction such that the ultrasound beam was perpendicular to the posterior third of the right hemidiaphragm.  - Mode of US: M-mode  - Type of probe: 3.5 MHz curvilinear probe  - Number of measurements: average of three measurements. | SBT success and ability to maintain SB > 48h without needing non-invasive or invasive ventilation. |
| Spadaro 2016^[25]^ | Italy | ICU, MV patients | 51 | 14 mm | NR | - Patient position : supine position (30 - 45˚).  - Timepoint of measurement: after 30 min of SBT.  - Maneuver: described by Kim WY et al., 2011^[52]^.  - Mode of US: M-mode.  - Type of probe: 3.5 MHz curvilinear probe.  - Number of measurements: average of three measurements. | Patients were extubated and SB > 48h. |
| Spadaro 2021^[64]^ | Italy | ICU, MV patients | 62 | 10 mm | M-Turbo, SonoSite, Inc., USA | - Patient position: supine position (30 - 45°).  - Timepoint of measurement: day 3 after start of weaning.  - Maneuver: right subcostal approach as described in Spadaro et al. 2019, Goligher et al. 2015, Umbrello et al. 2015.  - Mode of US: M-mode.  - Type of probe: 3.5-5MHz.  - Number of measurements: NR | Reintubation^a^. |
| Thabet 2020^[65]^ | Egypt | RICU, MV patients | 40 | 14.1 mm | - Medesion SonoAce R3 portable ultrasound  (2010; Samsung Company, Seoul, Korea) | - Patient position: semi-sitting position.  - Timepoint of measurement: immediately after the start of SBT.  - Maneuver: the probe placed subcostally directed with angle more than 70° for visualization of the posterior third of the diaphragm.  - Mode of US: M-mode.  - Type of probe: 5 MHz curvilinear probe.  - Number of measurements: average of three measurements. | Patients who did not require any re-use of MV, either invasive or noninvasive, > 48h. |
| Theerawit 2018^[66]^ | Thailand | MICU and SICU, MV patients ready to wean | 62 | 12.85 mm | - SonoSite M-Turbo (SonoSite Inc., Bothell, WA, USA) | - Patient position: supine position.  - Timepoint of measurement: at the end of a 2h SBT.  - Maneuver: measurements during tidal breathing. The probe was placed in the direction in which the ultrasound beam reached the posterior third of the hemidiaphragm.  - Mode of US: M-mode.  - Type of probe: 1 to 5 MHz.  - Number of measurements: average of three measurements. | Ability to tolerate SB ≥ 48h without any level of assisted ventilation^a^. |
| Varón-Vega 2021^[1]^ | Colombia | ICU, MV patients | 84 | 10 mm | Sonocare ultrasound system (Sonosite EDGE 03VRYF) | - Patient position: supine position (45°).  - Timepoint of measurement: after 30min of SBT.  - Maneuver: the transducer was positioned just below the ribcage, between the clavicular midline and the anterior axillary line. The ultrasound beam was directed cephalad, perpendicular to the posterior third of the diaphragm.  - Mode of US: M-mode.  - Type of probe: 1-5 MHz.  - Number of measurements: NR | Successful extubation was defined as the capacity to maintain SB > 48h without ventilatory assistance after extubation. |
| Vieira 2022^[27]^ | Brazil | ICU, MV and tracheostomized patients | 81 | 12.5 mm | - Sonosite® instrument (2013 SonoSite M-Turbo Model M-MSK) | - Patient position: supine (30°)  - Timepoint of measurement: during spontaneous ventilation within 48h after tracheostomy.  - Maneuver: The probe was placed in the hepatic anatomical window between the middle clavicular line and the anterior axillary line, pointing medially, cranially, and dorsally, then projecting the ultrasound beam perpendicularly across the posterior third of the diaphragm.  - Mode of US: M-mode  - Type of probe: 2-5 MHz curvilinear probe  - Number of measurements: NR | 48h free of MV for patients who underwent MV for up to 20 days; 5 consecutive days without MV for patients who underwent MV for a period of 21 days or more. |
| Vivier 2019^[67]^* | France | ICU, Ready to wean patients at risk of extubation failure | 189 | 10 mm | - Vivid S6 (General Electric Healthcare, Little Chalfont, Buckinghamshire, England) or  - CX-50 (Philips Ultrasound, Bothell, WA, USA) | - Patient position: supine position (45°).  - Timepoint of measurement: during SBT.  - Maneuver: measurements during tidal breathing. The probe was placed under the subcostal area targeting the beam towards the highest point of the diaphragmatic dome.  - Mode of US: M-mode.  - Type of probe: 4-MHz linear probe.  - Number of measurements: average of three consecutive measurements. | No occurrence of death or need of re-intubation for > 7 days after planned extubation^a^ |
| Xu 2022^[68]^ | China | ICU, MV patients | 96 | 11.2 mm | - Vivid7 and Vivid6 (GE, USA) | - Patient position: supine position (45°)  - Timepoint of measurement: during the first SBT.  - Maneuver: The probe was placed at the intersection of the right midclavicular and subcostal lines, with the transducer facing the skull and dorsal side.  - Mode of US: M-mode  - Type of probe: 4-13 MHz linear probe (Vivid6) and 4-11 MHz linear probe (Vivid7).  - Number of measurements: NR | No reintubation within 48 h or no death within 7 days after extubation. |
| Yoo  2018^[69]^ | Republic of Korea | MICU or SICU, MV patients ready to wean | 60 | 14 mm | - M-Turbo, Fujifilm  SonoSite Inc., Bothell, WA, USA | - Patient position: NR  - Timepoint of measurement: during SBT.  - Maneuver: the probe was placed over one of the lower intercostal spaces. DE was measured on the vertical axis tracing from the baseline to the point of maximum height of inspiration.  - Mode of US: M-mode.  - Type of probe: 2 to 5 MHz.  -Number of measurements: NR | Sustained SB > 48h following extubation without NIV. |
| Zaytoun 2021^[28]^ | Egypt | ICU, MV patients | 64 | 15 mm | Portable ultrasound (Mindray-Model DP-20, 2015, SHENZHEN, China) | - Patient position: supine position (45°).  - Timepoint of measurement: after 30min of SBT (just before reinstitution of MV in case of SBT failure).  - Maneuver: right hemidiaphragm was evaluated, by placing the probe between the mid-clavicular line and anterior axillary line, in the subcostal region, and directed medially, posteriorly, and cranially, so that the ultrasonic waves reached perpendicularly the posterior third of the right copula of diaphragm.  - Mode of US: M-mode.  - Type of probe: 2.5-5MHz curvilinear probe.  - Number of measurements: at least three respiratory cycles were  recorded, and measurements were averaged from at  least three different cycles. | The weaning attempt was considered as successful when the patient was extubated and maintained his/her SB without any ventilatory support > 48h. |
| Zhang 2020^[70]^ | China | ICU, MV COPD patients | 37 | 17.2 mm | NR | - Patient position: supine position (30˚).  - Timepoint of measurement: during SBT.  - Maneuver: measurements during tidal breathing. The probe was placed to the subcostal area between the anterior axillary and the mid-clavicular lines.  - Mode of US: M-mode.  - Type of probe: 2–5 MHz curvilinear probe.  - Number of measurements: average of three measurements. | Ability to maintain SB ≥ 48h without any MV support. |
| **Diaphragmatic thickness fraction, DTF** | | | | | | | |
| **Study** | **Country** | **Setting and population** | **Sample size (n)** | **Threshold** | **Equipment used** | **Assessment protocol** | **Definition of weaning success^b^** |
| Abdelhafeez 2019^[30]^ | Egypt | RICU, MV patients | 240 | 30.1 % | - Mindray DP-2200; Shenzen Lontek Electronic Technology Co., Limited, China | - Patient position: supine position.  - Timepoint of measurement: just before extubation.  - Maneuver: The probe was placed in a low intercostal position between the anterior and midaxillary lines to obtain the best imaging of the left hemi-diaphragm.  - Mode of US: NR  - Type of probe: NR  - Number of measurements: Measurements of at least three respiratory cycles were averaged for the right and left hemidiaphragm. Then the average of measurements of the two sides was calculated. | **Weaning success**: simple weaning = weaning duration less than week and patient weaned successfully from the first weaning trial; difficult weaning = weaning duration up to a week and patient weaned successfully after three weaning trials. **Weaning failure** was defined as one of the following: nonscheduled extubation, need for reintubation, need for noninvasive positive pressure ventilation of the patient within 48h after extubation, delayed extubation, tracheostomy. |
| Abdelwahed 2019^[71]^ | Egypt | RICU, MV patients | 65 | 30 % | - Philips’s ultrasound (model CX_50_) | - Patient position: supine position (30 °- 45°).  - Timepoint of measurement: first five to ten minutes of the initial SBT.  - Maneuver: the probe was placed perpendicular to the chest wall in the mid-axillary line between the 8^th^ and 10^th^ intercostal spaces to observe the zone of apposition.  - Mode of US: M-mode.  - Type of probe: 10-15 MHz linear probe.  - Number of measurements: average of three measurements. | Sustained SB > 48h after extubation. |
| Alam  2022^[31]^ | Bangladesh | ICU, MV patients | 31 | 22.33 % | -Sonosite M-Turbo (FUJIFILM SonoSite, Bothell, WA, USA) | - Patient position: supine position (20 °- 40 °).  - Timepoint of measurement: at the start and after 10 min of a T-piece trial, and just before extubation  - Maneuver: the probe was placed between the 8^th^ and 10^th^ intercostal spaces.  - Mode of US: B-mode.  - Type of probe: 10 MHz linear probe.  - Number of measurements: average of three measurements. | Continuation of SB ≥ 48h after extubation |
| Ali  2017^[5]^ | Egypt | ICU, MV patients | 60 | 30 % | - Echo Blaster 128 Kit | - Patient position: supine position (0 °- 20°).  - Timepoint of measurement: NR  - Maneuver: thicknesses measured during tidal breathing, excluding smaller or deeper breaths. The probe was placed anterior subcostal between midclavicular and axillary lines.  - Mode of US: B-mode.  - Type of probe: 10 MHz linear transducer.  - Number of measurements: average of six measurements. | Ability to maintain SB ≥ 48h without any level of MV support. |
| Al Tayar 2022^[32]^ | Saudi Arabia | ICU, MV patients | 24 | 15 % | - CX50 (Philips, Amsterdam, the Netherlands) | - Patient position: NR  - Timepoint of measurement: after 30 min of SBT.  - Maneuver: thicknesses measured at end-inspiration and at end-expiration.  - Mode of US: NR  - Type of probe: linear probe 10 MHz.  - Number of measurements: average of three measurements. | No need for re-intubation, noninvasive ventilation, or death in the first 48h after extubation^a^ |
| Amara 2022^[33]^ | India | ICU, MV patients ready to wean | 81 | 27.5 % | - Philips CX 50 (Philips Healthcare, 3000 Minuteman Road, Andover, USA) | - Patient position: NR  - Timepoint of measurement: on the day of the first SBT  - Maneuver: the probe was placed at the zone of apposition at the mid-axillary line between the 8^th^ and 10^th^ intercostal space.  - Mode of US: NR.  - Type of probe: linear probe.  - Number of measurements: NR | Initiation of weaning to successful extubation after first SBT |
| Asmita 2022^[72]^ | India | ICU, MV patients | 100 | 44 % | - SonoScape S30 | - Patient position: supine position (45°)  - Timepoint of measurement: after 15-20 min during PSV.  - Maneuver: measurements performed during PSV at 2 cmH2O negative pressure trigger. Thicknesses were measures at full inspiration and expiration. The probe was placed at the zone of apposition, at the right anterior axillary line in the longitudinal plane, between 7^th^ and 9^th^ intercostal space.  - Type of probe: 7-18 MHz linear probe.  - Number of measurements: average of three measurements taken at least 10-15 min apart. | No reintubation within 48h of extubation^a^ |
| Baess  2016^[34]^ | Egypt | ICU and RICU, MV (intubated) patients | 30 | 30 % | - Ultrasound from Philips Healthcare  (Andover, Massachusetts, USA) | -Patient position: supine position (45°).  -Timepoint of measurement: during SBT.  - Maneuver: the probe was placed immediately below the costal margin at the midclavicular line or on the last two spaces at the anterior axillary line.  - Mode of US: B-mode.  - Type of probe: 2–4 MHz phased-array probe.  - Number of measurements: average of five measurements. | SB sustained > 48h following extubation. |
| Banerjee 2018^[35]^ | India | ICU, MV patients | 53 | 28 % | - Siemens Acuson X300 | - Patient position: NR  - Timepoint of measurement: after 20min of SBT.  - Maneuver: thicknesses measured at end-inspiration and at end-expiration. The probe was placed intercostally perpendicular to the chest wall in the 8^th^ or 9^th^ intercostal space between the anterior and midaxillary lines.  - Mode of US: NR  - Type of probe: linear probe.  - Number of measurements: NR | Stay in SB ≥ 48h after the extubation. |
| Blumhof  2016^[73]^ | USA | ICU, MV patients | 56 | 20 % | - Sonosite M-Turbo (FujiFilm, Tokyo, Japan) | - Patient position: supine position (20°- 40°).  - Timepoint of measurement: during PS weaning (PEEP = 5 cmH_2_O, PS = 5 cmH_2_O)  - Maneuver: the probe was placed at the zone of apposition located on the mid-axillary line between the 8^th^ and 10^th^ ribs.  - Mode of US: B-Mode  - Type of probe: linear 7–10 MHz probe.  - Number of measurements: average of three measurements. | Extubation in 48h after US. |
| Cavus 2022^[74]^ | Turkey | ICU, MV patients | 68 | 26% | - Philips ClearVue 550 system | - Patient position: supine position (20°- 40°).  - Timepoint of measurement: during PS ventilation (PEEP = 5 cmH2O, PS = 10 cmH2O), before 1h SBT- (T-piece trial).  - Maneuver: thicknesses measured at end-expiration and end-inspiration. The right hemidiaphragm was visualized in the midaxillary line between the 8th and 10th intercostal spaces, at the junction of the diaphragm and the rib cage.  - Mode of US: B-mode  - Type of probe: S1-4 MHz linear probe  - Number of measurements: average of three serial measurements | SB sustained for > 48h following extubation |
| DiNino  2014^[75]^ | USA | MICU, MV patients | 63 | 30 % | - LOGIQ Book, (GE  Healthcare, Waukesha, Wisconsin, USA) | - Patient position: supine position (20°- 40°)  - Timepoint of measurement: during SBT.  - Maneuver: thicknesses measured at end-expiration and end-inspiration. The probe was placed at the zone of apposition of the diaphragm and rib cage in the midaxillary line between the 8^th^ and 10^th^ intercostal spaces.  - Mode of US: B-mode.  - Type of probe: 7–10 MHz linear probe.  - Number of measurements: average of three to five measurements. | Extubation success + SB > 48h. |
| Dres 2021^[76]^ | France, Canada | MICU, SICU, RICU, MV patients | 122 | 15.6 % | - Sparq (Philips Healthcare, Bothell, WA, USA)  - Sonosite (Fujifilm Sonosite, Bothell, WA, USA) | - Patient position: NR  - Timepoint of measurement: within 2h after extubation with patients breathing spontaneously without noninvasive ventilation or high-flow oxygen therapy.  - Maneuver: thicknesses measured at end-expiration and end-inspiration. The probe was placed perpendicular to the right chest wall, at the midaxillary line between the 9th and 10th right intercostal spaces.  - Mode of US: M-mode  - Type of probe: 10-15 MHz linear probe.  - Number of measurements: average of three measurements | No re-intubation or death within the 7 days following planned extubation. |
| Eksombatchai 2023^[36]^ | Thailand | MICU and SICU, ready to wean MV patients | 130 | 26.2 % | - Sonosite M-Turbo (SonoSite Inc., Bothell, WA, USA) | - Patient position: supine (30°-45°)  - Timepoint of measurement: at 2h after the SBT.  - Maneuver: probe was placed at the zone of apposition, between the 8th and 10th intercostal spaces.  - Mode of US: M-mode  - Type of probe: 6-13 MHz linear probe  - Number of measurements: average of three consecutive tidal breaths. | No reintubation reintubation within 48h after extubation^a^. |
| Elgazzar  2019^[37]^ | Egypt | RICU, MV patients | 30 | 33 % | - Doppler ultrasound system, model S6 (SonoScape, Shanghai, China) | - Patient position: semi-sitting position.  - Timepoint of measurement: during SBT.  - Maneuver: NR  - Mode of US: B-mode and M-mode.  - Type of probe: NR  - Number of measurements: NR | Patient can continue SB for ≥ 48h without any MV support and without developing failure measures of SB. |
| Elshazly  2020^[38]^ | Egypt | RICU, MV patients | 62 | 21.5 % | - Transthoracic ultrasound (Logiq P3, GE Healthcare, Chicago, IL, USA) | - Patient position: semi-sitting position.  - Timepoint of measurement: during SBT.  - Maneuver: thicknesses measured during tidal breathing. The probe was placed between mid-clavicular line and anterior axillary line.  - Mode of US: B-mode.  - Type of probe: 7-12 MHz linear.  - Number of measurements: NR | Maintaining SB after extubation for > 48h. |
| Eltrabili  2019^[39]^ | Egypt | SICU, MV patients | 30 | 30.7 % | - Mindray model M7  ultrasound machine (Mindray Bio-Medical, Shenzhen, China) | - Patient position: supine position (45°).  - Timepoint of measurement: after SBT.  - Maneuver: the probe was placed perpendicular to the chest wall, in the 8^th^ or 9^th^ intercostal space, between the anterior and the mid-axillary lines to observe the zone of apposition.  - Mode of US: B-mode.  - Type of probe: 7.5–10 MHz linear.  - Number of measurements: NR | Patient stays in SB ≥ 48h after extubation. |
| Er 2021^[40]^ | Turkey | MICU, MV patients | 38 | 30%† | - Acuson X700, Siemens | - Patient position: supine position.  - Timepoint of measurement: within 36h after intubation.  - Maneuver: Measurements were done at end-expiration and end-inspiration during breaths with a tidal volume of 6-8ml/kg of ideal body weight. The probe was placed between anterior and mid-axillary lines at level of 9-10^th^ intercostal space.  - Mode of US: NR.  - Type of probe: 10.7 MHz linear probe.  - Number of measurements: average value of three consecutive measurements. | No reintubation or death within 7 days after extubation |
| Farghaly  2017^[3]^ | Egypt | RICU, MV patients | 54 | 34.2 % | - Samsung Medison Sono Ace R3 ultrasound system (Samsung company, Seoul, South Korea) | - Patient position: supine position (45°).  - Timepoint of measurement: during SBT.  - Maneuver: thicknesses measured during tidal breathing. The probe was placed at the zone of apposition perpendicular to the chest wall, in the 8^th^ and 9^th^ intercostal space between anterior axillary and mid-axillary lines.  - Mode of US: B-mode.  - Type of probe: 7 MHz probe.  - Number of measurements: average of three measurements. | Maintenance of SB > 48h following extubation. |
| Fossat 2022^[41]^ | France | MICU, MV patients | 79 | 30%† | - GE HealthCare | - Patient position: NR  - Timepoint of measurement: after 5 min of SBT.  - Maneuver: thicknesses measured at end-expiration and end-inspiration.  - Mode of US: NR  - Type of probe: NR  - Number of measurements: NR | No need for re-intubation or for the initiation of curative noninvasive ventilation (NIV) before or at day 7 after extubation |
| Genty 2022^[77]^ | France | Cardiothoracic ICU, MV patients | 50 | 50% | - Vivid 6 ultrasound probe (GE Healthcare, Chicago, Illinois) | - Patient position: NR  - Timepoint of measurement: during SBT.  - Maneuver: measurements were performed using the leading-edge method to minimize thickness overestimation due to inclusion of the peritoneal membrane. The breathing frequency curve was connected to the ultrasound device to locate end-expiration and end inspiration on the ultrasound images.  Diaphragm end-expiratory thickness was measured just before thickening started and end-inspiratory thickness at the inspiratory peak.  - Mode of US: NR  - Type of probe: 7-10-MHz linear probe.  - Number of measurements: the mean of measurements during 3 consecutive breaths. | Weaning failure was defined as SBT failure or extubation followed by reintubation  within 48h. |
| Gok 2021^[43]^ | Turkey | ICU, MV patients | 46 | 27.5 % | - Philips Affiniti (Philips, Amsterdam, Netherlands | - Patient position: NR  - Timepoint of measurement: during SBT (T-piece trial).  - Maneuver: thicknesses measured at end-expiration and end-inspiration. The probe was placed in the intercostal space at the zone of apposition.  - Mode of US: B-mode.  - Type of probe: 4-12 MHz linear probe.  - Number of measurements: average of five measurements. | No re-intubation or need for NIV within 48h after extubation^a^. |
| Gonzàlez-Aguirre  2019^[4]^ | Mexico | MICU or SICU, MV patients | 82 | 30 % | - GE Logiq XP (Soma Technology, Inc.) | - Patient position: supine position.  - Timepoint of measurement: NR  - Maneuver: thicknesses measured during tidal breathing, at maximal inspiration and expiration. The probe was placed at 8^th^ or 9^th^ intercostal space at mid-axillary line.  - Mode of US: B-mode.  - Type of probe: 10 MHz linear probe.  - Number of measurements: NR | No reinsertion of endotracheal tube for > 48h after extubation^a^. |
| Haaksma 2021^[78]^ | The Netherlands | ICU, MV patients | 83 | 30% | - Philips CX50 (Andover, Massachusetts; 100–240 V, 2.65 A, maximum frame rate 755 frames/s, dynamic range 170 dB) | - Patient position: NR  - Timepoint of measurement: at the end of 1h SBT (PS and PEEP < 10 cmH2O)  - Maneuver: thickneses measured at end-expiration and e,d-inspiration. The probe was placed on the mid-axillary line in the zone of apposition  - Type of probe: 4-12 MHz linear probe.  - Number of measurements: three video of three respiratory cycles. For each video, the respiratory cycle where the pleural and peritoneal line could be most clearly be delineated was retained. The average of three end-expiratory and three end-inspiratory values were used to calculate DTF. | No need for reintubation or rescue noninvasive ventilation within 48h after extubation^a^. |
| Kaur 2022^[50]^ | India | ICU, MV patients ready to wean | 50 | 23.75% | - FUJIFILM ((SonoSite Inc., Bothell, WA, USA) | - Patient position: supine position.  - Timepoint of measurement: during SBT.  - Maneuver:the probe was placed in cranio-caudal  direction in the 9th or 10th intercostal space near the  mid-axillary line and angled perpendicular to the chest  wall.  - Mode of US: M-mode.  - Type of probe: 10 MHz linear probe.  - Number of measurements: average of three values. | Ability to sustain SB after removal of the endotracheal tube or tracheostomy tube > 48 hours, without the need for re-intubation^a^. |
| Kundu 2022^[79]^ | India | ICU tertiary care university teaching hospital | 60 | 26% | NR | - Patient position: supine position (30 – 45°).  - Timepoint of measurement: before and after SBT.  - Maneuver: NR  - Mode of US: NR  - Type of probe:NR  - Number of measurements: average of three measurements. | The requirement of non-invasive or invasive mechanical ventilation over the ensuing 48h. |
| Lalwani 2022^[80]^ | India | ICU, MV patients with ARF | 54 | 29.71 % | - Micromaxx ultrasound machine (Sonosite Inc., Bothell, WA, USA) | - Patient position: NR  - Timepoint of measurement: during SBT.  - Maneuver: diaphragm thickness was measured at end-expiration and end-inspiration. The right diaphragm was imaged at the zone of apposition in the mid-axillary line between the 6th and 10th intercostal spaces. Similarly, the left diaphragm was imaged between the 6th and 10th intercostal spaces in the mid to posterior axillary line.  - Mode of US: B-mode  - Type of probe: 6-13 MHz linear probe.  - Number of measurements: average of three measurements. | Spontaneous breathing for more than 48 hours without needing MV or reintubation. |
| Li 2021^[53]^ | China | ICU, elderly patients (<= 65years) | 101 | 30 % | - Mindray probe | - Patient position: NR  - Timepoint of measurement: during SBT.  - Maneuver: The right hemidiaphragm was imaged at the zone of apposition of the diaphragm and rib cage on the mid-axillary line between the 8th and 10th intercostal spaces.  - Mode of US: B-mode.  - Type of probe: 6–13 MHz linear probe.  - Number of measurements: NR | Extubation success was defined as sustained SB for more than 48h following extubation without noninvasive positive-pressure ventilation (NPPV) or invasive ventilation. |
| Mawla 2022^[55]^ | Egypt | ICU, MV patients | 90 | 13.5 % | - Philips HD11XE | - Patient position: supine position (30 – 45°).  - Timepoint of measurement: during SBT.  - Maneuver: probe was placed intercostally perpendicular to the chest wall in the 8th or 9th intercostal space between the anterior and mid-axillary line.  - Mode of US: B-mode.  - Type of probe: 9 MHz linear probe.  - Number of measurements: NR | Able to maintain their own breathing for 48h after extubation without any level of ventilator support. |
| McCool  2020^[2]^ | USA | MICU, MV patients | 32 | 30 % | - LOGIQ Book (GE  Healthcare, Waukesha, Wisconsin, USA) | - Patient position: NR  - Timepoint of measurement: first 5 to 10 min of SBT.  - Maneuver: thicknesses measured at end-expiration and end-inspiration. The probe placed at the right hemidiaphragm in the mid-axillary line between the 8^th^ and 10^th^ intercostal spaces.  - Mode of US: B-mode.  - Type of probe: 7–10 MHz linear probe.  - Number of measurements: average of three to five measurements. | NR |
| Mohamed 2021^[56]^ | Egypt | ICU, MV patients | 80 | 32.82 % | NR | - Patient position: supine position (20 - 40°).  - Timepoint of measurement: after SBT success.  - Maneuver: the right hemidiaphragm was imaged at the zone of apposition of the diaphragm and rib cage in the midaxillary line between the 8th and 10th intercostal spaces.  - Mode of US: B-mode.  - Type of probe: 7–10-MHz linear probe.  - Number of measurements: average of at least three measurments | Successful extubation was defned as maintenance of SB > 48h following extubation. |
| Osman  2017^[57]^ | Egypt | ICU, MV patients | 68 | 28 % | - Logic E9 (GE) and Honda electronics HS-2100 Por-  table ultrasound machine. | - Patient position: supine position (45°).  - Timepoint of measurement: during SBT.  - Maneuver: thicknesses measured at end-inspiration and end-expiration. The probe was placed intercostally perpendicular to the chest wall in the 8^th^ or 9th intercostal spaces between the anterior and mid-axillary line.  - Mode of US: NR  - Type of probe: 9–11 MHz linear probe.  - Number of measurements: NR | No requirement of reintubation or re-ventilation within 48h of extubation. |
| Pirompanich 2018^[81]^ | Thailand | MICU and medical wards, MV patients ready to wean | 34 | 26 % | - LOGIQ C5 Premium (GE Medical System, China) | - Patient position: supine position (45°).  - Timepoint of measurement: after 1h SBT.  - Maneuver: thicknesses measured at TLC and RV. The probe was placed vertical to the chest wall, at the 8^th^ or 9^th^ intercostal spaces, between the anterior axillary and the mid-axillary lines, to observe the zone of apposition.  - Mode of US: B-mode.  - Type of probe: 10-MHz linear probe.  - Number of measurements: average of three measurements. | Ability to maintain SB ≥ 48h, without MV support and without criteria for failure of SB. |
| Saad 2022^[59]^ | Egypt | ICU, MV patients | 64 | 37 % | PHILIPS HD5 release 2.1 (distributed by PHILIPS healthcare, Bothell, WA, USA) @2011Koninklijke Philips N.V. | - Patient position: supine position.  - Timepoint of measurement: after 30 min during a T-piece trial.  - Maneuver: during deep breathing, the probe was placed perpendicular to the chest wall or with angle not less than 70 degrees according difficulty of the case, in the eighth or ninth intercostal space, between the anterior axillary and the midaxillary lines. Diaphragmatic thickness was subsequently measured at the zone of apposition, at both total lung capacity and residual volume.  - Mode of US: M-mode.  - Type of probe: 7–11 MHz linear probe.  - Number of measurements: average of six measurements. | Failure of weaning was considered when patient needed MV during SBT and patient was reintubated and ventilated or needed non-invasive ventilation (NIV) within 48h. |
| Samanta  2017^[61]^ | India | MICU and SICU, MV patients ready to wean | 64 | 24.5 % | - Fujifilm SonoSite, Inc. | - Patient position: supine position (45°).  - Timepoint of measurement: during the first T-piece trial.  - Maneuver: the probe was placed in the zone of apposition of the diaphragm and ribcage in the mid-axillary line between the 8^th^ and 10^th^ intercostal space.  - Mode of US: M-mode.  - Type of probe: 3.5-5 MHz curvilinear.  - Number of measurements: average of three measurements. | Patients who proceeded from initiation of weaning to extubation success on their first SBT without any difficulty were categorized as simple weaning. |
| Saravanan 2022^[62]^ | India | ICU, MV patients | 200 | 37 % | - GE LogiQ machine, China | - Patient position: supine position (45°)  - Timepoint of measurement: before extubation.  - Maneuver: the probe was placed between the anterior and mid-axillary line on the right side at the level of the eighth or ninth intercostal spaces to visualize the diaphragm muscle and the  zone of apposition of the diaphragm with the pleura.  - Mode of US: M-mode  - Type of probe: 8-13 MHz linear probe  - Number of measurements: NR | Ability of the patient to tolerate spontaneous breathing without the use of non-invasive ventilation for at least 48h after extubation. |
| Shamil 2022^[24]^ | India | ICU, Mv patients ready to wean | 50 | 30%† | NR | - Patient position: position: supine position (30°-45°).  - Timepoint of measurement: during SBT.  - Maneuver: measurements performed during tidal breathing while patients ventilated with PSV (PEEP: 5 cmH2O, PS: 5 cmH2O); diaphragmatic thickness was measured during both inspiration and expiration.  - Mode of US: NR  - Type of probe: 10 MHz linear probe.  - Number of measurements: NR | No reintubation or need of noninvasive ventilation (NIV) for the patient within 48 hours after extubation. |
| Soliman  2019^[82]^ | Egypt | ICU, MV patients | 100 | 29.5 % | - TOSHIBA  ACUSON X 300x | - Patient position: NR  - Timepoint of measurement: after SBT.  - Maneuver: thicknesses measured at TLC and RV. The probe was placed in the 8^th^ intercostal space, perpendicular to the chest wall between the anterior axillary and the mid axillary lines, to see the zone of apposition of the diaphragm.  - Mode of US: B-mode and M-mode.  - Type of probe: 10 MHz linear probe.  - Number of measurements: average of three measurements. | Ability to stay in SB ≥ 48h after extubation. |
| Song 2022^[63]^ | China | ICU, MV patients | 110 | 30.09% | M-turbo; FUJIFILM (Sonosite, Washington, USA) | - Patient position: supine (30°-45°)  - Timepoint of measurement: after 30 min from the beginning of the SBT or immediately before returning to the initial ventilator settings in case of SBT failure.  - Maneuver: the probe was placed perpendicular to the chest wall, in the 8th or 9th intercostal space, between the anterior axillary and midaxillary lines, to observe the zone of apposition of the muscle 0.5-2 cm below the costophrenic sinus.  - Mode of US: M-mode  - Type of probe: 10 MHz linear probe  - Number of measurements: average of three values | SBT success and ability to maintain SB > 48h without needing non-invasive or invasive ventilation. |
| Spadaro 2021^[64]^ | Italy | ICU, MV patients | 62 | 30% | M-Turbo, SonoSite, Inc., USA | - Patient position: supine position (30 – 45°).  - Timepoint of measurement: day 3 after start of weaning.  - Maneuver: as described in Vivier et al. 2012 and Kim et al. 2011, by using the right intercostal space approach.  - Mode of US: M-mode.  - Type of probe: 12-MHz linear probe.  - Number of measurements: NR | Reintubation^a^ |
| Tenza-Lozano 2018^[26]^ | Spain | ICU, MV patients | 69 | 24 % | - Micromax® Sonosite | - Patient position : semi-decubitus position (20˚– 40˚).  - Timepoint of measurement: during SBT.  - Maneuver: thicknesses measured at end-expiration and end-inspiration during tidal breathing. The probe was placed in the zone of apposition, on the midaxillary line between the 8^th^ and 10^th^ intercostal spaces.  - Mode of US: B-mode and M-mode.  - Type of probe: 7–10 MHz linear probe.  - Number of measurements: average of three measurements. | Patients were extubated and SB > 48h. |
| Thabet  2020^[65]^ | Egypt | RICU, MV patients | 40 | 37 % | - MEDESION SONOACE R3 portable ultrasound  (2010; Samsung Company, Seoul, Korea) | - Patient position: supine position (45°).  - Timepoint of measurement: immediately after the start of SBT.  - Maneuver: thicknesses measured at end-inspiration and end-expiration. The probe was placed in the zone of apposition, on the midaxillary line between the 8th and 10th intercostal spaces.  - Mode of US: M-mode.  - Type of probe: 7.5 MHz linear probe.  - Number of measurements: average of three measurements. | Patients who did not require any re-use of MV, either invasive or noninvasive ≥ 48h. |
| Trifi 2021^[83]^ | Tunis | MICU, MV patients | 30 | 32.70% | Aloka-ARIETTA V60 [manufacturing Year: 2014, Company: Hitachi, Ltd, manufacturing country Chiyoda, Tokyo, Japan] | - Patient position: NR  - Timepoint of measurement: minimum of 48h and maximum of 5 days since beginning of MV.  - Maneuver: right hemi-diaphragm measured at zone of apposition at the level of seventh to ninth intercostal spaces. The probe was inclined such that the ultrasound beam reaches perpendicularly the posterior part of the diaphragm.  - Mode of US: B-mode.  - Type of probe: 10-MHz linear probe.  - Number of measurements: NR | NR |
| Varón-Vega 2021^[1]^ | Colombia | ICU, MV patients | 84 | 30% | Sonocare ultrasound system (Sonosite EDGE 03VRYF) | - Patient position: supine position (45°)  - Timepoint of measurement: after 30min of SBT.  - Maneuver: the transducer was positioned just below the ribcage, between the clavicular midline and the anterior axillary line. The ultrasound beam was directed cephalad, perpendicular to the posterior third of the diaphragm.  - Mode of US: M-mode.  - Type of probe: 6-13 MHz probe.  - Number of measurements: NR | Successful extubation was defined as the capacity to maintain SB > 48h without ventilatory assistance after extubation. |
| Vetrugno 2022^[84]^ | Italy | ICU, COVID-19 patients ready to be weaned | 57 | 30%† | NR | - Patient position: supine (10°-15°)  - Timepoint of measurement: within 24h after the start of weaning.  - Maneuver: the right hemidiaphragm was assessed. Thicknesses were measured at end-inspiration and end-expiration while patients were ve,tilated with PSV with standardized settings (PEEP: 5 cmH2O, PS: 8 cmH2O, FiO2<50%).  - Mode of US: M-mode  - Type of probe: linear probe.  - Number of measurements: average of three consecutive measurements. | SBT success and no need of reintubation within 48h following extubation^a^. |
| Vivier 2019^[67]^* | France | ICU, Ready to wean patients at risk of extubation failure | 164 | 30 % | - Vivid S6 (General Electric Healthcare, Little Chalfont, Buckinghamshire, England) or  - CX-50 (Philips Ultrasound, Bothell, WA, USA) | - Patient position: supine position (45°).  - Timepoint of measurement: during SBT.  - Maneuver: thicknesses measured during tidal breathing. The probe was placed on the zone of apposition perpendicularly to the chest wall.  - Mode of US: M-mode.  - Type of probe: 10-MHz linear probe.  - Number of measurements: average of three consecutive measurements. | No reintubation or death for >7 days after planned extubation^a^. |
| Xu 2022^[68]^ | China | ICU, MV patients | 96 | 27.39 % | - Vivid7 and Vivid6 (GE, USA) | - Patient position: supine position (45°)  - Timepoint of measurement: during the first SBT.  - Maneuver: The probe was placed between the right anterior axillary line and the midaxillary line, perpendicular to the 8th and 9th ribs where the zone of apposition of the diaphragm could be visualized.  - Mode of US: M-mode  - Type of probe: 4-13 MHz linear probe (Vivid6) and 4-11 MHz linear probe (Vivid7).  - Number of measurements: NR | No reintubation within 48 h or no death within 7 days after extubation. |
| Yoo  2018^[69]^ | Republic of Korea | MICU or SICU, MV patients ready to wean | 60 | 30 % | - M-Turbo, (Fujifilm, SonoSite Inc., Bothell, WA, USA) | - Patient position: NR  - Timepoint of measurement: during SBT.  - Maneuver: thicknesses measured at end-expiration and end-inspiration. The probe was placed in the zone of apposition of the diaphragm and rib cage in the mid-axillary line between the 8^th^ and 10^th^ intercostal spaces.  - Mode of US: B-mode.  - Type of probe: 6-13 MHz linear probe.  - Number of measurements: NR | Sustained SB > 48h following extubation without NIV. |
| **Diaphragm thickness end-expiratory, Tdi_ee_** | | | | | | | |
| **Study** | **Country** | **Setting and population** | **Sample size (n)** | **Threshold** | **Equipment used** | **Assessment protocol** | **Definition of weaning success^b^** |
| Ali  2017^[5]^ | Egypt | ICU, MV patients | 60 | 2 mm | Echo Blaster 128 Kit | - Patient position: supine position (0 °-20 °).  - Timepoint of measurement: NR  - Maneuver: measurements at end-expiration during tidal breathing, excluding smaller or deeper breaths. The probe was placed anterior subcostal between midclavicular and axillary lines.  - Mode of US: B-mode.  - Type of probe: 10 MHz linear transducer.  - Number of measurements: average of six measurements. | Ability to maintain SB for 48h without any level of MV support. |
| Baess 2016^[34]^ | Egypt | ICU and RICU, MV (intubated) patients | 30 | 3.30mm | Ultrasound from Philips Healthcare  (Andover, Massachusetts, USA) | - Patient position: supine position (45°).  - Timepoint of measurement: during SBT.  - Maneuver: measurements at end-expiration. The probe was placed immediately below the costal margin at the midclavicular line or on the last two spaces at the anterior axillary line.  - Mode of US: B-mode.  - Type of probe: 2–4 MHz phased-array probe.  - Number of measurements: average of five measurements. | SB sustained > 48h following extubation. |
| Cavus 2022^[74]^ | Turkey | ICU, MV patients | 68 | 6.3 mm | - Philips ClearVue 550 system | - Patient position: supine position (20°- 40°).  - Timepoint of measurement: during PS ventilation (PEEP = 5 cmH2O, PS = 10 cmH2O), before 1h SBT- (T-piece trial).  - Maneuver: measurements at end-expiration. The right hemidiaphragm was visualized in the midaxillary line between the 8th and 10th intercostal spaces, at the junction of the diaphragm and the rib cage.  - Mode of US: B-mode  - Type of probe: S1-4 MHz linear probe  - Number of measurements: average of three serial measurements | SB sustained > 48h following extubation |
| DiNino 2014^[75]^ | USA | MICU, MV patients | 63 | 1.70mm | LOGIQ Book (GE  Healthcare, Waukesha, Wisconsin, USA) | - Patient position: supine position (20°- 40°).  - Timepoint of measurement: during SBT.  - Maneuver: measurements at end-expiration. The probe was placed at the zone of apposition of the diaphragm and rib cage in the midaxillary line between the 8^th^ and 10^th^ intercostal spaces.  - Mode of US: B-mode.  - Type of probe: 7–10 MHz linear probe.  - Number of measurements: average of three to five measurements. | SB > 48h after extubation. |
| Er 2021^[40]^ | Turkey | MICU, MV patients | 38 | 3.30 mm† | - Acuson X700, Siemens | - Patient position: supine position.  - Timepoint of measurement: within 36h after intubation.  - Maneuver: Measurements were done at end-expiration during breaths with a tidal volume of 6-8ml/kg of ideal body weight. The probe was placed between anterior and mid-axillary lines at level of 9-10^th^ intercostal space.  - Mode of US: NR.  - Type of probe: 10.7 MHz linear probe.  - Number of measurements: average value of three consecutive measurements. | No reintubation or death within 7 days after extubation |
| Farghaly 2017^[3]^ | Egypt | RICU, MV patients | 54 | 10.5mm | Samsung Medison Sono Ace R3 ultrasound system (Samsung company, Seoul, South Korea) | - Patient position: supine position (45°).  - Timepoint of measurement: during SBT.  - Maneuver: measurements at end-expiration during tidal breathing. The probe was placed at the zone of apposition perpendicular to the chest wall, in the 8^th^ and 9^th^ intercostal space between anterior axillary and midaxillary line.  - Mode of US: B-mode.  - Type of probe: 7 MHz probe.  - Number of measurements: average of three measurements. | Maintenance of SB > 48h following extubation. |
| Fossat 2022^[41]^ | France | MICU, MV patients | 79 | 3.30mm† | - GE HealthCare | - Patient position: NR  - Timepoint of measurement: after 5 min of SBT.  - Maneuver: measurements at end-expiration during quiet breathing.  - Mode of US: NR  - Type of probe: NR  - Number of measurements: NR | No need for re-intubation or for the initiation of curative noninvasive ventilation (NIV) before or at day 7 after extubation |
| Lalwani 2022^[80]^ | India | ICU, MV patients with ARF | 54 | 1.78 mm | - Micromaxx ultrasound machine (Sonosite Inc., Bothell, WA, USA) | - Patient position: NR  - Timepoint of measurement: during SBT.  - Maneuver: diaphragm thickness was measured at end-expiration and end-inspiration. The right diaphragm was imaged at the zone of apposition in the mid-axillary line between the 6th and 10th intercostal spaces. Similarly, the left diaphragm was imaged between the 6th and 10th intercostal spaces in the mid to posterior axillary line.  - Mode of US: B-mode  - Type of probe: 6-13 MHz linear probe.  - Number of measurements: average of three measurements. | Spontaneous breathing for more than 48 hours without needing MV or reintubation. |
| Mohamed 2021^[56]^ | Egypt | ICU, MV patients | 80 | 15.5 mm | NR | - Patient position: head of the bed elevated between 20 and 40°  - Timepoint of measurement: after SBT success  - Maneuver: the right hemidiaphragm was imaged at the zone of apposition of the diaphragm and rib cage in the midaxillary line between the 8th and 10th intercostal spaces.  - Mode of US: B-mode  - Type of probe: linear probe, 7–10-MHz  - Number of measurements: mean of at least 3 measurements | Successful extubation was defned as maintenance of SB > 48h following extubation |
| Thabet 2020^[65]^ | Egypt | RICU, MV patients | 40 | 2.60mm | Medesion SonoAce R3 portable ultrasound  (2010; Samsung Company, Seoul, Korea) | - Patient position: supine position (45°).  - Timepoint of measurement: immediately after the start of SBT.  - Maneuver: measurements at the end of expiration. The probe was placed in the zone of apposition, on the midaxillary line between the 8^th^ and 10^th^ intercostal spaces.  - Mode of US: M-mode.  - Type of probe: 7.5 MHz linear probe.  - Number of measurements: average of three measurements. | Patients who did not require any re-use of MV, either invasive or noninvasive, ≥ 48h. |
| Vetrugno 2022^[84]^ | Italy | ICU, COVID-19 patients ready to be weaned | 57 | 3.30mm† | NR | - Patient position: supine (10°-15°)  - Timepoint of measurement: within 24h after the start of weaning.  - Maneuver: the right hemidiaphragm was assessed. Thicknesses were measured at end-inspiration and end-expiration while patients were ve,tilated with PSV with standardized settings (PEEP: 5 cmH2O, PS: 8 cmH2O, FiO2<50%).  - Mode of US: M-mode  - Type of probe: linear probe.  - Number of measurements: average of three consecutive measurements. | SBT success and no need of reintubation within 48h following extubation^a^. |
| **Diaphragm thickness end-expiratory, Tdi_ei_** | | | | | | | |
| **Study** | **Country** | **Setting and population** | **Sample size (n)** | **Threshold** | **Equipment used** | **Assessment protocol** | **Definition of weaning success^b^** |
| Baess 2016^[34]^ | Egypt | ICU and RICU, MV (intubated) patients | 30 | 4.1 mm | - Ultrasound from Philips Healthcare,  (Andover, Massachusetts, USA) | - Patient position: supine position (45°).  - Timepoint of measurement: during SBT.  - Maneuver: measurements at end-inspiration. The probe was placed immediately below the costal margin at the midclavicular line or on the last two spaces at the anterior axillary line.  - Mode of US: B-mode.  - Type of probe: 2–4 MHz phased-array probe.  - Number of measurements: average of five measurements. | SB sustained > 48h following extubation. |
| Cavus 2022^[74]^ | Turkey | ICU, MV patients | 68 | 9.7 mm | - Philips ClearVue 550 system | - Patient position: supine position (20°- 40°).  - Timepoint of measurement: during PS ventilation (PEEP = 5 cmH2O, PS = 10 cmH2O), before 1h SBT- (T-piece trial).  - Maneuver: measurements at end-expiration. The right hemidiaphragm was visualized in the midaxillary line between the 8th and 10th intercostal spaces, at the junction of the diaphragm and the rib cage.  - Mode of US: B-mode  - Type of probe: S1-4 MHz linear probe  - Number of measurements: average of three serial measurements | SB sustained > 48h following extubation |
| Er 2021^[40]^ | Turkey | MICU, MV patients | 38 | 9.7 mm† | - Acuson X700, Siemens | - Patient position: supine position.  - Timepoint of measurement: within 36h after intubation.  - Maneuver: Measurements were done at end-expiration and end-inspiration during breaths with a tidal volume of 6-8ml/kg of ideal body weight. The probe was placed between anterior and mid-axillary lines at level of 9-10^th^ intercostal space.  - Mode of US: NR.  - Type of probe: 10.7 MHz linear probe.  - Number of measurements: average value of three consecutive measurements. | No reintubation or death within 7 days after extubation |
| Farghaly 2017^[3]^ | Egypt | RICU, MV patients | 54 | 21 mm | - Samsung Medison SonoAce R3 ultrasound system (Samsung company, Seoul, South Korea) | - Patient position: supine position (45°).  - Timepoint of measurement: during SBT.  - Maneuver: measurements at end-inspiration during tidal breathing. The probe was placed at the zone of apposition perpendicular to the chest wall, in the 8^th^ and 9^th^ intercostal space between anterior axillary and midaxillary line.  - Mode of US: B-mode.  - Type of probe: 7 MHz probe.  - Number of measurements: average of three measurements. | Maintenance of SB > 48h following extubation. |
| Fossat 2022^[41]^ | France | MICU, MV patients | 79 | 9.7 mm† | - GE HealthCare | - Patient position: NR  - Timepoint of measurement: after 5 min of SBT.  - Maneuver: measurements at end-expiration during quiet breathing.  - Mode of US: NR  - Type of probe: NR  - Number of measurements: NR | No need for re-intubation or for the initiation of curative noninvasive ventilation (NIV) before or at day 7 after extubation |
| Mohamed 2021^[56]^ | Egypt | ICU, MV patients | 80 | 21 mm | NR | - Patient position: supine position (20 – 40°)  - Timepoint of measurement: after SBT success.  - Maneuver: the right hemidiaphragm was imaged at the zone of apposition of the diaphragm and rib cage in the midaxillary line between the 8th and 10th intercostal spaces.  - Mode of US: B-mode.  - Type of probe: 7–10-MHz linear probe.  - Number of measurements: average of at least 3 measurements. | maintenance of SB > 48h following extubation |
| Thabet 2020^[65]^ | Egypt | RICU, MV patients | 40 | 2.6 mm | - Medesion SonoAce R3 portable ultrasound  (2010; Samsung Company, Seoul, Korea | - Patient position: supine position (45°).  - Timepoint of measurefment: immediately after the start of SBT  - Maneuver: measurements at the end of inspiration. The probe was placed in the zone of apposition, on the midaxillary line between the 8^th^ and 10^th^ intercostal spaces.  - Mode of US: M-mode.  - Type of probe: 7.5 MHz linear probe.  - Number of measurements: average of three measurements. | Patients who did not require any reuse of MV, either invasive or noninvasive, ≥ 48h. |
| Vetrugno 2022^[84]^ | Italy | ICU, COVID-19 patients ready to be weaned | 57 | 9.7mm† | NR | - Patient position: supine (10°-15°)  - Timepoint of measurement: within 24h after the start of weaning.  - Maneuver: the right hemidiaphragm was assessed. Thicknesses were measured at end-inspiration and end-expiration while patients were ve,tilated with PSV with standardized settings (PEEP: 5 cmH2O, PS: 8 cmH2O, FiO2<50%).  - Mode of US: M-mode  - Type of probe: linear probe.  - Number of measurements: average of three consecutive measurements. | SBT success and no need of reintubation within 48h following extubation^a^. |
| **Airway occlusion pressure, P0.1** | | | | | | | |
| **Study** | **Country** | **Setting and population** | **Sample size (n)** | **Threshold** | **Equipment used** | **Assessment protocol** | **Definition of weaning success^b^** |
| Azeredo  2017^[85]^ | Brazil | ICU, MV patients | 331 | 3.1 cmH2O | - Software of ventilator Evita 2 (Draëger, Lübeck, Germany) | - Patient position: NR  - Timepoint of measurement: before the SBT, during PSV (PEEP=5 cmH_2_O, PS=7cmH_2_O).  - Maneuver: NR  - Number of measurements: average of three consecutive measurements with intervals of 15 s. | Sustained SB >48h after extubation |
| Capdevila 1995^[8]^ | France | ICU, MV patients ready to wean | 67 | 5.0 cmH2O | - A side port proximal to the endotracheal or tracheostomy tube with a differential pressure transducer (MLR 2, Les Clayes, France). The signal was printed using an ultrarapid ink jet printer (Oscillomink, Siemens, Sweden). | - Patient position: supine position (30 ˚- 45˚).  - Timepoint of measurement: during a 20-min T-piece trial.  - Maneuver: occlusion at end of expiration by means of a syringe  - Number of measurements: average of three measurements, each separated by the time needed for a return to resting ventilation levels. | Reinstitution of MV was not necessary in the 48h following extubation. |
| Chittock  2000^[86]^ | Canada | ICU, MV patients ready to be extubated | 68 | 7.5 cmH_2_O | - Low-compliance cannula connected to a pressure transducer (Ohmeda DT-4812, Singapore) placed in the MV circuit between the patient end of the Y connector tubing and the endotracheal tube | - Patient position: NR  - Timepoint of measurement: during PS 0 cmH_2_O and CPAP 5 cmH_2_O.  - Maneuver: airway was briefly occluded at the inspiratory port of MV to measure P0.1 .  - Number of measurements: average of three measurements separated by 15 s. | Sustained SB without reintubation > 24h after extubation with PS 0 cmH_2_O. |
| Conti  2004^[11]^ | Italy | ICU, MV patients | 50 | 4.0 cmH2O | NR | - Patient position: NR  - Timepoint of measurement: during the first 2 min after discontinuation of MV.  - Maneuver: occlusion of the inspiratory line during expiration by inflating a rubber balloon with a syringe connected with a 30-cm noncompliant line^[12]^.  - Number of measurements: NR | Sustained SB >48h after extubation |
| de Souza  2012^[14]^ | Brazil | ICU, MV patients, ready to wean | 103 | 2.3 cmH2O | - Unidirectional valve method and a digital vacuometer (MVD 300, Globalmed, Porto Alegre, Rio Grande do Sul, Brazil) | - Patient position: : supine position (45˚).  - Timepoint of measurement: before SBT.  - Maneuver: patients were disconnected from MV and 20 s occlusion was applied using a unidirectional valve method. Pre-oxygenation was used beforehand.  - Number of measurements: average of P0.1 of the first three breathing cycles. | Sustained SB > 48h after withdrawal from MV |
| Fernandez 2004^[87]^ | Spain | MICU and SICU, MV patients ready to wean | 57 | 2.8 cmH2O | - Ventilator Evita 2 and Evita 4 (Draëger, Lübeck, Germany) | - Patient position: NR  - Timepoint of measurement: during first min of 30 min SBT, under MV with PS=7cmH_2_O and PEEP = 0cmH2O, flow trigger 1L/min, no use of automatic tube compensation.  - Maneuver: P0.1 recorded from the ventilator display.  - Number of measurements: NR | No need for reintubation within 48h after extubation^a^ |
| Hurtado  2001^[88]^ | Uruguay | ICU, MV patients ready to wean | 19 | 3.0 cmH2O | - Pneumotachograph (Hewlett-Packard21071B) connected to an airway flow transducer (Hewlett-Packard 47304A | - Patient position: NR  -Timepoint of measurement: after SBT.  - Maneuver: measured during T-tube MV by occluding the airway with a manually operated pneumatic valve.  - Number of measurements: average of three to five measurements. | Patients who do not required to be re-intubated or MV within 24h of extubation |
| Kaur 2022^[50]^ | India | ICU, MV patients ready to wean | 50 | 4.7 cmH2O | NR | - Patient position: supine position.  - Timepoint of measurement: during SBT.  - Maneuver: NR  - Number of measurements: average of three measurements. | Ability to sustain SB after removal of the endotracheal tube or tracheostomy tube > 48 hours, without the need for re-intubation^a^. |
| Liu  2010^[89]^ | China | MICU, MV patients | 91 | 3.7 cmH2O | - Evita-4 or XL ventilator (Draëger, Lübeck, Germany) | - Patient position: supine position (30 ˚- 45˚).  - Timepoint of measurement: at 30 min of SBT.  - Maneuver: NR  - Number of measurements: average of three measurements separated by an interval not shorter than 15 s. | Sustained SB > 48h after extubation. |
| Montgomery 1987^[90]^ | USA | ICU, MV patients, recovering from acute respiratory failure of a variety of medical and surgical aetiologies, ready to wean | 14 | 3.6 cmH2O† | - A low resistance one-way valve (Hans Rudolph, internal dead space = 18 ml) was attached to the patients endotracheal tube.  - A storage oscilloscope was used for visual measurements. | - Patient position: supine position.  - Timepoint of measurement: Last 3 min of a 20-min T-piece trial  - Maneuver: Occlusion of the- inspiratory portion of the patient-ventilator Y-connector by means of a pneumatically driven valve operated by a solenoid. Occlusion performed randomly every 7 to 20 seconds.  - Number of measurements: average of at least ten measurements. | No development of progressive hypercapnia during the T-piece trial and no reinstitution of MV within 24h^a^ |
| Okamoto 1990^[91]^ | Japan | ICU, MV patients after major surgery or severe burns | 33 | 3.5 cmH2O | - Airway pressure catheter (2.0 mm internal diameter and 2.7 mm outer diameter) was connected to a pressure transducer (P23ID, Statham Instruments, U.S.A.)  - airway pressure was continuously recorded on a polygraph (Nihon Koden, Japan) at a speed of 50 mm/sec. | - Patient position: supine position (30 ˚- 45˚).  - Timepoint of measurement: during SB through T-piece breathing circuit.  - Maneuver: the connector of the endotracheal tube was tightly occluded with the tip of the finger, starting from end-expiratory phase until 0.4-0.6 seconds of the inspiratory phase.  - Number of measurements: average of at least five measurements. | No need of MV within 24h after extubation of discontinuation of CPAP^a^ |
| Rivera  1997^[92]^ | USA | ICU, MV ready to wean patients | 40 | 4.5 cmH_2_O | - A bidirectional pneumotachograph, the VarFlex flow transducer (Al- lied Healthcare Products) | - Patient position: supine position (30 ˚- 45˚).  - Timepoint of measurement: during CPAP 3-4 cmH_2_0 + PS 5cmH_2_0  - Maneuver: NR  - Number of measurements: NR | NR |
| Sassoon  1993^[23]^ | USA | ICU, MV patients, recovering from acute respiratory failure of various aetiologies, ready to wean | 45 | 5.5 cmH2O | - A side port proximal to the endotracheal tube with a differential pressure transducer (MP45 ± 100 cm H20 ; Validyne Corp., Northridge, CA)  - an unidirectional balloon occlusion valve (Hans Rudolph, Kansas City, MO) | - Patient position: supine position (30 ˚- 45˚).  - Timepoint of measurement: after 5min of 1h 5cmH_2_O CPAP trial  - Maneuver: the inspiratory line was occluded for less than 500 ms at intervals of more than 15 s.  - Number of measurements: average of three measurements not falling outside twice the standard error. | Ability to complete the 1-h trial or to complete the trial without presenting siqns of cardiorespiratory distress within 48h of discontinuation from MV^a^ |
| Thabet  2020^[65]^ | Egypt | ICU, MV patients | 40 | 3.59 cmH_2_O | - Puritan-Bennett ventilator (NPB 840; Puritan Bennett/Covidien, Carlsbad, California, USA) | - Patient position: NR  - Timepoint of measurement: during PEEP < 5cmH_2_0, PS = 8cmH_2_0.  - Maneuver: P0.1 was measured using the ventilator software  - Number of measurements: average of five measurements. | Patients who did not require any re-use of MV, either invasive or noninvasive, ≥ 48h |
| Vallverdu 1998^[93]^ | Spain | MICU and SICU, MV patients ready to wean | 217 | 4.5 cmH2O | - Fleisch no. 2 pneumotachograph (Metabo, Epalinges, Switzerland) connected to an unidirectional valve (Hans Rudolph, Kansas City, MO) and to a differential pressure transducer (Validyne MP45 6 225 cm H2O) | - Patient position: supine position (30 ˚- 45˚).  - Timepoint of measurement: during a 2h T-piece trial.  - Maneuver: selective occlusions in the inspiratory limb by manually inflating a latex balloon during expiration.  - Number of measurements: average of five measurements obtained at random during a 60- to 90s periods. | Sustained SB >48h after extubation |
| ^a^ : Absence of weaning failure was considered as weaning success. *: Confusion matrix was directly extracted from the data reported in the published study instead of calculated based on the sensitivity, specificity and prevalence. †: Threshold corresponding to the median threshold of the other included studies reporting on the same assessment and target condition. This threshold was used to extract the confusion matrix after obtainment of the raw data from the authors when not retrievable from the papers.  **^b^:** Weaning definitions: Four studies did not report the definition of weaning success or failure, while three studies defined weaning success as patients weaned successfully after the first SBT. Eighty-one studies defined weaning success as either a pre-defined period of spontaneous breathing, not being re-intubated or not requiring any mechanical ventilation after extubation. This period was mostly defined as 48h (n=66 studies), as 24h in 6 studies, between 3 to 7 days in 9 studies and one study did not specify. Sixteen studies mentioned that the use of NIV was not allowed following extubation.  *Abbreviations:* ARF: acute respiratory failure, CPAP: continuous positive airway pressure, FRC: functional residual capacity, ICU: general intensive care unit, MICU: medical intensive care unit, MV: mechanically ventilated/mechanical ventilation, NIV: non-invasive ventilation, NIPPV: non-invasive positive pressure ventilation, NR: not reported, RV: residual volume, SB: spontaneous breathing, SBT: spontaneous breathing trial, SICU: surgical intensive care unit, PEEP: positive end-expiratory pressure, PS: pressure support, COPD: chronic obstructive pulmonary disease, CCU: coronary care unit, EICU: emergency intensive care unit, RICU: respiratory intensive care unit, NICU: neurointensive care unit. AECOPD: acute exacerbation chronic obstructive pulmonary disease, PSV: pressure support ventilation, TLC: total lung capacity. | | | | | | | |

| Table S4. Characteristics table of studies investigating predictive accuracy of the assessment methods not included in the meta-analyses | | | | | | | |
| --- | --- | --- | --- | --- | --- | --- | --- |
| **Maximal expiratory pressure, PEmax** | | | | | | | |
| **Study** | **Country** | **Setting and population** | **Sample size (n)** | **Threshold** | **Equipment used** | **Assessment protocol** | **Definition of weaning success** |
| De Jonghe 2007^[13]^* | France | MICU, SICU,  MV patients | 79 | 30 cmH_2_O | NR | - Patient position: supine position (45°).  - Timepoint of measurement: first day of return to normal consciousness.  - Maneuver: PEmax was measured after a forced inspiration against a manual occlusion of the respiratory circuit and held for ≥ 1s.  - Lung volume from which the effort was performed: TLC  - Number of breathing efforts/tests performed: three tests were performed, selecting the highest value. | No reintubation > 48 h after extubation^a^. |
| Lim 2015^[18]^ | Taiwan | ICU and respiratory care center, MV patients | 86 | 31 cmH_2_O | - Manometer (Boehringer Laboratories, Norristown, PA, USA)  - unidirectional valve to the other end. | - Patient position: supine position (30° - 45°).  - Timepoint of measurement: after conversion of the endotracheal tube to a tracheostomy.  - Maneuver: described by Truwit, Marini 1992^[19]^.  - Lung volume from which the effort was performed: NR  - Number of breathing efforts/tests performed: three tests were performed, selecting the highest value. | Liberation from MV support for at least 5 consecutive days. |
| **Electrical activity of diaphragm, EAdi** | | | | | | | |
| **Study** | **Country** | **Setting and population** | **Sample size (n)** | **Threshold** | **Equipment used** | **Assessment protocol** | **Definition of weaning success** |
| Liu 2012^[94]^ | China | ICU, MV patients | 52 | 14.23 μV | - Nasogastric tube capable of measuring EAdi (Maquet) | - Patient position: NR  - Timing of EAdi: during SBT, CPAP 5cmH_2_0.  - Maneuver: NR  - Number of measurements: average value of five inspirations. | Patients who were extubated from a completed SBT and remained extubated for > 48 h^a^ |
| Muttini 2015^[95]^ | Italy | ICU, MV patients | 18 | 14.90 μV | - Nasogastric tube capable of measuring EAdi (Maquet) | - Patient position: NR  - Timing of EAdi: during 15 min of CPAP trial.  - Maneuver: NR  - Number of measurements: NR | Patients able to complete both CPAP trials without developing fatigue, or failure if signs of fatigue were observed at any time during the protocol |
| **Phrenic nerve stimulation, Ptr,stim** | | | | | | | |
| **Study** | **Country** | **Setting and population** | **Sample size (n)** | **Threshold** | **Equipment used** | **Assessment protocol** | **Definition of weaning success** |
| Dres 2019^[96]^* | France | MICU, MV (intubated) patients | 116 | 7 cmH_2_O | - Two figure-of-eight coils connected to a pair of Magstim® 200 stimulators (The Magstim Company, Dyfed, UK) | - Patient position: supine position (45°).  - Timepoint of measurement: before SBT.  - Maneuver: endotracheal tube was occluded, and bilateral anterolateral magnetic stimulation was performed. at the proximal end of the endotracheal tube. Stimulations were delivered at the maximum intensity allowed by the stimulator.  - Site of stimulation: posterior to the SCM at the level of the cricoid cartilage.  - Number of stimulations: three times. | Not failing the SBT or not requiring reintubation or any form of MV support (including NIV for post-extubation ARF, but not prophylactic NIV) during the 48h following extubation^a^. |
| Qing 2018^[97]^ | China | ICU, MV patients | 62 | 8.75 cmH_2_O | - 2-way non-rebreathing automatic trigger device system, with the MV connected to the pipeline. | - Patient position: supine position (45°).  - Timepoint of measurement: NR  - Maneuver: the inspired air was blown out of system from the unidirectional valve. A trigger given by the magnetic stimulator was released to stimulate the phrenic nerve.  **-** Site of stimulation: NR  - Number of stimulations: NR | No need to re-intubate within 48h after extubation. |
| **Transdiaphragmatic pressure, Pdi** | | | | | | | |
| **Study** | **Country** | **Setting and population** | **Sample size (n)** | **Threshold** | **Equipment used** | **Assessment protocol** | **Definition of weaning success** |
| Castro 2012^[98]^* | Brazil | ICU, MV patients with stroke | 20 | 40% Pdi/Pdimax | - A gastroesophageal balloon  - a pneumotachograph | - Patient position: NR.  - Timepoint of measurement: before extubation, 5 min after and 1h after.  - Maneuver: measurements during calm breathing and during a maneuver of maximal inspiration.  -Determination of the correct position of the gastroesophageal catheter: with an occlusion test and when values reached were within reference values.  - Number of breathing efforts/tests performed: NR | No decrease in the level of consciousness, respiratory discomfort and/or hypoxemia during a period of up to 30 min after extubation^a^. |
| **Parasternal intercostal thickening fraction** | | | | | | | |
| **Study** | **Country** | **Setting and population** | **Sample size (n)** | **Threshold** | **Equipment used** | **Assessment protocol** | **Definition of weaning success** |
| Dres 2021^[76]^ | France, Canada | MICU, SICU, MV patients | 122 | 8.6% | - Sparq  ultrasound system (Philips Healthcare, USA)  - Sonosite (Fujifilm Sonosite, Bothell, WA, USA) | - Patient position: NR  - Timepoint of measurement: early after extubation.  - Maneuver: The thicknesses were measured on frozen images at end-expiration and at peak inspiration while patients were breathing spontaneously without noninvasive ventilation or high-flow oxygen therapy. The probe was placed at the level of the second right intercostal space. The second right parasternal intercostal muscle was identified as a three-layered biconcave structure: two linear hyperechoic membranes running, respectively, from the anterior and posterior aspects of the adjoining ribs, and a medial portion with muscle echotexture.  - Mode of US: M-mode.  - Type of probe: 10- to 15-MHz linear probe.  - Number of measurements: average of at least three measurements. | No need for reintubation and no death within 7 days after extubation^a^. |
| Xu 2023^[99]^ | China | ICU, MV patients | 83 | 7.6 % | - Vivid7 and VividS6 (GE, Chicago, USA) | - Patient position: supine (30°-45°) position.  - Timepoint of measurement: during SBT or before reconnection to MV.  - Maneuver: the probe was placed perpendicular to the anterior thoracic surface in the sagittal plane, at the level of the right second intercostal space, approximately 2 to 3 cm lateral to the sternal border, with a window to view the second and third ribs.  - Mode of US: M-mode  - Type of probe: 4-13 MHz linear probe (Vivid6) and 4-11 MHz linear probe (Vivid7).  - Number of measurements: average of three measurements. | No reintubation within 48h after extubation and no death within 7 days after extubation^a^. |
| **Thickness of rectus abdominis (RA)** | | | | | | | |
| **Study** | **Country** | **Setting and population** | **Sample size (n)** | **Threshold** | **Equipment used** | **Assessment protocol** | **Definition of weaning success** |
| Amara 2022^[33]^ | India | ICU, MV patients ready to wean | 81 | 6.38 mm | - Philips CX 50 (Philips Healthcare, 3000 Minuteman Road, Andover, USA) | - Patient position: NR  - Timepoint of measurement: on the day of the first SBT.  - Maneuver: the probe was placed horizontally about 2-3 cm above the umbilicus and 2-3 cm away from the midline.  - Mode of US: NR.  - Type of probe: linear probe.  - Number of measurements: NR | Simple weaning: Initiation of weaning to successful extubation after first SBT |
| **Thickness of internal oblique (IO)** | | | | | | | |
| **Study** | **Country** | **Setting and population** | **Sample size (n)** | **Threshold** | **Equipment used** | **Assessment protocol** | **Definition of weaning success** |
| Amara 2022^[33]^ | India | ICU, MV patients ready to wean | 81 | 4.92 mm | - Philips CX 50 (Philips Healthcare, 3000 Minuteman Road, Andover, USA) | - Patient position: NR  - Timepoint of measurement: on the day of the first SBT.  - Maneuver: the probe was placed horizontally at the anterior axillary line, approximately at the mid-point of the right costal margin and the iliac crest. - Mode of US: NR.  - Type of probe: linear probe.  - Number of measurements: NR | Simple weaning: Initiation of weaning to successful extubation after first SBT |
| **Thickness of external oblique (EO)** | | | | | | | |
| **Study** | **Country** | **Setting and population** | **Sample size (n)** | **Threshold** | **Equipment used** | **Assessment protocol** | **Definition of weaning success** |
| Amara 2022^[33]^ | India | ICU, MV patients ready to wean | 81 | 3.15 mm | - Philips CX 50 (Philips Healthcare, 3000 Minuteman Road, Andover, USA) | - Patient position: NR  - Timepoint of measurement: on the day of the first SBT.  - Maneuver: the probe was placed horizontally at the anterior axillary line, approximately at the mid-point of the right costal margin and the iliac crest.  - Mode of US: NR.  - Type of probe: linear probe.  - Number of measurements: NR | Simple weaning: Initiation of weaning to successful extubation after first SBT |
| **Thickness of transversus abdominis (TA)** | | | | | | | |
| **Study** | **Country** | **Setting and population** | **Sample size (n)** | **Threshold** | **Equipment used** | **Assessment protocol** | **Definition of weaning success** |
| Amara 2022^[33]^ | India | ICU, MV patients ready to wean | 81 | 2.53 mm | - Philips CX 50 (Philips Healthcare, 3000 Minuteman Road, Andover, USA) | - Patient position: NR  - Timepoint of measurement: on the day of the first SBT  - Maneuver: the probe was placed horizontally at the anterior axillary line, approximately at the mid-point of the right costal margin and the iliac crest.  - Mode of US: NR.  - Type of probe: linear probe.  - Number of measurements: NR | Simple weaning: Initiation of weaning to successful extubation after first SBT |
| ^a^: Absence of SBT or weaning failure was considered as SBT or weaning success. *: Confusion matrix was directly extracted from the data reported in the published study instead of calculated based on the sensitivity, specificity and prevalence. *Abbreviations:* CPAP: continuous positive airway pressure, ICU: general intensive care unit, MV: mechanically ventilated/mechanical ventilation, NR: not reported, SBT: spontaneous breathing trial,  MICU: medical intensive care unit, ARF: acute respiratory failure, NIV: non-invasive ventilation, sSICU: surgical intensive care unit, Ptr, stim: phrenic nerve stimulation (endotracheal tube pressure), SCM: sternocleidomastoid muscle(s), Pdimax: maximal transdiaphragmatic pressure. | | | | | | | |

# Figure S1. Accuracy of respiratory muscle assessment methods for predicting weaning success


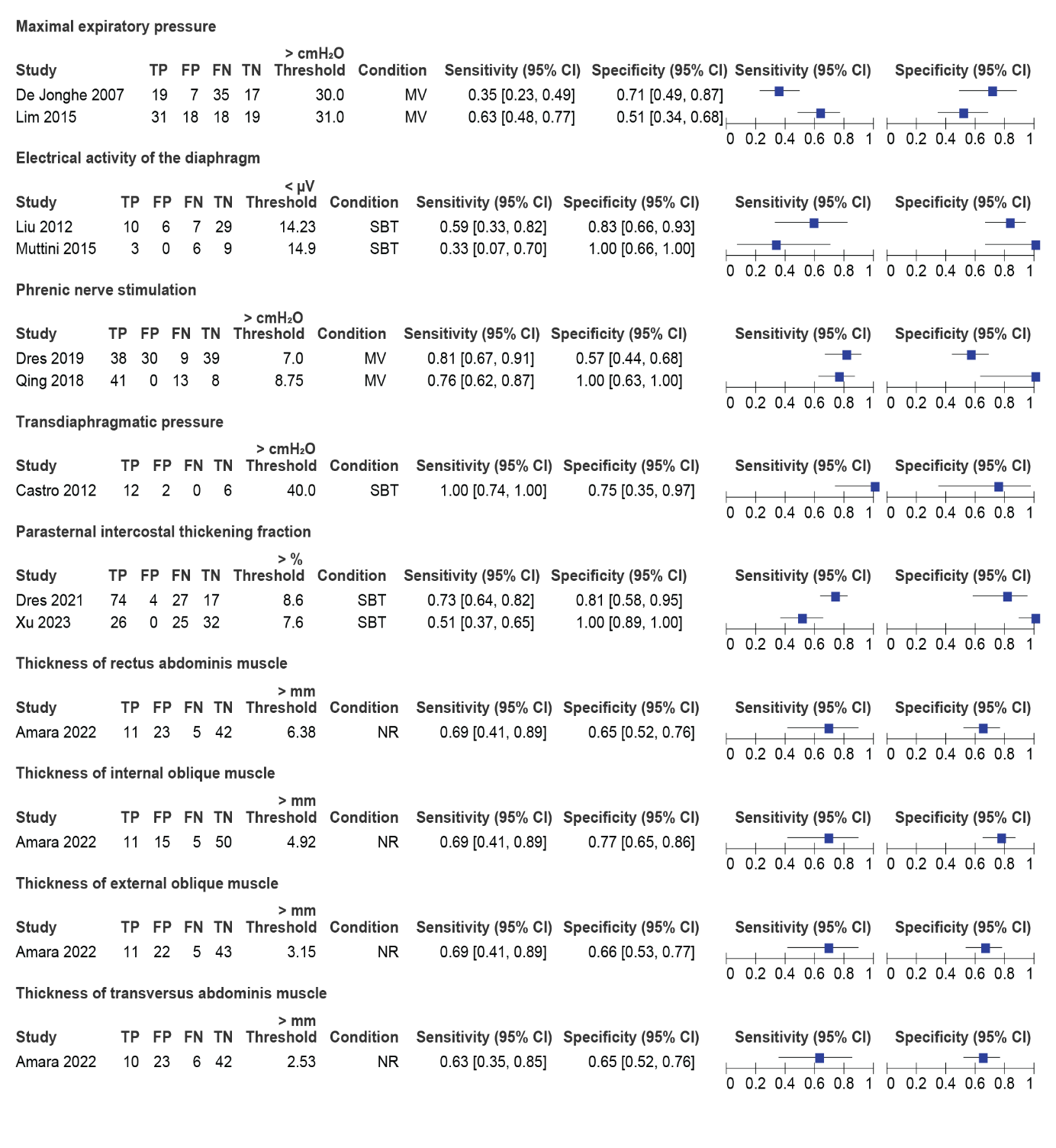


Figure depicts the individual sensitivity and specificity of each study on the respiratory assessment method of interest for predicting weaning success. Condition: Indicates whether the assessment was performed while the patients was mechanically ventilated (MV) or during spontaneous breathing/ spontaneous breathing trial (SBT). If no or insuffient data was provided on the timepint it is marked as not reported (NR). Abbreviations: TP: true positive, FP: false positive, FN: false negative, TN: true negative, SBT: spontaneous breathing trial, MV: mechanical ventilation, NR: not reported, CI: confidence interval.

# Figure S2. Risk of bias and applicability concerns for weaning success per assessment method, part 1 of 3


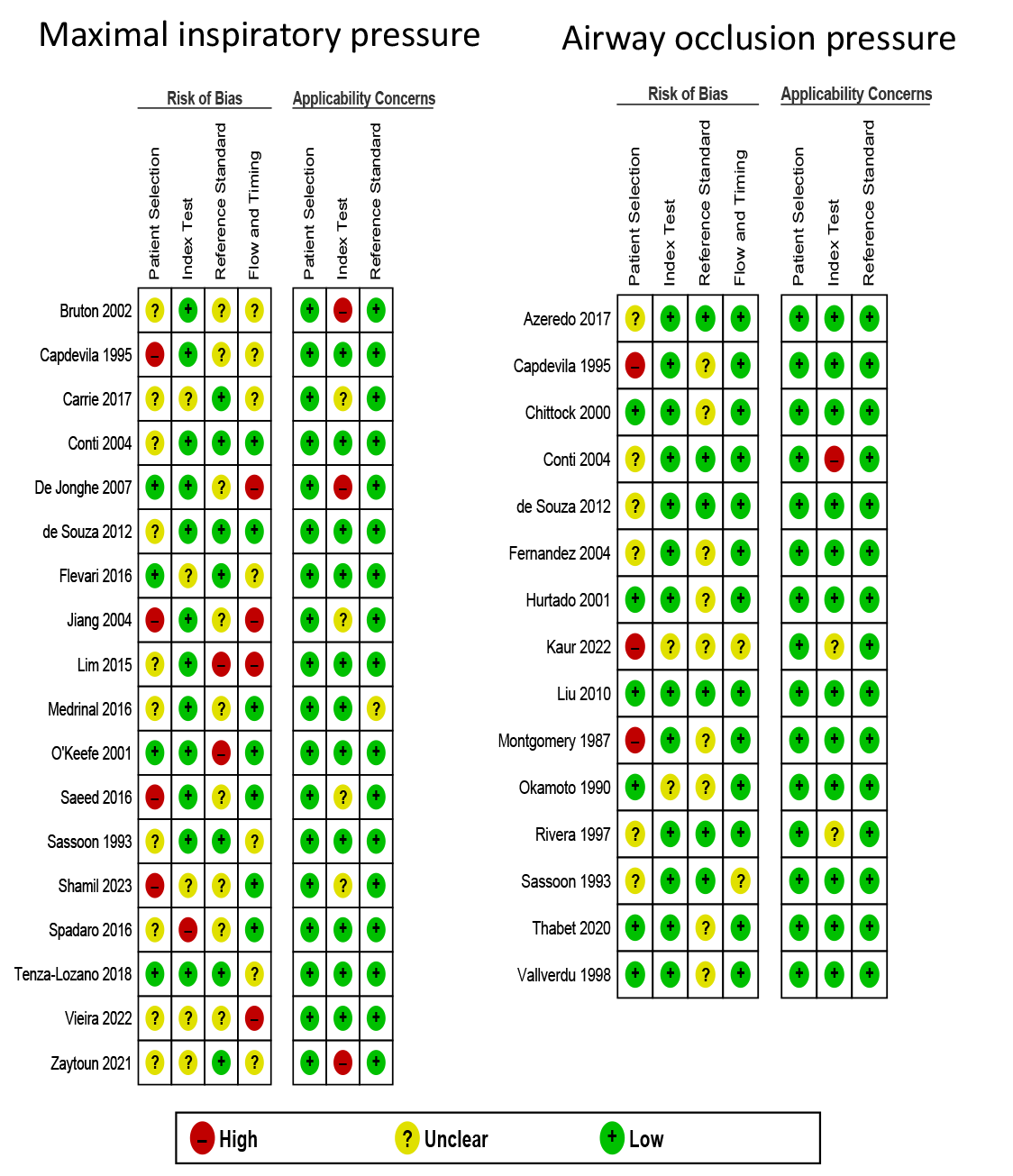


Risk of bias evaluated with the QUADAS 2 tool of all studies evaluating a respiratory muscle assessment method of interest for predicting weaning success. Figures show the identified risk of bias for each domain of risk of bias and of applicability concerns for each individual study.

Figure S3. Risk of bias and applicability concerns for weaning success per assessment method, part 2 of 3


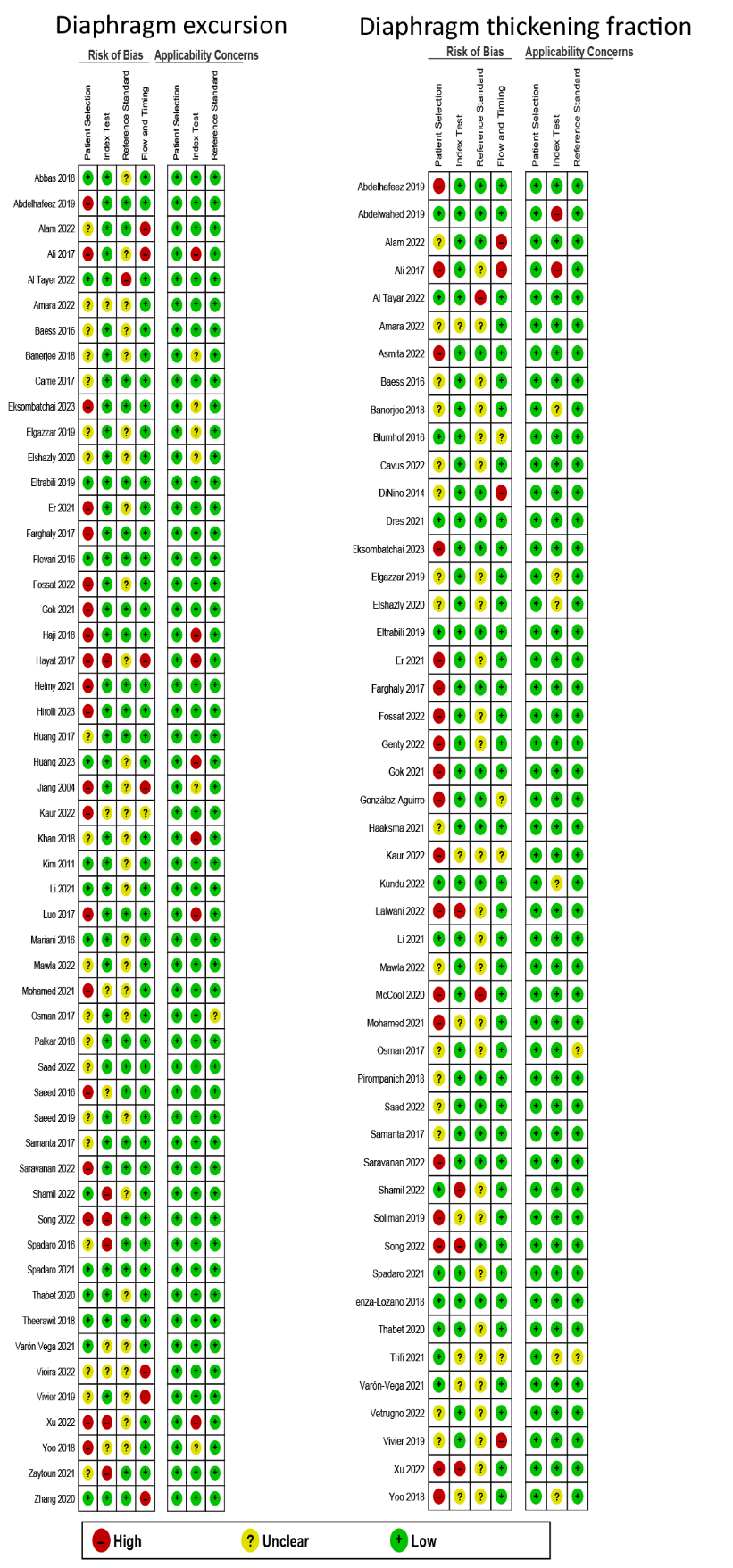


Risk of bias evaluated with the QUADAS 2 tool of all studies evaluating a respiratory muscle assessment method of interest for predicting weaning success. Figures show the identified risk of bias for each domain of risk of bias and of applicability concerns for each individual study

# Figure S4. Risk of bias and applicability concerns for weaning success per assessment method, part 3 of 3


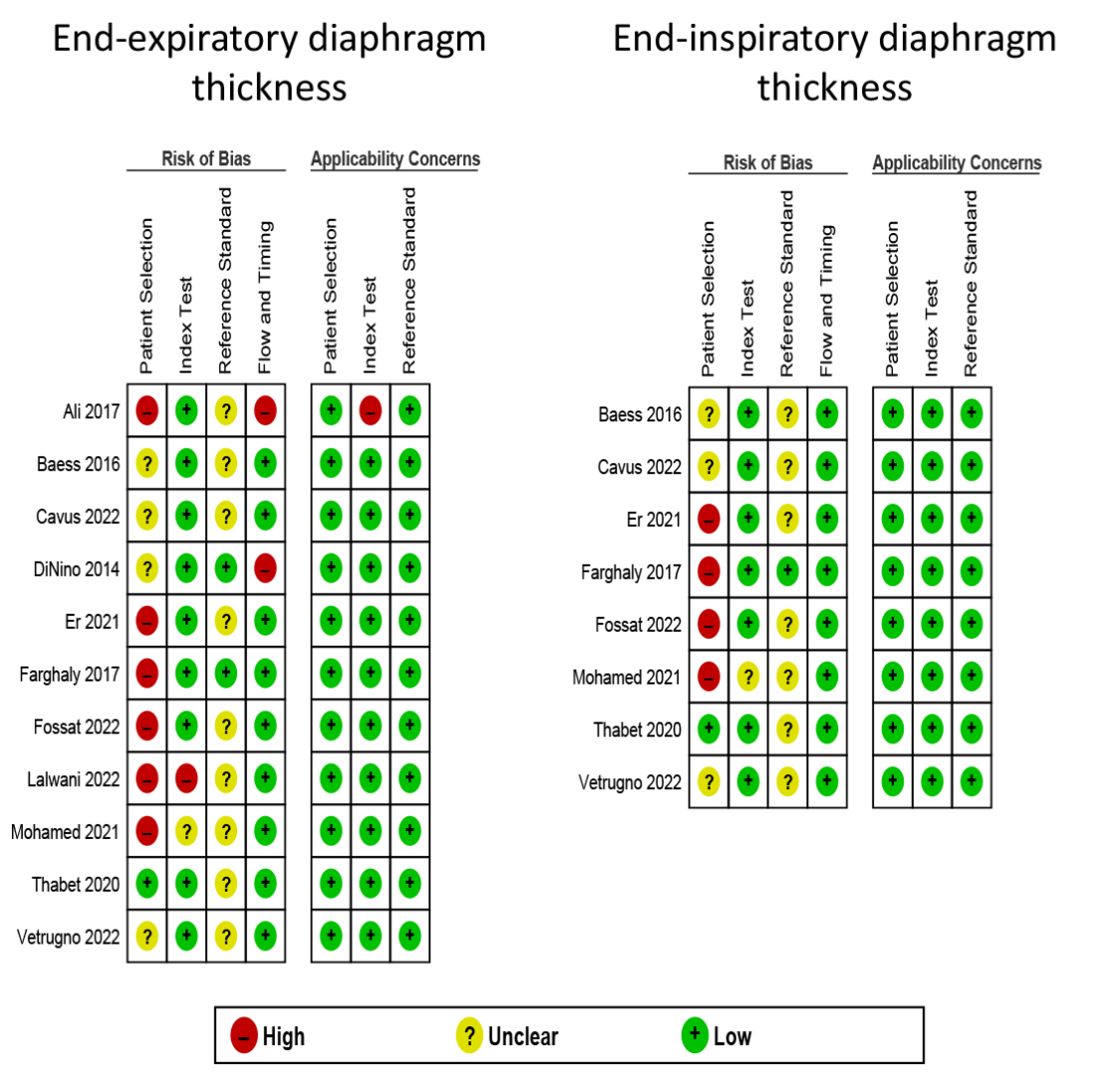


Risk of bias evaluated with the QUADAS 2 tool of all studies evaluating a respiratory muscle assessment method of interest for predicting weaning success. Figures show the identified risk of bias for each domain of risk of bias and of applicability concerns for each individual study

# Figure S5. Estimated summary receiver operating characteristic (SROC) curves to predict WS


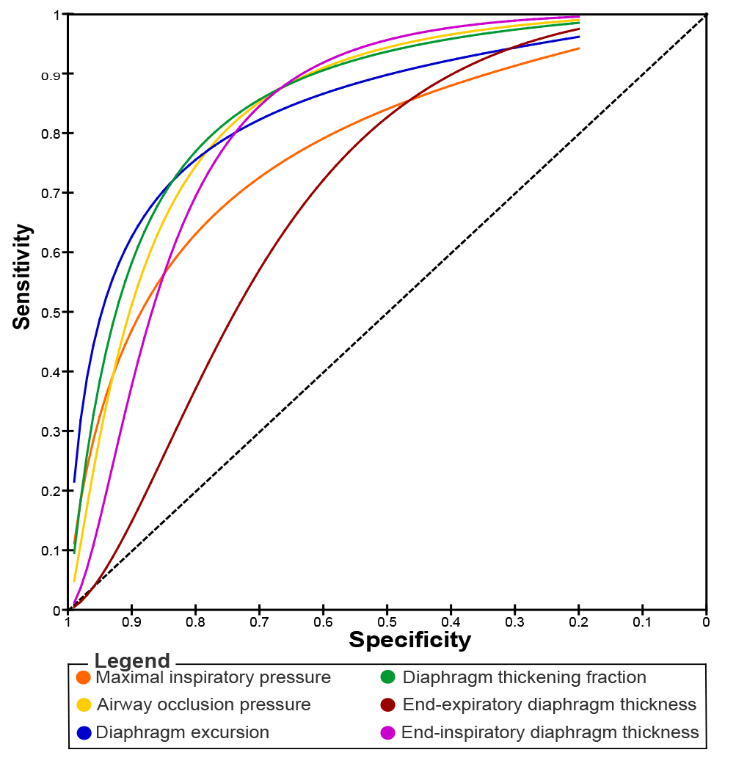


Figure depicts the summary receiver operating characteristic curves estimated with HSROC model for each respiratory muscle assessment method for which at least 4 studies reporting on test accuracy to predict weaning success was found. Single study points were not plotted for clarity of the image.

# Figure S6. Estimated SROC curves to predict WS after exclusion of studies with high risk of bias


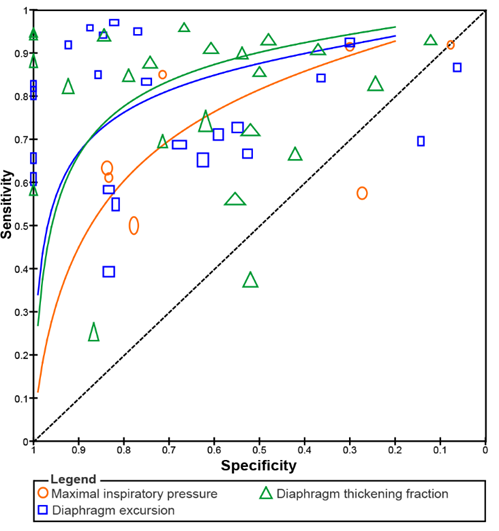


Figure depicts the summary receiver operating characteristic curves estimated with HSROC model for PImax, DTF to predict weaning success after exclusion of studies with high RoB. Different plotting symbols and colors represent the results extracted from individual studies for PImax, DE and DTF. Single study points are scaled according to inverse standard error. Abbreviations: WS: weaning success, RoB: risk of bias.

# Figure S7. Estimated SROC curves to predict WS considering direct comparative studies


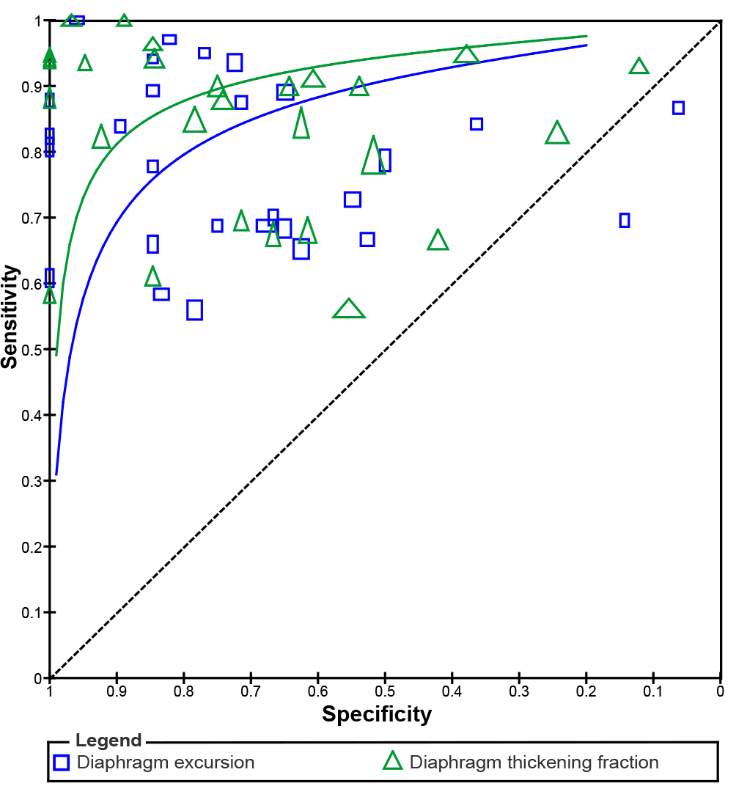


Figure shows the estimated SROC curves for the pair of assessment methods DE and DTF based on direct comparative studies. Different plotting symbols and colors represent the results extracted from individual studies for DE and DTF. Single study points are scaled according to inverse standard error. Abbreviations: WS: weaning success.

# Figure S8. Estimated SROC curves to predict weaning success based on direct comparative studies


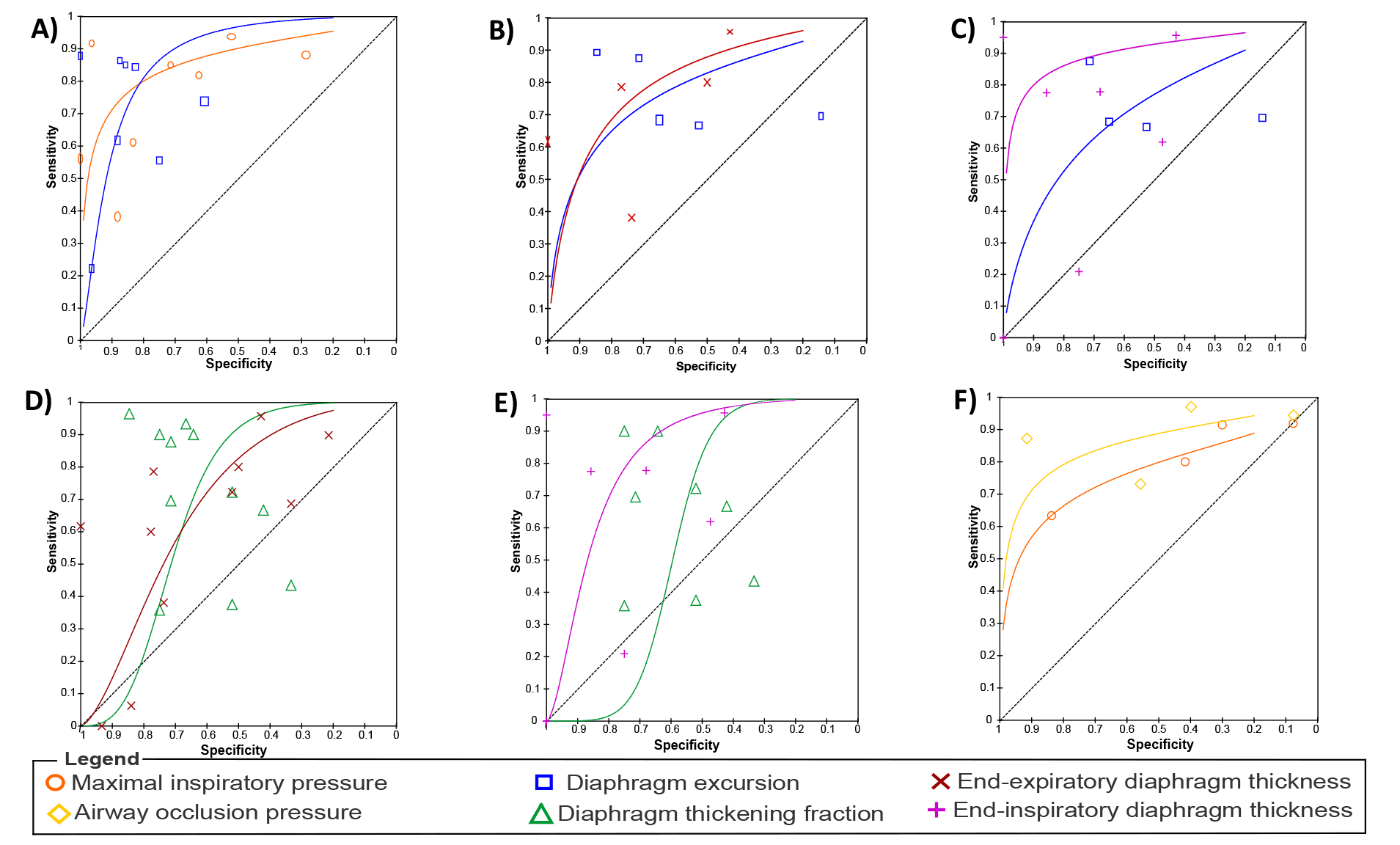


Figure depicts the summary receiver operating characteristic curves estimated with HSROC model based on direct comparative studies for the following pairs of assessment methods: **A)** DE and PImax, **B)** DE and Tdiee, **C)** DE and Tdiei, **D)** Tdiee and DTF, **E)** Tdi_ei_ and DTF, **F)** P0.1 and PImax. Direct comparative studies are studies which compared each pair of assessments on the same patients. Different plotting symbols and colours represent the results extracted from individual studies for the different respiratory muscle assessments. Single study points are scaled according to inverse standard error.

| Table S5. Within-assessment SROC curve comparisons with timepoint as potential source heterogeneity. | | | | | | | | | |
| --- | --- | --- | --- | --- | --- | --- | --- | --- | --- |
| **Assessment** | **Timepoint** | **Studies n** | **Patients n** | **-2Log Likelihood value**  **of the fitted models** | | | **Likelihood ratio assessments** | | |
|  |  |  |  | **Varied**  **model** | **Fixed shape model** | **Fixed accuracy model** | **Model comparison** | **Chi-square (df=1)** | **p-value** |
| **DTF** | During MV | 7 | 409 | 529.7 | 529.7 | 531.1 | Fixed shape vs. Varied | 0 | 1 |
|  | During SBT | 38 | 2769 |  |  |  | Fixed accuracy vs. Fixed shape | 1.4 | 0.24 |
| **PImax*** | During MV | 6 | 542 | 145.9 | 148.7 | 148.8 | Fixed shape vs. Varied | 2.8 | 0.09 |
|  | During SBT | 7 | 345 |  |  |  | Fixed accuracy vs. Fixed shape | 0.1 | 0.75 |
| **P0.1** | During MV | 4 | 670 | 161.5 | 161.8 | 162.9 | Fixed shape vs. Varied | 0.3 | 0.58 |
|  | During SBT | 10 | 510 |  |  |  | Fixed accuracy vs. Fixed shape | 1.1 | 0.29 |
| **DE** | During MV | 4 | 236 | 577.7 | 580.8 | 580.8 | Fixed shape vs. Varied | 3.1 | 0.08 |
|  | During SBT | 47 | 3191 |  |  |  | Fixed accuracy vs. Fixed shape | 0 | 1 |
| **Tdiee** | During MV | 4 | 217 | 121.9 | 127.9 | 128.3 | Fixed shape vs. Varied | 8 | **0.005** |
|  | During SBT | 7 | 400 |  |  |  | Fixed accuracy vs. Fixed shape | ¥ | ¥ |
| To compare SROC curves, the following HSROC models were compared: Model 1 (“Varied”) which includes covariates to allow accuracy, threshold and shape to vary by assessment; Model 2 (“Fixed shape”) from which the covariate term for shape was removed, to assume that the SROC curves under comparison have the same shapes; Model 3 (“Fixed accuracy”) from which also the covariate term for accuracy was removed to assume that the SROC curves under comparison have the same accuracy. A significant p-value for Model 2 vs. 1 denotes that the shapes of the SROC curves under comparison are different; a significant p-value for Model 3 vs. 2 comparison denotes that the overall accuracies of the assessments under comparison are different. *the measurements were performed either during a brief disconnection from MV or during an SBT. ¥= in case of a significant p-value for Model 2 vs. 1, which denotes that the shapes of the SROC curves under comparison are different, further comparisons of Model 3 vs. 2 was not performed. Significance level: p<0.05 (highlighted in bold). The condition of assessment could not be evaluated as potential sources of heterogeneity for Tdi_ei_ because less than 4 studies performed the assessment during mechanical ventilation. Abbreviations: DTF: diaphragm thickening fraction, PImax: maximal inspiratory pressure, P0.1: airway occlusion pressure, MV: mechanical ventilation, SBT: spontaneous breathing trial, df: degrees of freedom which are equal to the difference in the number of parameters between the models that are compared. | | | | | | | | | |

| Table S6. Within-assessment SROC curve comparisons with threshold as potential source of heterogeneity. | | | | | | | | | | | |  |
| --- | --- | --- | --- | --- | --- | --- | --- | --- | --- | --- | --- | --- |
| **Assessment** | **Threshold** | **Studies n** | **Patients n** | **-2Log Likelihood value**  **of the fitted models** | | | | | **Likelihood ratio assessments** | | | |
|  |  |  |  | **Varied model** | **Fixed shape model** | | **Fixed accuracy model** | | **Model comparison** | **Chi-square (df=1)** | **p-value** | |
| **DE** | Low:  9.1 - 11.9 mm | 30 | 2226 | 605.3 | 607.8 | | 607.9 | | Fixed shape vs. Varied | 2.5  0.1 | 0.11  0.75 | |
|  | High:  12.0 - 61 mm | 23 | 1412 |  |  |  |  |  | Fixed accuracy vs. Fixed shape |  |  |  |
| **DTF** | Low:  13.5 - 29.5 % | 20 | 1416 | 570 | 571.7 | | 572.1 | | Fixed shape vs. Varied | 1.7 | 0.58 | |
|  | High:  30.0 - 50 % | 28 | 2116 |  |  |  |  |  | Fixed accuracy vs. Fixed shape | 0.4 | 0.65 | |
| **PImax** | Low:  16 - 29.5 cmH_2_O | 6 | 226 | 195.6 | 195.7 | | 197.5 | | Fixed shape vs. Varied | 0.1 | 0.75 | |
|  | High:  30 - 50 cmH2O | 12 | 881 |  |  |  |  |  | Fixed accuracy vs. Fixed shape | 1.8 | 0.18 | |
| **P0.1** | Low:  2.3 - 3.5 cmH2O | 6 | 583 | 166.1 | 168.9 | | 169.7 | | Fixed shape vs. Varied | 2.8 | 0.09 | |
|  | High:  3.6 - 7.5 mm | 9 | 642 |  |  |  |  |  | Fixed accuracy vs. Fixed shape | 0.8 | 0.37 | |
| **Tdi_ee_** | Low:  1.7 - 2.89 mm  High:  2.9 – 15.5 mm | 4 | 211 | 130 | | 131.1 | | 131.2 | Fixed shape vs. Varied | 1.1 | 0.29 | |
|  |  | 7 | 406 |  |  |  |  |  | Fixed accuracy vs. Fixed shape | 0.1 | 0.75 | |
| Thresholds were categorized as low or high depending on the median threshold of all included studies for the respective assessment method. A threshold was identified as low when the value was between the lowest observed threshold and the median and as a high threshold when the value was between the median and the highest observed threshold. To compare SROC curves, the following HSROC models were compared: Model 1 (“Varied”) which includes covariates to allow accuracy, threshold and shape to vary by assessment; Model 2 (“Fixed shape”) from which the covariate term for shape was removed, to assume that the SROC curves under comparison have the same shapes; Model 3 (“Fixed accuracy”) from which also the covariate term for accuracy was removed to assume that the SROC curves under comparison have the same accuracy. A significant p-value for Model 2 vs. 1 denotes that the shapes of the SROC curves under comparison are different; a significant p-value for Model 3 vs. 2 comparison denotes that the overall accuracies of the assessments under comparison are different. Significance level: p<0.05. The chosen threshold could not be evaluated as potential sources of heterogeneity for Tdi_ei_ because less than 4 studies used a low threshold. Abbreviations: DE: diaphragm excursion, DTF: diaphragm thickening fraction, PImax: maximal inspiratory pressure, P0.1: airway occlusion pressure, df: degrees of freedom which are equal to the difference in the number of parameters between the models that are compared. | | | | | | | | | | | |  |

| Table S7. Estimated HSROC parameters after exclusion of studies with high RoB | | | | | | | | | | |
| --- | --- | --- | --- | --- | --- | --- | --- | --- | --- | --- |
|  | **HSROC parameters** | | | | | | | | | |
|  | **alpha** | | **theta** | | **beta** | | **s2ua** | | **s2ut** | |
| **Assessment** | Estimate | SE | Estimate | SE | Estimate | SE | Estimate | SE | Estimate | SE |
| **PImax** | 1.6931 | 0.2879 | 0.5499 | 0.4825 | 0.2640 | 0.2856 | 0 | 0 | 1.4626 | 0.8861 |
| **DTF** | 2.6464 | 0.4315 | 0.5551 | 0.2537 | 0.3569 | 0.2745 | 2.8245 | 1.1125 | 0.7944 | 0.2941 |
| **DE** | 2.5887 | 0.3881 | 0.5010 | 0.2501 | 0.5636 | 0.2983 | 2.0697 | 0.8742 | 0.6547 | 0.2497 |
| **P0.1** | 2.3730 | 0.6957 | -0.09875 | 0.2201 | -0.9426 | 0.4527 | 3.9498 | 2.8852 | 0.04348 | 0.08683 |
| **Tdi_ee_** | 0.9794 | 0.4279 | -0.3810 | 0.6730 | -0.9535 | 0.4106 | 0 | 0 | 1.5965 | 1.3226 |
| **Tdi_ei_** | 1.3876 | 0.5019 | -0.9928 | 1.4547 | -0.6174 | 0.5480 | 0.1918 | 0.5711 | 7.1417 | 7.1165 |
| The estimates of the HSROC parameters can be entered into RevMan to plot the corresponding SROC curve. Estimates parameters represents the estimate for the mean for accuracy (alpha), for the mean for threshold (theta), for the shape parameter (beta), for the variance of the random effects for accuracy (s2ua) and for the variance of the random effects for accuracy for threshold (s2ut)^[100]^. *Abbreviations:* DE: diaphragm excursion; DTF : diaphragm thickening fraction; PImax: maximal inspiratory pressure; P0.1: airway occlusion pressure; Tdi_ee_: diaphragm thickness at end-expiration; SE: standard error, RoB: risk of bias. | | | | | | | | | | |

| Table S8. Comparisons of SROC curves to predict WS after excluding studies with high risk of bias. | | | | | | | | |  |
| --- | --- | --- | --- | --- | --- | --- | --- | --- | --- |
| **Assessment comparison** | **Studies n** | **Patients n** | **-2Log Likelihood value**  **of the fitted models** | | | **Likelihood ratio assessments** | | | **RDOR**  **(95%CI)** |
|  |  |  | **Varied model (V)** | **Fixed shape model (FS)** | **Fixed accuracy model (FA)** | **Model comparison** | **Chi-square (df=1)** | **p-value** |  |
| **DE**  **vs.**  **PImax** | 23 | 1323 | 335.4 | 335.6 | 336.9 | FS vs. V | 0.2 | 0.65 | 1.63  (0.48-5.55) |
|  | 7 | 441 |  |  |  | FA vs. FS | 1.3 | 0.25 |  |
| **DTF**  **vs.**  **PImax** | 23 | 1430 | 347.5 | 347.5 | 349.6 | FS vs. V | 0 | 1 | 2.59  (0.73-9.24) |
|  | 7 | 441 |  |  |  | FA vs. FS | 2.1 | 0.15 |  |
| **DTF**  **vs.**  **DE** | 23 | 1323 | 536 | 536 | 542.6 | FS vs. V | 0 | 1 | 1.82  (1.10-3.01) |
|  | 23 | 1430 |  |  |  | FA vs. FS | 6.6 | **0.01** |  |
| To compare SROC curves, the following HSROC models were compared: Model 1 (“Varied”, V) which includes covariates to allow accuracy, threshold and shape to vary by assessment; Model 2 (“Fixed shape”, FS) from which the covariate term for shape was removed, to assume that the SROC curves under comparison have the same shapes; Model 3 (“Fixed accuracy”, FA) from which also the covariate term for accuracy was removed to assume that the SROC curves under comparison have the same accuracy. A significant p-value for Model 2 vs. 1 denotes that the shapes of the SROC curves under comparison are different; a significant p-value for Model 3 vs. 2 comparison denotes that the overall accuracies of the assessments under comparison are different. Significance level: p<0.05 (highlighted in bold). SROC curves of P0.1, Tdi_ee_ and Tdi_ei_ could not be compared due to their asymmetrical aspect. Values of RDOR were calculated as relative diagnostic odds ratio of the first assessment compared to the second, as indicated by the order specified in the “Assessment comparison” column. A value of the RDOR higher or lower than 1 indicate that the first assessment has higher or lower accuracy than the second one. Confidence intervals of RDOR not containing 1 indicate significant higher or lower accuracy.*Abbreviations:* CI: confidence interval, DE: diaphragm excursion; DTF: diaphragm thickening fraction; PImax: maximal inspiratory pressure; RoB: risk of bias; df: degrees of freedom which are equal to the difference in the number of parameters between the models that are compared; WS: weaning success. | | | | | | | | | |

| **Table S9. SROC curves comparisons after excluding studies conducting assessments early after start of MV** | | | | | | | | |  |
| --- | --- | --- | --- | --- | --- | --- | --- | --- | --- |
| **Assessment comparison** | **Studies n** | **Patients n** | **-2Log Likelihood value of the fitted models** | | | **Likelihood ratio assessments** | | | **RDOR**  **(95%CI;**  **p-value)** |
|  |  |  | **Varied model (V)** | **Fixed shape model (FS)** | **Fixed accuracy model**  **(FA)** | **Model comparison** | **Chi-square (df=1)** | **p-value** |  |
| **DE**  **vs.**  **PImax** | 53 | 3638 | 857.9 | 858.2 | 862.3 | FS vs. V | 0.3 | 0.58 | 2.11  (1.14-3.93) |
|  | 18 | 1107 |  |  |  | FA vs. FS | 4.1 | **0.04** |  |
| **DTF**  **vs.**  **PImax** | 46 | 3403 | 775.0 | 775.3 | 788.5 | FS vs. V | 0.3 | 0.58 | 4.97  (1.81-13.69) |
|  | 18 | 1107 |  |  |  | FA vs. FS | 13.2 | **<0.001** |  |
| **DE**  **vs.**  **DTF** | 53 | 3638 | 1254.6 | 1280.6 | 1281.0 | FS vs. V | 26 | **<0.001** | ¥ |
|  | 46 | 3403 |  |  |  | FA vs. FS | ¥ | ¥ |  |
| **DE**  **vs.**  **Tdi_ee_** | 53 | 3638 | 738.6 | 740.3 | 742.7 | FS vs. V | 1.7 | 0.19 | 1.88  (0.88-3.98) |
|  | 10 | 579 |  |  |  | FA vs. FS | 2.4 | 0.12 |  |
| **DTF**  **vs.**  **Tdi_ee_** | 46 | 3403 | 718.4 | 722.8 | 725.2 | FS vs. V | 4.4 | **0.03** | ¥ |
|  | 10 | 579 |  |  |  | FA vs. FS | ¥ | ¥ |  |
| **PImax**  **vs.**  **Tdi_ee_** | 18 | 1107 | 324.3 | 325.2 | 326.0 | FS vs. V | 0.9 | 0.34 | 1.50  (0.55-4.14) |
|  | 10 | 579 |  |  |  | FA vs. FS | 0.8 | 0.37 |  |
| **PImax**  **vs.**  **P0.1** | 18 | 1107 | 380.6 | 382.5 | 383.4 | FS vs. V | 1.9 | 0.17 | 0.68  (0.30-1.54) |
|  | 15 | 1225 |  |  |  | FA vs. FS | 0.9 | 0.34 |  |
| **DE**  **vs.**  **P0.1** | 53 | 3638 | 782.9 | 787.1 | 787.1 | FS vs. V | 4.2 | **0.04** | ¥ |
|  | 15 | 1225 |  |  |  | FA vs. FS | ¥ | ¥ |  |
| **DTF**  **vs.**  **P0.1** | 46 | 3471 | 723.5 | 725.2 | 726.0 | FS vs. V | 1.7 | 0.19 | 1.57  (0.53-4.71) |
|  | 15 | 1225 |  |  |  | FA vs. FS | 0.8 | 0.37 |  |
| **P0.1**  **vs.**  **Tdi_ee_** | 15 | 1225 | 296.3 | 296.9 | 297.5 | FS vs. V | 0.6 | 0.44 | 1.87  (0.51-6.82) |
|  | 10 | 579 |  |  |  | FA vs. FS | 0.6 | 0.44 |  |
| To compare SROC curves, the following HSROC models were compared: Model 1 (“Varied”, V) which includes covariates to allow accuracy, threshold and shape to vary by assessment; Model 2 (“Fixed shape”, FS) from which the covariate term for shape was removed, to assume that the SROC curves under comparison have the same shapes; Model 3 (“Fixed accuracy”, FA) from which also the covariate term for accuracy was removed to assume that the SROC curves under comparison have the same accuracy. A significant p-value for Model 2 vs. 1 denotes that the shapes of the SROC curves under comparison are different; a significant p-value for Model 3 vs. 2 comparison denotes that the overall accuracies of the assessments under comparison are different. ¥= in case of a significant p-value for Model 2 vs. 1, which denotes that the shapes of the SROC curves under comparison are different, further comparisons of Model 3 vs. 2 was not performed. Significance level: p<0.05 (highlighted in bold). Values of RDOR were calculated as relative diagnostic odds ratio of the first assessment compared to the second, as indicated by the order specified in the “Assessment comparison” column. A value of the RDOR higher or lower than 1 indicate that the first assessment has higher or lower accuracy than the second one. Confidence intervals of RDOR not containing 1 indicate significant higher or lower accuracy. *Abbreviations:* CI: confidence interval, DE: diaphragm excursion; DTF : diaphragm thickening fraction; MV: mechanical ventilation; PImax: maximal inspiratory pressure; P0.1: airway occlusion pressure; Tdi_ee_: diaphragm thickness at end-expiration; df: degrees of freedom which are equal to the difference in the number of parameters between the models that are compared; WS: weaning success. | | | | | | | | | |

# References

1. Varon-Vega F, Hernandez A, Lopez M, Caceres E, Giraldo-Cadavid LF, Uribe-Hernandez AM, et al. Usefulness of diaphragmatic ultrasound in predicting extubation success. Med Intensiva (Engl Ed). 2021;45(4):226-33.

2. McCool FD, Oyieng'o DO, Koo P. The Utility of Diaphragm Ultrasound in Reducing Time to Extubation. Lung. 2020;198(3):499-505.

3. Farghaly S, Hasan AA. Diaphragm ultrasound as a new method to predict extubation outcome in mechanically ventilated patients. Aust Crit Care. 2017;30(1):37-43.

4. González-Aguirre JE, Rivera-Uribe CP, Rendón-Ramírez EJ, Cañamar-Lomas R, Serna-Rodríguez JA, Mercado-Longoría R. Pulmonary Ultrasound and Diaphragmatic Shortening Fraction Combined Analysis for Extubation-Failure-Prediction in Critical Care Patients. Arch Bronconeumol (Engl Ed). 2019;55(4):195-200.

5. Ali ER, Mohamad AM. Diaphragm ultrasound as a new functional and morphological index of outcome, prognosis and discontinuation from mechanical ventilation in critically ill patients and evaluating the possible protective indices against VIDD. Egypt J Chest Dis Tu. 2017;66(2):339-51.

6. Luo L, Li Y, Chen X, Sun B, Li W, Gu W, et al. Different effects of cardiac and diaphragm function assessed by ultrasound on extubation outcomes in difficult-to-wean patients: a cohort study. Bmc Pulm Med. 2017;17(1):161.

7. Bruton A. A pilot study to investigate any relationship between sustained maximal inspiratory pressure and extubation outcome. Heart Lung. 2002;31(2):141-9.

8. Capdevila XJ, Perrigault PF, Perey PJ, Roustan JP, d'Athis F. Occlusion pressure and its ratio to maximum inspiratory pressure are useful predictors for successful extubation following T-piece weaning trial. Chest. 1995;108(2):482-9.

9. Marini JJ, Smith TC, Lamb V. Estimation of inspiratory muscle strength in mechanically ventilated patients: The measurement of maximal inspiratory pressure. Journal of Critical Care. 1986;1(1):32-8.

10. Carrie C, Gisbert-Mora C, Bonnardel E, Gauche B, Biais M, Vargas F, et al. Ultrasonographic diaphragmatic excursion is inaccurate and not better than the MRC score for predicting weaning-failure in mechanically ventilated patients. Anaesth Crit Care Pain Med. 2017;36(1):9-14.

11. Conti G, Montini L, Pennisi MA, Cavaliere F, Arcangeli A, Bocci MG, et al. A prospective, blinded evaluation of indexes proposed to predict weaning from mechanical ventilation. Intensive Care Med. 2004;30(5):830-6.

12. Conti G, De Blasi R, Pelaia P, Benito S, Rocco M, Antonelli M, et al. Early prediction of successful weaning during pressure support ventilation in chronic obstructive pulmonary disease patients. Crit Care Med. 1992;20(3):366-71.

13. De Jonghe B, Bastuji-Garin S, Durand MC, Malissin I, Rodrigues P, Cerf C, et al. Respiratory weakness is associated with limb weakness and delayed weaning in critical illness. Crit Care Med. 2007;35(9):2007-15.

14. de Souza LC, da Silva CT, Jr., Almeida JR, Lugon JR. Comparison of maximal inspiratory pressure, tracheal airway occlusion pressure, and its ratio in the prediction of weaning outcome: impact of the use of a digital vacuometer and the unidirectional valve. Respir Care. 2012;57(8):1285-90.

15. Flevari A, Lignos M, Konstantonis D, Armaganidis A. Diaphragmatic ultrasonography as an adjunct predictor tool of weaning success in patients with difficult and prolonged weaning. Minerva Anestesiol. 2016;82(11):1149-57.

16. ATS/ERS Statement on respiratory muscle testing. Am J Respir Crit Care Med. 2002;166(4):518-624.

17. Jiang JR, Tsai TH, Jerng JS, Yu CJ, Wu HD, Yang PC. Ultrasonographic evaluation of liver/spleen movements and extubation outcome. Chest. 2004;126(1):179-85.

18. Lim CK, Ruan SY, Lin FC, Wu CL, Chang HT, Jerng JS, et al. Effect of Tracheostomy on Weaning Parameters in Difficult-to-Wean Mechanically Ventilated Patients: A Prospective Observational Study. PLoS One. 2015;10(9):e0138294.

19. Truwit JD, Marini JJ. Validation of a technique to assess maximal inspiratory pressure in poorly cooperative patients. Chest. 1992;102(4):1216-9.

20. Medrinal C, Prieur G, Frenoy É, Robledo Quesada A, Poncet A, Bonnevie T, et al. Respiratory weakness after mechanical ventilation is associated with one-year mortality - a prospective study. Crit Care. 2016;20(1):231.

21. O'Keefe GE, Hawkins K, Boynton J, Burns D. Indicators of fatigue and of prolonged weaning from mechanical ventilation in surgical patients. World J Surg. 2001;25(1):98-103.

22. Saeed AM, El Assal GI, Ali TM, Hendawy MM. Role of ultrasound in assessment of diaphragmatic function in chronic obstructive pulmonary disease patients during weaning from mechanical ventilation. Egypt J Bronchol. 2016;10(2):167-72.

23. Sassoon CS, Mahutte CK. Airway occlusion pressure and breathing pattern as predictors of weaning outcome. Am Rev Respir Dis. 1993;148(4 Pt 1):860-6.

24. Shamil PK, Gupta NK, Ish P, Sen MK, Kumar R, Chakrabarti S, et al. Prediction of Weaning Outcome from Mechanical Ventilation Using Diaphragmatic Rapid Shallow Breathing Index. Indian J Crit Care Med. 2022;26(9):1000-5.

25. Spadaro S, Grasso S, Mauri T, Dalla Corte F, Alvisi V, Ragazzi R, et al. Can diaphragmatic ultrasonography performed during the T-tube trial predict weaning failure? The role of diaphragmatic rapid shallow breathing index. Crit Care. 2016;20(1):305.

26. Tenza-Lozano E, Llamas-Alvarez A, Jaimez-Navarro E, Fernández-Sánchez J. Lung and diaphragm ultrasound as predictors of success in weaning from mechanical ventilation. Crit Ultrasound J. 2018;10(1):12.

27. Vieira FN, Bertazzo RB, Nascimento GC, Anderle M, Coelho AC, Chaise FO, et al. Association between rectus femoris cross-sectional area and diaphragmatic excursion with weaning of tracheostomized patients in the intensive care unit. Rev Bras Ter Intensiva. 2022;34(4):452-60.

28. Zaytoun TM, Elsayed HE, Elghazaly AM. The role of diaphragmatic rapid shallow breathing index and maximum inspiratory pressure in predicting outcome of weaning from mechanical ventilation. Egypt J Chest Dis Tu. 2021;70(4):526-33.

29. Abbas A, Embarak S, Walaa M, Lutfy SM. Role of diaphragmatic rapid shallow breathing index in predicting weaning outcome in patients with acute exacerbation of COPD. Int J Chron Obstruct Pulmon Dis. 2018;13:1655-61.

30. Abdelhafeez RM, Abumossalam AM, Arram EO, Elshafey MM, Abushehata ME. Diaphragm and weaning from mechanical ventilation: anticipation and outcome. Egypt J Bronchol. 2019;13(4):489-97.

31. Alam MJ, Roy S, Iktidar MA, Padma FK, Nipun KI, Chowdhury S, et al. Diaphragm ultrasound as a better predictor of successful extubation from mechanical ventilation than rapid shallow breathing index. Acute Crit Care. 2022;37(1):94-100.

32. Al Tayar AS, Abdelshafey EE. Diaphragm Electromyography Versus Ultrasonography in the Prediction of Mechanical Ventilation Liberation Outcome. Resp Care. 2022;67(11):1437-42.

33. Amara V, Vishwas P, Maddani SS, Natarajan S, Chaudhuri S. Evaluation of Abdominal Expiratory Muscle Thickness Pattern, Diaphragmatic Excursion, and Lung Ultrasound Score in Critically Ill Patients and Their Association with Weaning Patterns: A Prospective Observational Study. Indian J Crit Care M. 2022;26(3):307-13.

34. Baess AI, Abdallah TH, Emara DM, Hassan M. Diaphragmatic ultrasound as a predictor of successful extubation from mechanical ventilation: thickness, displacement, or both? Egypt J Bronchol. 2016;10(2):162-6.

35. Banerjee A, Mehrotra G. Comparison of Lung Ultrasound-based Weaning Indices with Rapid Shallow Breathing Index: Are They Helpful? Indian J Crit Care Med. 2018;22(6):435-40.

36. Eksombatchai D, Sukkratok C, Sutherasan Y, Junhasavasdikul D, Theerawit P. The ratio of respiratory rate to diaphragm thickening fraction for predicting extubation success. Bmc Pulm Med. 2023;23(1):109.

37. ElGazzar A, Kamel KM, Mohammad OI, Abd Elraoof B. Diaphragmatic ultrasound as a predictor for successful weaning from mechanical ventilation. The Egyptian Journal of Chest Diseases and Tuberculosis. 2019;68:585 - 9.

38. Elshazly MI, Kamel KM, Elkorashy RI, Ismail MS, Ismail JH, Assal HH. Role of Bedside Ultrasonography in Assessment of Diaphragm Function as a Predictor of Success of Weaning in Mechanically Ventilated Patients. Tuberc Respir Dis (Seoul). 2020;83(4):295-302.

39. Eltrabili HH, Hasanin AM, Soliman MS, Lotfy AM, Hamimy WI, Mukhtar AM. Evaluation of Diaphragmatic Ultrasound Indices as Predictors of Successful Liberation From Mechanical Ventilation in Subjects With Abdominal Sepsis. Respir Care. 2019;64(5):564-9.

40. Er B, Simsek M, Yildirim M, Halacli B, Ocal S, Ersoy EO, et al. Association of baseline diaphragm, rectus femoris and vastus intermedius muscle thickness with weaning from mechanical ventilation. Resp Med. 2021;185.

41. Fossat G, Daillet B, Desmalles E, Boulain T. Does diaphragm ultrasound improve the rapid shallow breathing index accuracy for predicting the success of weaning from mechanical ventilation? Aust Crit Care. 2022;35(3):233-40.

42. Zambon M, Greco M, Bocchino S, Cabrini L, Beccaria PF, Zangrillo A. Assessment of diaphragmatic dysfunction in the critically ill patient with ultrasound: a systematic review. Intensive Care Med. 2017;43(1):29-38.

43. Gok F, Mercan A, Kilicaslan A, Sarkilar G, Yosunkaya A. Diaphragm and Lung Ultrasonography During Weaning From Mechanical Ventilation in Critically Ill Patients. Cureus. 2021;13(5).

44. Haji K, Haji D, Canty DJ, Royse AG, Green C, Royse CF. The impact of heart, lung and diaphragmatic ultrasound on prediction of failed extubation from mechanical ventilation in critically ill patients: a prospective observational pilot study. Crit Ultrasound J. 2018;10(1):13.

45. Hayat A, Khan A, Khalil A, Asghar A. Diaphragmatic Excursion: Does it Predict Successful Weaning from Mechanical Ventilation? J Coll Physicians Surg Pak. 2017;27(12):743-6.

46. Helmy MA, Milad LM, Osman SH, Ali MA, Hasanin A. Diaphragmatic excursion: A possible key player for predicting successful weaning in patients with severe COVID-19. Anaesth Crit Care Pa. 2021;40(3).

47. Hirolli D, Srinivasaiah B, Muthuchellappan R, Chakrabarti D. Clinical Scoring and Ultrasound-Based Diaphragm Assessment in Predicting Extubation Failure in Neurointensive Care Unit: A Single-Center Observational Study. Neurocrit Care. 2023.

48. Huang D, Ma H, Zhong W, Wang X, Wu Y, Qin T, et al. Using M-mode ultrasonography to assess diaphragm dysfunction and predict the success of mechanical ventilation weaning in elderly patients. J Thorac Dis. 2017;9(9):3177-86.

49. Huang D, Song F, Luo B, Wang S, Qin T, Lin Z, et al. Using automatic speckle tracking imaging to measure diaphragm excursion and predict the outcome of mechanical ventilation weaning. Crit Care. 2023;27(1):18.

50. Kaur A, Sharma S, Singh VP, Krishna MR, Gautam PL, Singh G. Sonographic assessment of diaphragmatic thickening and excursion as predictors of weaning success in the intensive care unit: A prospective observational study. Indian J Anaesth. 2022;66(11):776-82.

51. Khan MT, Munawar K, Hussain SW, Qadeer A, Saeed ML, Shad ZS, et al. Comparing Ultrasound-based Diaphragmatic Excursion with Rapid Shallow Breathing Index as a Weaning Predictor. Cureus. 2018;10(12):e3710.

52. Kim WY, Suh HJ, Hong SB, Koh Y, Lim CM. Diaphragm dysfunction assessed by ultrasonography: influence on weaning from mechanical ventilation. Crit Care Med. 2011;39(12):2627-30.

53. Li SG, Chen Z, Yan WF. Application of bedside ultrasound in predicting the outcome of weaning from mechanical ventilation in elderly patients. Bmc Pulm Med. 2021;21(1).

54. Mariani LF, Bedel J, Gros A, Lerolle N, Milojevic K, Laurent V, et al. Ultrasonography for Screening and Follow-Up of Diaphragmatic Dysfunction in the ICU: A Pilot Study. J Intensive Care Med. 2016;31(5):338-43.

55. Mawla TSA, Fattah SRA, Halim AMA, Elhefeny RA. Diaphragmatic Function Assessment Using Chest Ultrasonography as a Predictor for Weaning from Mechanical Ventilation. Egypt J Crit Care Me. 2022;9(1):1-9.

56. Mohamed RSE, Mohamed ASE, Fathalah WF, Mohamed MF, Ahmed AA. The role of diaphragmatic ultrasound as a predictor of successful extubation from mechanical ventilation in respiratory intensive care unit (vol 15, 51, 2021). Egypt J Bronchol. 2021;15(1).

57. Osman A, Hashim R. Diaphragmatic and lung ultrasound application as new predictive indices for the weaning process in ICU patients. The Egyptian Journal of Radiology and Nuclear Medicine. 2017;48.

58. Palkar A, Mayo P, Singh K, Koenig S, Narasimhan M, Singh A, et al. Serial Diaphragm Ultrasonography to Predict Successful Discontinuation of Mechanical Ventilation. Lung. 2018;196(3):363-8.

59. Saad MA, Nashed SW, El-Shaer AN, Elagamy AE, El Derh MS. Ultrasound-assessed diaphragmatic dysfunction as a predictor of weaning outcome in mechanically ventilated patients with sepsis in intensive care unit. Ain Shams J Anesthes. 2022;14(1).

60. Saeed AM, El Maraghy AA, Raafat RH, Abd Elsamad AM. Assessment of diaphragmatic mobility by chest ultrasound in patients with chronic obstructive pulmonary disease on different modes of mechanical ventilation. Egypt J Bronchol. 2019;13(2):184-90.

61. Samanta S, Singh RK, Baronia AK, Poddar B, Azim A, Gurjar M. Diaphragm thickening fraction to predict weaning-a prospective exploratory study. J Intensive Care. 2017;5:62.

62. Saravanan R, Nivedita K, Karthik K, Venkatraman R. Role of diaphragm ultrasound in weaning mechanically ventilated patients: A prospective observational study. Indian J Anaesth. 2022;66(8):591-8.

63. Song J, Qian Z, Zhang H, Wang M, Yu Y, Ye C, et al. Diaphragmatic ultrasonography-based rapid shallow breathing index for predicting weaning outcome during a pressure support ventilation spontaneous breathing trial. Bmc Pulm Med. 2022;22(1):337.

64. Spadaro S, Dalla Corte F, Scaramuzzo G, Grasso S, Cinnella G, Rosta V, et al. Circulating Skeletal Troponin During Weaning From Mechanical Ventilation and Their Association to Diaphragmatic Function: A Pilot Study. Front Med-Lausanne. 2021;8.

65. Thabet D, Makhlouf HA, Hasan AA, Ghanem MA. Are diaphragmatic indices assessed by ultrasound good predictors of weaning outcome in mechanically ventilated respiratory patients? The Egyptian Journal of Chest Diseases and Tuberculosis. 2020;69(4):681-7.

66. Theerawit P, Eksombatchai D, Sutherasan Y, Suwatanapongched T, Kiatboonsri C, Kiatboonsri S. Diaphragmatic parameters by ultrasonography for predicting weaning outcomes. Bmc Pulm Med. 2018;18(1):175.

67. Vivier E, Muller M, Putegnat JB, Steyer J, Barrau S, Boissier F, et al. Inability of Diaphragm Ultrasound to Predict Extubation Failure: A Multicenter Study. Chest. 2019;155(6):1131-9.

68. Xu Q, Yang X, Qian Y, Hu C, Lu W, Cai S, et al. Comparison of assessment of diaphragm function using speckle tracking between patients with successful and failed weaning: a multicentre, observational, pilot study. Bmc Pulm Med. 2022;22(1):459.

69. Yoo JW, Lee SJ, Lee JD, Kim HC. Comparison of clinical utility between diaphragm excursion and thickening change using ultrasonography to predict extubation success. Korean J Intern Med. 2018;33(2):331-9.

70. Zhang X, Yuan J, Zhan Y, Wu J, Liu B, Zhang P, et al. Evaluation of diaphragm ultrasound in predicting extubation outcome in mechanically ventilated patients with COPD. Ir J Med Sci. 2020;189(2):661-8.

71. Abdelwahed WM, Abd Elghafar MS, Amr YM, Alsherif SEI, Eltomey MA. Prospective study: Diaphragmatic thickness as a predictor index for weaning from mechanical ventilation. J Crit Care. 2019;52:10-5.

72. Asmita, Arshad Z, Siddiqui AK, Mourya R, Singh GP, Abbas H. Comparison of Ultrasound-based Diaphragmatic Thickness Fraction (DTF) with Rapid Shallow Breathing Index and DTF alone for Predicting Successful Weaning from Mechanical Ventilation: A Randomised Control Trial. J Clin Diagn Res. 2020;16(6):Uc67-Uc71.

73. Blumhof S, Wheeler D, Thomas K, McCool FD, Mora J. Change in Diaphragmatic Thickness During the Respiratory Cycle Predicts Extubation Success at Various Levels of Pressure Support Ventilation. Lung. 2016;194(4):519-25.

74. Cavus MA, Bektas SG, Sipahioglu H, Zararsiz GE, Turan S. Power of diaphragm ultrasonography to predict weaning success. Cukurova Med J. 2022;47(2):747-55.

75. DiNino E, Gartman EJ, Sethi JM, McCool FD. Diaphragm ultrasound as a predictor of successful extubation from mechanical ventilation. Thorax. 2014;69(5):423-7.

76. Dres M, Similowski T, Goligher EC, Pham T, Sergenyuk L, Telias I, et al. Dyspnoea and respiratory muscle ultrasound to predict extubation failure. Eur Respir J. 2021;58(5).

77. Genty T, Laverdure F, Peyrouset O, Rezaiguia-Delclaux S, Thès J, Stéphan F. Extubation Failure Prediction by Echography of the Diaphragm After Cardiothoracic Surgery: The EXPEDIA Study. Respir Care. 2022;67(3):308-15.

78. Haaksma ME, Smit JM, Heldeweg M, Nooitgedacht JS, Atmowihardjo LN, Jonkman AH, et al. Holistic Ultrasound to Predict Extubation Failure in Clinical Practice. Respir Care. 2021;66(6):994-1003.

79. Kundu R, Baidya D, Anand R, Maitra S, Soni K, Subramanium R. Integrated ultrasound protocol in predicting weaning success and extubation failure: a prospective observational study. Anaesthesiol Intensive Ther. 2022;54(2):156-63.

80. Lalwani LK, Govindagoudar MB, Singh PK, Sharma M, Chaudhry D. The role of diaphragmatic thickness measurement in weaning prediction and its comparison with rapid shallow breathing index: a single-center experience. Acute Crit Care. 2022;37(3):347-54.

81. Pirompanich P, Romsaiyut S. Use of diaphragm thickening fraction combined with rapid shallow breathing index for predicting success of weaning from mechanical ventilator in medical patients. J Intensive Care. 2018;6:6.

82. Soliman SB, Ragab F, Soliman RA, Gaber A, Kamal A. Chest Ultrasound in Predication of Weaning Failure. Open Access Maced J Med Sci. 2019;7(7):1143-7.

83. Trifi A, Abdellatif S, Ben Lamine F, Abdennebi C, Touil Y, Ben Lakhal S. Ultrasound assessment of the diaphragm during the first days of mechanical ventilation compared to spontaneous respiration: a comparative study. Tunis Med. 2021;99(11):1055-65.

84. Vetrugno L, Orso D, Corradi F, Zani G, Spadaro S, Meroi F, et al. Diaphragm ultrasound evaluation during weaning from mechanical ventilation in COVID-19 patients: a pragmatic, cross-section, multicenter study. Respir Res. 2022;23(1):210.

85. Azeredo LM, Nemer SN, Barbas CS, Caldeira JB, Noé R, Guimarães BL, et al. The Integrative Weaning Index in Elderly ICU Subjects. Respir Care. 2017;62(3):333-9.

86. Chittock DR, Uusaro A, Russell JA, Walley KR. Stress test and gastric-arterial PCO2 measurement improve prediction of successful extubation. Crit Care Med. 2000;28(7):2313-9.

87. Fernandez R, Raurich JM, Mut T, Blanco J, Santos A, Villagra A. Extubation failure: diagnostic value of occlusion pressure (P0.1) and P0.1-derived parameters. Intensive Care Med. 2004;30(2):234-40.

88. Hurtado FJ, Berón M, Olivera W, Garrido R, Silva J, Caragna E, et al. Gastric intramucosal pH and intraluminal PCO2 during weaning from mechanical ventilation. Crit Care Med. 2001;29(1):70-6.

89. Liu Y, Wei LQ, Li GQ, Lv FY, Wang H, Zhang YH, et al. A decision-tree model for predicting extubation outcome in elderly patients after a successful spontaneous breathing trial. Anesth Analg. 2010;111(5):1211-8.

90. Montgomery AB, Holle RH, Neagley SR, Pierson DJ, Schoene RB. Prediction of successful ventilator weaning using airway occlusion pressure and hypercapnic challenge. Chest. 1987;91(4):496-9.

91. Okamoto K, Sato T, Morioka T. Airway occlusion pressure (P0.1)-a useful predictor for the weaning outcome in patients with acute respiratory failure. J Anesth. 1990;4(2):95-101.

92. Rivera L, Weissman C. Dynamic ventilatory characteristics during weaning in postoperative critically ill patients. Anesth Analg. 1997;84(6):1250-5.

93. Vallverdú I, Calaf N, Subirana M, Net A, Benito S, Mancebo J. Clinical characteristics, respiratory functional parameters, and outcome of a two-hour T-piece trial in patients weaning from mechanical ventilation. Am J Respir Crit Care Med. 1998;158(6):1855-62.

94. Liu L, Liu H, Yang Y, Huang Y, Liu S, Beck J, et al. Neuroventilatory efficiency and extubation readiness in critically ill patients. Crit Care. 2012;16(4):R143.

95. Muttini S, Villani PG, Trimarco R, Bellani G, Grasselli G, Patroniti N. Relation between peak and integral of the diaphragm electromyographic activity at different levels of support during weaning from mechanical ventilation: a physiologic study. J Crit Care. 2015;30(1):7-12.

96. Dres M, Demoule A. Beyond Ventilator-induced Diaphragm Dysfunction: New Evidence for Critical Illness-associated Diaphragm Weakness. Anesthesiology. 2019;131(3):462-3.

97. Qing Q, Liang M, Sun Q, Xie B, Yang C, Liang W, et al. Using twitch tracheal airway pressure, negative inhale forced pressure, and Medical Research Council score to guide weaning from mechanical ventilation. J Thorac Dis. 2018;10(7):4424-32.

98. Castro AA, Cortopassi F, Sabbag R, Torre-Bouscoulet L, Kümpel C, Ferreira Porto E. Respiratory muscle assessment in predicting extubation outcome in patients with stroke. Arch Bronconeumol. 2012;48(8):274-9.

99. Xu Q, Yang X, Qian Y, Hu C, Lu W, Cai S, et al. Speckle tracking quantification parasternal intercostal muscle longitudinal strain to predict weaning outcomes: a multicentric observational study. Shock. 2023;59(1):66-73.

100. Takwoingi Y DN, Schiller I, Rücker G, Jones HE, Partlett C, Macaskill P. Chapter 10: Undertaking meta-analysis. Draft version (4 October 2022) for inclusion in: Deeks JJ, Bossuyt PM, Leeflang MM, Takwoingi Y, editor(s). Cochrane Handbook for Systematic Reviews of Diagnostic Test Accuracy Version 2. London: Cochrane. 2022.
